# PRMT1-mediated H4R3me2a recruits SMARCA4 to promote colorectal cancer progression by enhancing EGFR signaling

**DOI:** 10.1186/s13073-021-00871-5

**Published:** 2021-04-14

**Authors:** Bing Yao, Tao Gui, Xiangwei Zeng, Yexuan Deng, Zhi Wang, Ying Wang, Dongjun Yang, Qixiang Li, Peipei Xu, Ruifeng Hu, Xinyu Li, Bing Chen, Jin Wang, Ke Zen, Haitao Li, Melissa J. Davis, Marco J. Herold, Hua-Feng Pan, Zhi-Wei Jiang, David C. S. Huang, Ming Liu, Junyi Ju, Quan Zhao

**Affiliations:** 1 The State Key Laboratory of Pharmaceutical Biotechnology, Department of Hematology, the Affiliated Drum Tower Hospital of Nanjing University Medical School, China-Australia Institute of Translational Medicine, School of Life Sciences, Nanjing University, 163 Xianlin Avenue, Nanjing, 210023 China; 2 Department of Medical Genetics, Nanjing Medical University, Nanjing, China; 3 Beijing Advanced Innovation Center for Structural Biology, Beijing Frontier Research Center for Biological Structure, Tsinghua-Peking Joint Center for Life Sciences, School of Medicine, Tsinghua University, Beijing, China; 4 The Walter and Eliza Hall Institute of Medical Research, Department of Medical Biology, University of Melbourne, Melbourne, VIC Australia; 5 Department of General Surgery, the Affiliated Hospital of Nanjing University of Chinese Medicine, Nanjing, China

**Keywords:** Transcription, Epigenomics, H4R3me2s, PRMT1, SMARCA4, Colorectal Cancer

## Abstract

**Background:**

Aberrant changes in epigenetic mechanisms such as histone modifications play an important role in cancer progression. PRMT1 which triggers asymmetric dimethylation of histone H4 on arginine 3 (H4R3me2a) is upregulated in human colorectal cancer (CRC) and is essential for cell proliferation. However, how this dysregulated modification might contribute to malignant transitions of CRC remains poorly understood.

**Methods:**

In this study, we integrated biochemical assays including protein interaction studies and chromatin immunoprecipitation (ChIP), cellular analysis including cell viability, proliferation, colony formation, and migration assays, clinical sample analysis, microarray experiments, and ChIP-Seq data to investigate the potential genomic recognition pattern of H4R3me2s in CRC cells and its effect on CRC progression.

**Results:**

We show that PRMT1 and SMARCA4, an ATPase subunit of the SWI/SNF chromatin remodeling complex, act cooperatively to promote colorectal cancer (CRC) progression. We find that SMARCA4 is a novel effector molecule of PRMT1-mediated H4R3me2a. Mechanistically, we show that H4R3me2a directly recruited SMARCA4 to promote the proliferative, colony-formative, and migratory abilities of CRC cells by enhancing EGFR signaling. We found that *EGFR* and *TNS4* were major direct downstream transcriptional targets of PRMT1 and SMARCA4 in colon cells, and acted in a PRMT1 methyltransferase activity-dependent manner to promote CRC cell proliferation. In vivo, knockdown or inhibition of PRMT1 profoundly attenuated the growth of CRC cells in the C57BL/6 J-Apc^Min/+^ CRC mice model. Importantly, elevated expression of PRMT1 or SMARCA4 in CRC patients were positively correlated with expression of EGFR and TNS4, and CRC patients had shorter overall survival. These findings reveal a critical interplay between epigenetic and transcriptional control during CRC progression, suggesting that SMARCA4 is a novel key epigenetic modulator of CRC. Our findings thus highlight PRMT1/SMARCA4 inhibition as a potential therapeutic intervention strategy for CRC.

**Conclusion:**

PRMT1-mediated H4R3me2a recruits SMARCA4, which promotes colorectal cancer progression by enhancing EGFR signaling.

**Supplementary Information:**

The online version contains supplementary material available at 10.1186/s13073-021-00871-5.

## Background

Colorectal cancer (CRC) is the third most commonly diagnosed malignancy and the fourth leading cause of cancer-related deaths in the world; its burden is expected to increase by 60% to more than 2.2 million new cases and 1.1 million cancer deaths by 2030 [1]. One of the fundamental processes driving the initiation and progression of CRC is the accumulation of a variety of genetic and epigenetic changes in colon epithelial cells [2]. Over the past decade, major advances have been made in our understanding of cancer epigenetics, particularly regarding epigenetic alterations including aberrant DNA methylation and alterations in histone modification states [3–5]. For example, aberrant hypermethylation has been identified in the promoter regions of key tumor-suppressor genes, including *MLH1*, *CDKN2A*, and *APC* in the case of CRC; abnormal histone methylations, including H4K20me3, H3K4me1/2/3, H3K9me3, H3K27me3, and H3K79me2, have been frequently found in CRC tumor samples and cell lines [6]. Progress in this field suggests that these epigenetic alterations will be commonly used in the near future to direct the prevention and treatment of CRC [7].

Protein arginine methyl transferase 1 (PRMT1), a member of the protein arginine methyltransferase family (PRMTs), is the most abundant PRMT in mammals. PRMT1 mainly catalyzes asymmetric dimethylation of histone H4 on arginine 3 (H4R3me2a), usually a marker of transcriptional activation, which has been implicated in transcriptional control, pre-mRNA splicing, protein stability, DNA damage signaling, and cell fate decisions [8]. To date, only TDRD3 has been identified as a “reader” of epigenetic mark H4R3me2a [9]. PRMT1 is also involved in the interaction of many transcription factors and promoters, and the overexpression and abnormal splicing of PRMT1 directly affects the occurrence of tumors, including breast cancer, lung cancer, bladder cancer, leukemia, and colorectal cancer [10–14]. In colorectal cancer, the pathophysiological function of PRMT1 is largely unknown although PRMT1 has been examined as a marker of unfavorable prognosis for colon cancer patients [8, 14, 15].

SWI/SNF related matrix-associated actin-dependent regulator of chromatin subfamily A member 4 (SMARCA4, also known as Brahma-related gene 1, Brg1) is a catalytic subunit of the SWI/SNF complex, which uses ATP hydrolysis to provide energy required for the mediating changes in chromosome structure or chromatin remodeling [16]. SMARCA4 is indispensable for embryonic development and has been implicated in a variety of biological processes in both normal and neoplastic tissues [17, 18]. Heterozygous loss of *SMARCA4* in mice leads to increased risk of developing cancer including lung and mammary gland, indicating that it is a tumor-suppressor gene [18]. In support of this, the loss or mutation of *SMARCA4* is associated with human cancers including lung cancer, hepatocellular carcinoma, ovarian cancer, endometrial tumors, and Burkitt’s lymphoma [16, 19–25]. Other studies, however, have reported a tumor-promoting role of *SMARCA4* [26–28]. Thus, *SMARCA4* can be an oncogene for some cancers or in other contexts.

In the murine small intestinal epithelium, SMARCA4 is required for stem cell maintenance [29]. Loss of SMARCA4 attenuates Wnt signaling and prevents Wnt-dependent tumorigenesis in the murine small intestine [30]. In addition, duodenal SMARCA4 loss in mice is associated with dysregulation of the Notch pathway, which contributes to growth impairment, early death, and abnormal villous formation [31]. However, a recent study showed that SMARCA4 attenuates colonic inflammation and tumorigenesis through autophagy-dependent oxidative stress sequestration [32]. Thus, discrepancies abound regarding the functions of SMARCA4 with respect to tumor initiation and progression in gut. The roles of SMARCA4 in colon also remain unclear.

Here, we found that PRMT1 and SMARCA4 act cooperatively to promote CRC progression. Our data indicate that SMARCA4 is a novel effector molecule of PRMT1-mediated H4R3me2a. We show that *TNS4* and *EGFR* are direct downstream targets of PRMT1/SMARCA4 mediating activation of EGFR signaling pathway in CRC. These findings provide a novel strategy for targeting therapy of CRC.

## Methods

### Cell lines, transfections, and siRNA interference assays

Two colon carcinoma cell lines (HCT116 and SW620) were purchased from the Shanghai Institute of Cell Biology, Chinese Academy of Sciences (Shanghai, China). Cells were maintained at 37 °C in a humidified air atmosphere containing 5% carbon dioxide in McCOY’s 5A medium (HCT116) or RPMI-1640 (SW620) supplemented with 10% FBS (Invitrogen). The human colon cancer cell lines were recently authenticated by Genetic Testing Biotechnology Corporation (Suzhou, China) using short tandem repeat (STR) profiling. All lines were found to be negative for mycoplasma contamination. SiRNA against *PRMT1* and *SMARCA4* were synthesized by Jima, China. The sequences used were as follows: *PRMT1* siRNA-1, 5′-CUGAAGUCCAGGUCGAUGGUGAAGUC-3′; *PRMT1* siRNA-2, 5′-UUGUAGUCUUUGUACUGCC-3′; *SMARCA4* siRNA-1, 5′-UCGCUUUGGUUCGCAAAUC-3′; *SMARCA4* siRNA-2, 5′-UUCCUCCUCAUUCAGGUCC-3′. HCT116 and SW620 cells were transfected with oligonucleotides or the indicated constructs using Lipofectamine 3000 (Invitrogen) according to the manufacturer’s instructions.

### Mass spectrometry

High-capacity streptavidin agarose (Thermo Scientific) was incubated with C-terminal biotin-tagged 20 amino acid N-terminal peptides of H4, H4R3me2a, and H4R3me2s peptide immunoprecipitates from nuclear extracts of HCT116 cells, separated by sodium dodecyl sulfate-polyacrylamide gel electrophoresis (SDS-PAGE), and stained with SimplyBlue Safestain (Invitrogen). Protein bands of interest were excised and subjected to electrospray–ion trap tandem mass spectrometry (LCQ-Deca, Finnigan).

### Plasmid construction, recombinant protein expression, and purification

pGEX-6p-1 plasmids encoding GST, GST-SMARCA4-F1 (aa1-351), GST-SMARCA4-F2 (aa352-707), GST-SMARCA4-F3 (aa708-1008), GST-SMARCA4-F4 (aa1009-1314), and GST-SMARCA4-F5 (aa1315-1647) (human SMARCA4 isoform 1, Accession AAG24789.1) were transformed into *E. coli* BL21 and cultured with IPTG at 16 °C for 12 h until the optical density (OD600) reached 0.5~0.6. BL21 cells were collected and sonicated in cold PBS, and GST-fusion proteins were purified with Glutathione S-transferase beads according to the users’ manual. Purity was assessed by SDS-PAGE.

Wild-type human *PRMT1* coding regions was cloned into the retroviral vector plasmid MSCV and confirmed by DNA sequencing. The deletion mutant *PRMT1Δ* (GSGTG, amino acids 86–90) [33] was constructed by site-directed mutagenesis. The oligonucleotide used to introduce the deletion was 5′-GGTGGTGCTGGACGTC ATCCTCTGCATGTTTGC-3′.

### Microscale thermophoresis (MST) analysis and isothermal titration calorimetry (ITC) assays

For MST analysis, purified recombinant SMARCA4-F4 (aa1009-1314) proteins or Flag-SMARCA4 from HCT116 cells were labeled with Monolith NT-647-NHS. Labeled proteins were used at a concentration of 100 nM in PBS pH 7.4 containing 0.05% Tween-20. The concentration of H4, H4R3me2a, and H4R3me2s peptides ranged from 10 nM to 500 μM. The combined solutions of labeled proteins and peptides were incubated for 5 min and transferred into silicon-treated capillaries. Thermophoresis was measured for 30 s on a NanoTemper Monolith NT.115 (NanoTemper Technologies GMBH) using 60% LED power and 20% laser power. Dissociation constants were calculated by NanoTemper Analysis 1.5.41 software using the mass action equation (Kd formula).

A MicroCal ITC-200 system (Malvern Instruments Ltd.) was used for ITC experiments. Briefly, the synthesized peptides (Genscript, Nanjing, China) and proteins were all subjected to extensive dialysis against PBS. Peptides at concentrations of 1 mM were loaded into the ITC syringe, and proteins at concentrations of 100 μM were loaded into the ITC cell. Then, 19 injections of 2 μl peptide each were automatically made into the cell at 25 °C. The results of binding isotherms were analyzed using the Origin 7.0 software package (Origin Lab).

### Immunofluorescence and confocal microscopy

HCT116 cells were fixed with 4% formaldehyde for 15 min at room temperature. After washing cells 3 times in PBS with 0.1% Triton X-100, cells were blocked with 4% BSA for 30 min. Cells were incubated with primary antibody (PRMT1 and SMARCA4) for 1 h at room temperature. Following washes with PBS 0.1% Triton X-100, cells were incubated with a secondary antibody (Goat anti-Mouse IgG Alexa Fluor 488 and Goat anti-Rabbit IgG Alexa Fluor 594 from Life Technologies) for 1 h at room temperature. Following washes with PBS 0.1% Triton X-100, cells were stained with DAPI (Sigma) and visualized by confocal scanning microscopy (Olympus FV10i).

### Western blot analysis and protein interaction studies

Cellular proteins were extracted by RIPA lysis buffer at high salt concentration (420 mM NaCl), and western blot analysis was performed as described previously [34]. Scans of the uncropped blots for Western blots are presented in Additional file 3. For immunoprecipitation assays, cells were washed with cold phosphate buffered saline (PBS) and lysed with cold cell lysis buffer for 30 min at 4 °C. Then, 500 μg of cellular extract was incubated with appropriate specific antibodies or normal rabbit immunoglobin G (IgG) at 4 °C overnight with constant rotation, followed by the addition of protein A/G Sepharose beads and incubation for 2 h at 4 °C. Beads were then washed five times with cell lysis buffer (50 mM Tris-HCl, pH 7.4, 150 mM NaCl, 1 mM EDTA, 0.5% NP-40, 0.25% sodium deoxycholate and protease inhibitor mixture). The immune complexes were subjected to SDS-PAGE followed by immunoblotting with secondary antibodies.

### RNA isolation and quantitative RT-PCR

Total RNA from cultured cells was extracted using TRIzol reagent (Invitrogen). cDNAs were synthesized with a HiScript 1st Strand cDNA Synthesis Kit (Vazyme Biotech, China). Quantitative RT-PCR was performed using a FastStart Universal SYBR Green Master (Vazyme Biotech, China) according to the manufacturer’s instructions in a StepOnePlus™ Real-Time PCR System (Thermo Scientific) in a final volume of 20 μl. Cycling conditions were 94 °C for 15 s, 60 °C for 1 min, and 72 °C for 30 s. Each reaction was performed in triplicate. The primer sequences for RT-PCR are listed in Additional file 2: Table S5.

### Proliferation assay, EdU incorporation assay, colony formation assay, and migration assays

The in vitro viability of colorectal cancer cells was assessed using the Cell Counting Kit-8 (CCK-8). Cell proliferation was determined by incorporation of 5-ethynyl-20-deoxyuridine (EdU) using an EdU Cell Proliferation Assay Kit (Ribobio). Colony formation was observed by staining cells with 0.1% crystal violet (Sangon Biotechnologies Inc., China). For cell migration assays, 5 × 10^5^ cells were seeded into the upper chamber of the Transwell apparatus (Corning Costar) in serum-free medium, and medium supplemented with 10% FBS was added to the bottom chamber. After 24 h, the cells on the upper surface that did not pass through the 8-μm pore-size polycarbonate filter were removed using a moistened cotton swab; the cells migrating to the lower membrane surface were fixed in 100% methanol for 10 min, stained with 0.4% crystal violet for 15 min, and counted under a microscope (Nikon) at × 100 magnification.

### Gene-microarray analysis

Total RNA was extracted from PRMT1-NC1, PRMT1-NC2, PRMT1-KD1, PRMT1-KD2, SMARCA4-KD1, and SMARCA4-KD2 HCT116 cell lines. Total RNA was quantified by the NanoDrop ND-2000 (Thermo Scientific), and RNA integrity was assessed using an Agilent Bioanalyzer 2100 (Agilent Technologies). Sample labeling, microarray hybridization, and washing were performed based on the manufacturer’s standard protocols. Briefly, total RNA were transcribed to double strand cDNA, then synthesized into cRNA and labeled with Cyanine-3-CTP. The labeled cRNAs were hybridized onto the microarray. After washing, the arrays were scanned by the Agilent Scanner G2505C (Agilent Technologies). The 60-mer oligo nucleotide probes were designed using a microarray (Agilent) and performed by Oe-biotech (Shanghai, China). For the study of differential gene expression, Genespring (version13.1, Agilent Technologies) were employed to complete the basic analysis with the raw data. The genes with a fold change value greater than 1.5, and a *p* value < 0.01 was considered differentially expressed. Relationships of differentially expressed genes were determined by GO and GSEA analysis.

### Chromatin immunoprecipitation (ChIP)

ChIP assays were performed with HCT116 cells in accordance with standard protocols as described previously [35]. Normal rabbit IgG served as the control. ChIP samples were analyzed by quantitative real-time PCR using the FastStart Universal SYBR Green Master (Vazyme Biotech, China). The primer sequences for ChIP are listed in Additional file 2: Table S6.

### ChIP-Seq data processing and analysis

The ChIP-Seq data was from CistromeDb [36] about H3K4me1 [37], H3K4me3 [38], H3K27ac [39], and SMARCA4 [40]. CistromeDb provides a QC metric with 7 QC items across three different layers. For datasets with replicates, we choose the ChIP-Seq data with the highest QC score. All data uses the standard analysis pipeline ChiLin [41] to process all chromatin profiling reads. The ChIP and control FASTQ data were mapped onto a genome with BWA [42]. Marks on TNS4 and EGFR were distinctly identified using MACS2 [43] for narrow peak calling mode.

### ATAC-seq data processing and analysis

Chromatin accessibility data by ATAC-seq for SMARCA4 was from GSM2719724 [44]. Reads were aligned to the human genome (hg38) using STAR [45]. Only reads that mapped to a unique genomic locations (MAPQ > 10) were used for downstream analysis. ATAC-seq peaks were found using the findPeaks program in HOMER [46].

### Clinical samples and IHC staining

Ninety pairs of CRC and adjacent normal paraffin tissue sections (HColA180Su14) were obtained from Shanghai Outdo Biotech (National Human Genetic Resources Sharing Service Platform with code No. 2005DKA21300, Shanghai, China) under the approval by the Ethics Committee of Taizhou Hospital, Zhejiang, China. Immunohistochemical staining (IHC) was performed using paraffin-embedded sections of biopsies from CRC patients and controls according to standard protocols (Cell Signaling Technology). Briefly, slides were incubated with primary antibodies: anti-SMARCA4 (1:100 dilution, Abcam, ab110641), anti-PRMT1 (1:100 dilution, CST, #2449), anti-EGFR (1:200 dilution, Abcam, ab52894), or anti-TNS4 (1:100 dilution, Abcam, ab192247), followed by incubation with horseradish peroxidase-conjugated goat anti-rabbit secondary antibody. Antibody binding was visualized using a 2-Solution DAB Kit (Invitrogen). All colorectal cancer tissue sections were reviewed by two experienced pathologists, and staining of SMARCA4, PRMT1, TNS4, or EGFR in the tissue was scored independently (using the H-score system [47]) by two pathologists blinded to the clinical data. Rare discordant scores were resolved by re-review of the slide and consultation between the pathologists. The intensity of immunostaining (category A) was documented as 0–3: 0, negative; 1, weak; 2, moderate; 3, strong. For the Pearson correlation scatter plot of molecules in CRC, the H score was calculated by adding the multiplication product of the different staining intensities in category A (0–3) with the percentage of positive cells, i.e., H score (0–300 scale) = 3 × (% at 3+) + 2 × (% at 2+) + 1 × (% at 1+). The clinical features of the patients are listed in Additional file 2: Table S1 (the same patients from which tissues were obtained). For survival analyses, patient overall survivals were stratified by expression of the gene of interest and were presented as Kaplan–Meier plots and tested for significance using log-rank tests. Degree of correlation between SMARCA4, PRMT1, TNS4, and EGFR expression patterns in CRC was assessed via Pearson correlation analysis.

### Mice, colitis-associated colon tumor formation, and treatment

C57BL/6 J-Apc^Min/+^ mice were purchased from the Model Animal Resource Information Platform (Nanjing, China). All animal experimental procedures were conducted in accordance with animal protocols approved by the Laboratory Animal Center of Nanjing University.

Colitis-associated colon tumor formation was induced in mice as described previously [48]. Briefly, 8-week-old male mice were provided with drinking water containing 2% dextran sodium sulfate (DSS) (MP Biomedicals, Irvine, USA) for 7 consecutive days, followed by another 21 days with 45 kcal% high-fat diet (SYSE Biotech, Changzhou, China). This cycle was repeated once after high-fat diet for 4 weeks until day 150, when all mice were euthanized for analysis. Lentivirus expressing murine PRMT1 shRNA (5′-GTCAAAGCCAACAAGTTA-3′) was administrated into C57BL/6J-Apc^Min/+^ mice via enema infection from day 64 once a week (1 × 10^8^ TU). AMI-1 (Apexbio, Boston, USA) was administrated intraperitoneally into C57BL/6J-Apc^Min/+^ mice at 10 mg/kg twice weekly from day 64. Cetuximab (MedChemExpress, Shanghai, China) was injected in the tail vein into C57BL/6J-Apc^Min/+^ mice at 30 mg/kg once a week from day 120.

### Statistical analysis

All data were collected from more than 3 independent experiments. Results are expressed as the mean ± standard deviation unless otherwise indicated and were analyzed using GraphPad Prism 5.0 software (GraphPad Software, San Diego, CA). Statistically significant differences were examined using Two-tailed Student’s *t* test, two-sided Pearson *χ*^2^ test, or the log-rank (Mantel-Cox) test to derive the significance of the differences between two groups. *P* < 0.05 was considered to be significant.

## Results

### SMARCA4 is a major H4R3me2a-associated protein and promotes CRC cell proliferation

**Fig. 1:**
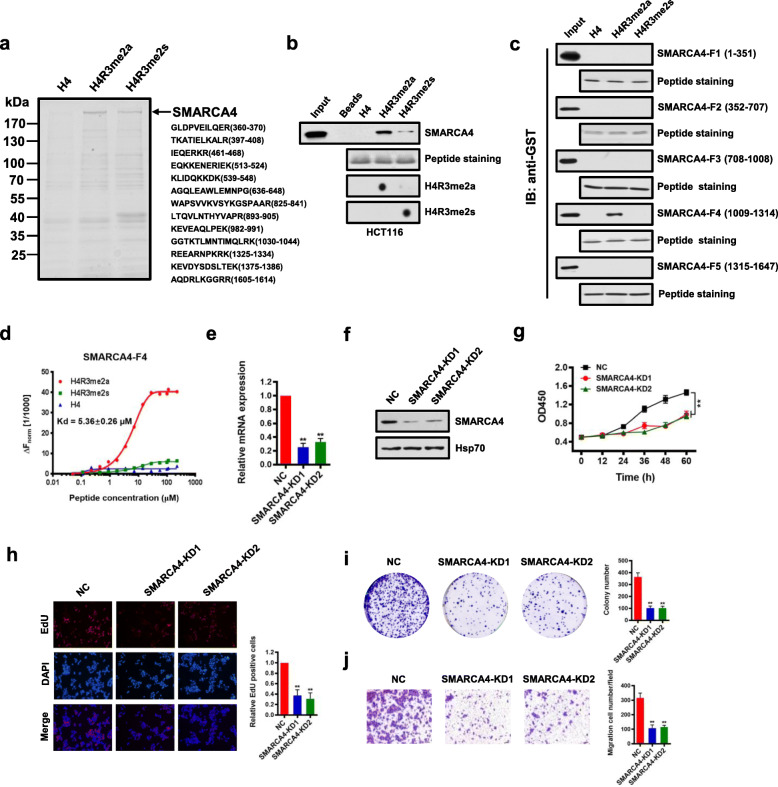
SMARCA4 is a major H4R3me2a-associated protein and promotes CRC cell proliferation. **a** Immunoaffinity purification to identify proteins that are associated with H4R3me2a. The protein bands were retrieved and analyzed by mass spectrometry. SMARCA4 peptide fragments identified by mass spectrometric assay (right). **b** Peptide pull-down assay to detect the interactions between H4, H4R3me2a, and H4R3me2s peptides and SMARCA4 in HCT116 cell nuclear extracts (top panel). Coomassie staining shows equivalent loading of the three peptides (middle panel). The modification of the synthesized peptide was confirmed by dot blot analysis with specific antibodies (bottom panels). **c** Peptide pull-down experiments were performed with H4, H4R3me2a, and H4R3me2s peptides and purified recombinant glutathione S-transferase (GST) fusion proteins of SMARCA4 fragments expressed in *E. coli.* as in **b**. **d** MST assay to identify direct interactions between SMARCA4-F4 and H4R3me2a peptides. The dissociation constant (Kd) between SMARCA4-F4 and H4R3me2a peptide is 5.36 ± 0.26 μM. **e**, **f** Identification of the effect of SMARCA4 knockdown (SMARCA4-KD) in HCT116 cells by quantitative real-time PCR (**e**) and western blot analysis with indicated antibodies (**f**). Hsp70 served as a loading control. **g** Proliferation of HCT116 cells following knockdown of SMARCA4. Values at the indicated time points represent mean ± s.d. from three independent tests; **P < 0.01. **h** EdU proliferation analysis of the effect of siRNA knockdown of SMARCA4 on the growth of HCT116 cells. Representative images (left panel) and quantitative analyses of the assay (right panel) are shown. **i** Colony formation assay of HCT116 cells following SMARCA4 knockdown. Representative images (left panel) and quantitative analyses of the colony formation (right panel) are shown. **j** Migration assay of HCT116 cells following SMARCA4 knockdown. The numbers of migrated cells were quantified by counting the numbers of cells in entire fields at ×200 magnification. Representative images (left panel) and quantitative analyses of the migrated cells (right panel) are shown. For **e**, **h**, **i,** and **j**, results are shown as mean ± s.d. from three independent experiments; **P < 0.01 compared with the scrambled negative control (NC)

To identify proteins that bind to histone H4R3me2a in colon cells, we employed affinity purification followed by mass spectrometric analysis to identify proteins that could potentially bind H4R3me2a. We performed a peptide pull-down assay using C-terminal biotin-tagged 20 amino acid N-terminal peptides of histone H4 in which the Arg3 residue was either asymmetrically dimethylated (H4R3me2a), or symmetrically dimethylated (H4R3me2s), or nonmethylated (H4). We incubated equivalent amounts of each peptide coupled to streptavidin beads with nuclear extracts from HCT116 cells, washed the beads, separated the eluates on SDS-PAGE gels, and stained with SimplyBlue Safestain (Fig. 1a, left). Compared to H4R3me2s or H4 peptides, we found that there was a protein band above 170 kDa that preferentially bound H4R3me2a. Mass spectrometric analysis showed that this protein band corresponded to SMARCA4 (Fig. 1a, right). Analysis of other proteins identified by mass spectrometry will be described elsewhere (Additional file 2: Table S9). We confirmed the interaction between H4R3me2a and SMARCA4 from HCT116 cell nuclear extracts by immunoblot using an antibody to SMARCA4 following a peptide pull-down assay. Strong binding of SMARCA4 was observed with the H4R3me2a peptide, but not with the unmethylated or symmetrically methylated peptides (Fig. 1b). We further found that this interaction was direct, as demonstrated by the peptide pull-down assay using purified recombinant glutathione S-transferase (GST) fusion proteins of SMARCA4 fragments expressed in *E. coli*. Binding required the residues between aa1009 and aa1314, a region which contains the HELICc domain [49] of SMARCA4 (SMARCA4-F4, Fig. 1c, Additional file 1: Fig. S1a and S1b). No binding to H4R3me2a peptides by other SMARCA4 domains, including a fragment containing a Bromo domain, was detected (Fig. 1c, Additional file 1: Fig. S1a and S1b). Similarly, H4R3me2a peptides bound robustly with purified Flag-tagged SMARCA4 from HCT116 cells, but not with the unmethylated or symmetrically methylated peptides (Additional file 1: Fig. S1c-d).

Next, we confirmed that H4R3me2a peptide was directly bound by SMARCA4-F4 by microscale thermophoresis (MST) using purified recombinant SMARCA4-F4 protein. The data fit a one-site-binding model with a dissociation constant (Kd) of 5.36 ± 0.26 μM for SMARCA4-F4 binding to H4R3me2a peptide. No binding of SMARCA4-F4 to H4 or H4R3me2s control peptides was observed (Fig. 1d). Keeping in line with this, we obtained a similar Kd of 6.27 ± 0.37 μM for purified Flag-SMARCA4 from HCT116 cells binding to H4R3me2a peptide (Additional file 1: Fig. S1e). Isothermal titration calorimetry (ITC) measurements also showed a similar specific interaction of SMARCA4-F4 with H4R3me2a peptide, with a Kd of 2.41 ± 0.12 μM (Additional file 1: Fig. S2). Again, compared to H4R3me2a, no interaction of SMARCA4-F4 with H4 or H4R3me2s control peptides was detected (Additional file 1: Fig. S2). These results indicate that SMARCA4 can discriminate between H4R3me2a and H4R3me2s marks and binds strongly to the isolated methyl mark of H4R3me2a compared to H4R3me2s.

We noticed that histone mark H4R3me2a was mainly triggered by PRMT1 in cells [8]. Thus, we reasoned that SMARCA4 and PRMT1 could interact in cells. Immunofluorescence staining experiments showed that SMARCA4 co-localized with PRMT1 in the nucleus of HCT116 cells (Additional file 1: Fig. S3a). We analyzed Flag-antibody immunoprecipitates from HCT116 cells overexpressing Flag-tagged PRMT1 by immunoblot with antibodies to SMARCA4 and found that PRMT1 co-immunoprecipitated with SMARCA4 (Additional file 1: Fig. S3b). In addition, we showed that SMARCA4 was co-immunoprecipitated with endogenous PRMT1 (Additional file 1: Fig. S3c). Furthermore, we confirmed that PRMT1 interacted with SMARCA4 at gene promoters in HCT116 cells using a ChIP-reChIP strategy (Additional file 1: Fig. S3d). These results indicate that SMARCA4 and PRMT1 interact in colon cells. However, whether they interact directly needs to be determined.

We went on to determine potential roles of SMARCA4 in CRC cells. To examine the effect of SMARCA4 on cell growth, we knocked down SMARCA4 in HCT116 cells using two independent RNAi. We found that in SMARCA4-knockdown (SMARCA4-KD) cells, levels of *SMARCA4* mRNAs were reduced to ~ 30% of levels in cells transfected with the scrambled negative control (NC) (Fig. 1e). The reduced protein levels of SMARCA4 were confirmed by western blot analysis (Fig. 1f). Knockdown of SMARCA4 significantly reduced the cell growth rate in HCT116 cells (Fig. 1g). These results were confirmed by EdU staining to detect nucleotide analogue incorporation into replicated DNA (Fig. 1h). In addition, knockdown of SMARCA4 significantly reduced the numbers of HCT116 colonies formed after culture compared with NC controls (Fig. 1i). Results from the Transwell assay showed that cell migratory capabilities of HCT116 cells were also significantly reduced in SMARCA4 knockdown cells compared with the NC cells (Fig. 1j). These data indicate that SMARCA4 is an H4R3me2a-associated protein, and knockdown of SMARCA4 inhibits CRC cell proliferation.

### PRMT1 expression is associated with prognosis of CRC, and knockdown of PRMT1 reduces CRC cell proliferation

**Fig. 2:**
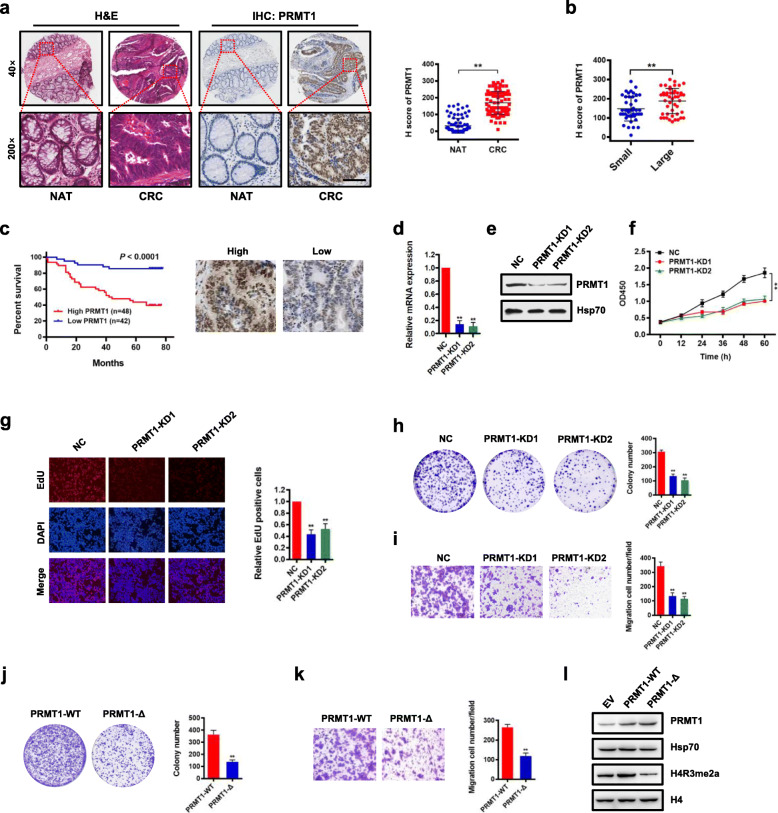
Upregulation of PRMT1 in colorectal cancer is associated with poor prognosis, and knockdown of PRMT1 reduces CRC cell proliferation. **a** Hematoxylin and eosin (H&E) staining and immunohistochemical staining (IHC) of PRMT1 protein in adjacent normal colon tissue controls (NAT) and colorectal cancer (CRC) human tissues. Representative micrographs are shown in original magnification (× 200) as indicated (left); total IHC score of PRMT1 in NAT and CRC tissues (*n* = 90); **P < 0.01 (right). Scale bar, 50 μm. **b** Correlation of PRMT1 expression with tumor size; **P < 0.01. **c** Kaplan–Meier plot of overall survival of 90 patients with colorectal cancer, stratified by PRMT1 expression. Log-rank test, *P* < 0.0001. **d**, **e** Effect of PRMT1 knockdown (PRMT1-KD) in HCT116 cells assessed by quantitative real-time PCR (**d**) and western blot analyses with the indicated antibodies (**e**). Hsp70 served as a loading control. **f** Proliferation of HCT116 cells following PRMT1 knockdown. Values at the indicated time points represent mean ± s.d. from three independent tests; **P < 0.01. **g** EdU proliferation analysis of the effect of PRMT1-KD on the growth of HCT116 cells compared with NC controls; Representative images (left panel) and quantitative analyses of the assay (right panel) are shown. **h** Colony formation assay of HCT116 cells following PRMT1 knockdown. Representative images (left panel) and quantitative analyses of the colony formation (right panel) are shown. **i** Migration assays of HCT116 cells following PRMT1 knockdown. The numbers of migrated cells were quantified by counting the numbers of cells in entire fields at × 200 magnification. Representative images (left panel) and quantitative analyses of the migrated cells (right panel) are shown. **j**, **k** Colony formation (**j**) and migration assays (**k**) of HCT116 cells overexpressing wild-type PRMT1 (PRMT1-WT) or PRMT1-Δ. **l** Western blot analysis of indicated proteins from HCT116 cells overexpressing PRMT1-WT, PRMT1-Δ, or EV (empty vector, MSCV). Hsp70 and histone H4 served as loading controls. Data are representative of three independent experiments. All results are shown as mean ± s.d. from three independent experiments; **P < 0.01 compared with the NC control

Histone modification of H4R3me2a in cells is mainly mediated by PRMT1 [50]. Thus, we evaluated PRMT1 expression and its potential role in CRC cells. To investigate the clinical significance of PRMT1 expression in patients with CRC, we examined expression of PRMT1 by immunohistochemical staining (IHC) in a human CRC tissue array containing 90 CRC samples and adjacent normal colon tissue controls (Additional file 2: Table S1). We found that PRMT1 was significantly upregulated in CRC compared with matched adjacent normal colon tissues (NAT; Fig. 2a). Notably, PRMT1 expression correlated with increased tumor size and higher grade, but not with lymph node status or TNM stage (Fig. 2b, Additional file 2: Table S1). Importantly, Kaplan–Meier survival analysis showed that CRC patients with high PRMT1 expression had shorter overall survival (Fig. 2c). These results indicate that PRMT1 expression levels are upregulated in human CRC tissues and correlate with poor prognosis in CRC, suggesting that PRMT1 may promote cancer cell growth during malignant progression.

To determine the role of PRMT1 in CRC cell proliferation, two different short hairpin RNAs against PRMT1 were used to knock down PRMT1 expression in HCT116 cells. We found that *PRMT1* expression in PRMT1-knockdown (PRMT1-KD) cells was reduced to ~ 20% of that in cells transfected with the scrambled negative control (NC) (Fig. 2d). Decreased protein levels of PRMT1 were confirmed by western blot analysis (Fig. 2e). Knockdown of PRMT1 significantly reduced the cell growth rate of HCT116 cells (Fig. 2f). This was confirmed by EdU staining to detect nucleotide analogue incorporation into replicated DNA (Fig. 2g). In addition, knockdown of PRMT1 significantly reduced the numbers of HCT116 colonies formed after culture compared with NC cells (Fig. 2h). Results from the Transwell assay showed that cell migratory capabilities of HCT116 cells were also significantly reduced in PRMT1 knockdown cells compared with NC cells (Fig. 2i). These data indicate that knockdown of PRMT1 inhibits CRC cell proliferation in vitro.

To determine whether regulation of cell proliferation by PRMT1 was methyltransferase activity-dependent, we constructed a mutant form of PRMT1 in which five amino acids (GSGTG, aa86-90) located in the S-adenosyl-L-methionine binding motif were deleted (PRMT1-Δ) [51]. HCT116 cells were transfected with vectors overexpressing either PRMT1 or PRMT1-Δ. Cell colony formation assay and Transwell assay showed that cell proliferation of HCT116 cells was significantly reduced in PRMT1-Δ cells compared with the wild-type PRMT1 overexpressing cells (Fig. 2j, k), similar to the effect obtained from PRMT1 knockdown. The effect of the PRMT1-Δ mutant on histone H4 was confirmed by western blot analysis with an anti-H4R3me2a antibody (Fig. 2l). These results suggest that the methyltransferase activity of PRMT1 is critical for CRC cell proliferation in vitro.

### *TNS4* and *EGFR* are direct downstream transcriptional targets of PRMT1 and SMARCA4 in CRC cells

**Fig. 3:**
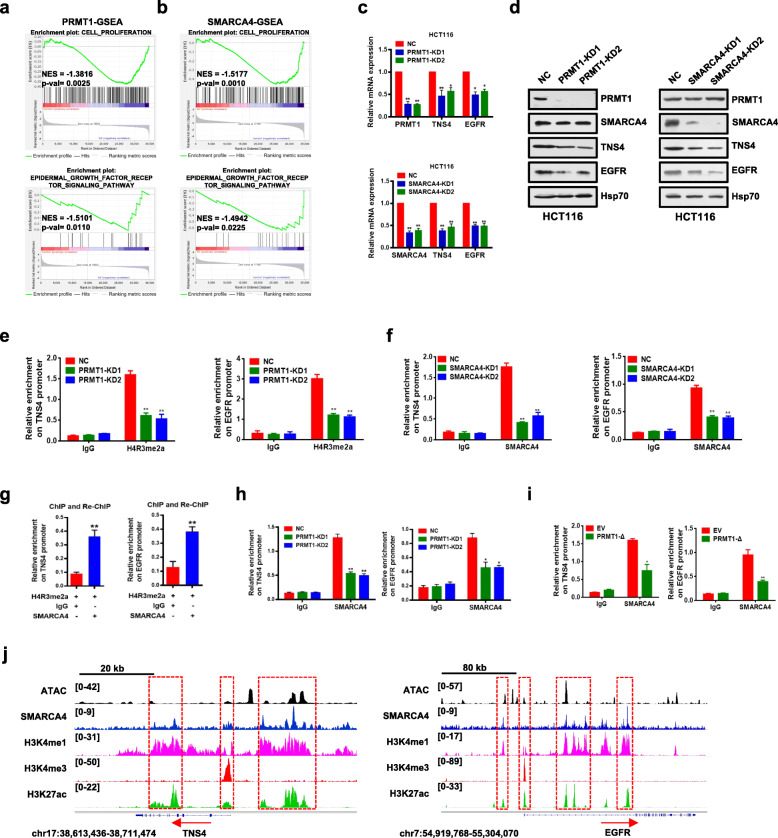
Identification of transcriptional targets of PRMT1 and SMARCA4. **a**,**b** Gene set enrichment analysis (GSEA) plots for cell proliferation-related and EGFR signaling pathway-related genes in HCT116 cells following PRMT1 knockdown (**a**) or SMARCA4 knockdown (**b**). **c** Quantitative real-time PCR analysis of *PRMT1*, *TNS4*, or *EGFR* mRNA levels normalized to GAPDH in scrambled negative control HCT116 cells (NC) and PRMT1-KD1/2 or SMARCA4-KD1/2 HCT116 cells. **d** Western blot analysis of indicated proteins in NC, PRMT-KD, and SMARCA4-KD HCT116 cells. Hsp70 served as a loading control. Data are representative of three independent experiments. **e**,**f** ChIP analysis of H4R3me2a and SMARCA4 binding to the *TNS4* and *EGFR* promoter in NC, PRMT-KD, and SMARCA4-KD HCT116 cells. **g** ChIP-reChIP analysis of chromatin from HCT116 cells. The first antibody (H4R3me2a) and second antibody (SMARCA4) used in the reChIP are shown below the bar plot. The amount recovered from the reChIP was determined by qPCR and is shown as a percentage of the input. **h** ChIP analysis of SMARCA4 on the *TNS4* and *EGFR* promoter from PRMT1 knockdown cells or NC control cells. **i** ChIP analysis of SMARCA4 on the *TNS4* and *EGFR* promoter from PRMT1-Δ-overexpressing cells or EV (empty vector, MSCV) control cells. **j** Genomic tracks of ATAC and ChIP intensities of SMARCA4, H3K4me1, H3K4me3, and H3K27ac in the vicinity of *TNS4* and *EGRF* loci in HCT116 cells. Track height is normalized to relative number of mapped reads. All results are shown as mean ± s.d. from three independent experiments. **P < 0.01 or *P < 0.05 compared to NC control or the indicated control (rabbit IgG)

The experiments above showed that PRMT1 and SMARCA4 promoted proliferation of CRC cells. To identify their potential downstream targets and understand their mechanisms of action, we conducted gene expression profiling assays with the Agilent SurePrint G3 Human Gene Expression v3 (8*60K, Design ID:072363) using mRNA following PRMT1 knockdown, SMARCA4 knockdown, and NC controls from HCT116 cells (Additional file 1: Fig. S4a and S4b). Gene set enrichment analysis (GSEA) showed that cell proliferation and EGFR signaling genes were enriched in PRMT1- or SMARCA4-knockdown HCT116 cells compared to NC controls (Fig. 3a, b). Consistently, gene ontology (GO) analysis of the differentially expressed genes in the PRMT1- or SMARCA4-knockdown versus NC controls revealed significant enrichment of genes involved in several key cellular processes such as EGFR signaling pathways, cell proliferation, and cell division or migration, which correlated with cancer progression (Additional file 1: Fig. S4c). From gene profile data, we found a total of 770 downregulated genes in PRMT1-KD1 and 781 downregulated genes in PRMT1-KD2 versus NC controls with at least a 1.5-fold change, whereas there were a total of 1967 downregulated genes in SMARCA4-KD1 and 1932 downregulated genes in SMARCA4-KD2 versus NC controls with at least a 1.5-fold change (Additional file 1: Fig. S4d, GEO database under accession GSE143198 and GSE143199). There were 109 common genes which exhibited at least 1.5-fold downregulation in all four KD profiles (Additional file 1: Fig. S4d; Additional file 2: Table S10). We found that 15 genes in a previously published SMARCA4 ChIP-Seq database [40] in HCT116 cells overlapped with our 109 genes (Additional files 1 & 2: Fig. S4e, Table S2). Among these 15 common target genes, TNS4 (also named CTEN), which regulates EGFR protein levels through a posttranslational mechanism and prolongs signaling by EGFR through reducing its ligand-induced degradation [52], turned out to be the most downregulated gene by both PRMT1 and SMARCA4 knockdown. Interestingly, we also found that EGFR was among the 15 overlapping gene list. Based on these findings, we chose *TNS4* and *EGFR* for further investigation as potential downstream target genes of PRMT1 and SMARCA4.

We confirmed that knockdown of PRMT1 or SMARCA4 in HCT116 cells led to significantly decreased expression of *TNS4* and *EGFR* at both transcriptional and protein levels (Fig. 3c, d). To probe whether PRMT1 or SMARCA4 directly regulated *TNS4* and *EGFR* transcription, we performed ChIP analysis using anti-PRMT1-mediated H4R3me2a or anti-SMARCA4 antibodies at *TNS4* and *EGFR* gene promoters. We found that knockdown of PRMT1 led to a significant reduction of H4R3me2a and knockdown of SMARCA4 led to a significant reduction of SMARCA4 at the promoters of *TNS4* and *EGFR* (Fig. 3e, f) in HCT116 cells, suggesting that PRMT1 or SMARCA4 bound to the promoters and directly regulate transcription of *TNS4* and *EGFR*. Further, we confirmed these proteins interacted on the promoters of TNS4 and EGFR by a ChIP-reChIP strategy, in which chromatin immunoprecipitated with H4R3me2a antibody was re-immunoprecipitated with antibody to SMARCA4 (Fig. 3g). Importantly, we found that enrichment of SMARCA4 on *TNS4* or *EGFR* promoter was significantly decreased (Fig. 3h, i, Fig. S8e) under these conditions, including when PRMT1 was knocked down, or when PRMT1-Δ was overexpressed in HCT116 cells, or when HCT116 cells were treated with AMI-1, a PRMT1 methyltransferase inhibitor [53]. These results indicate that SMARCA4 bound to H4R3me2a in cells. In control experiments, we did not see a change of SMARCA4 protein levels after knocking down PRMT1 (Fig. 3d, left).

Indeed, ChIP-Seq data demonstrated that SMARCA4 bound *TNS4* and *EGFR* gene sequences in colon HCT116 cells, and the enrichment pattern was similar to active promoter and enhancer marks H3K4me3, H3K4me1, and H3K27ac genome-wide (Cistrome Data Browser). Also the SMARCA4 enrichment was largely overlapped with ATAC-seq peaks at promoters, TSSs, and gene regions (Fig. 3j). Unfortunately, our H4R3me2a ChIP-Seq experiments were unsuccessful despite that we performed the experiment many times. This could be due to very low IP efficiency during ChIP-Seq analysis. Nevertheless, we found that ATAC accessibility sites were enriched in HCT116 cells at sites of increased ChIP density for SMARCA4, H3K4me3, and H3K27ac (Additional file 1: Fig. S5). These data suggest that *TNS4* and *EGFR* are direct downstream transcriptional targets of SMARCA4 in CRC cells.

### PRMT1 and SMARCA4 cooperatively activate *TNS4* and *EGFR* transcription

**Fig. 4:**
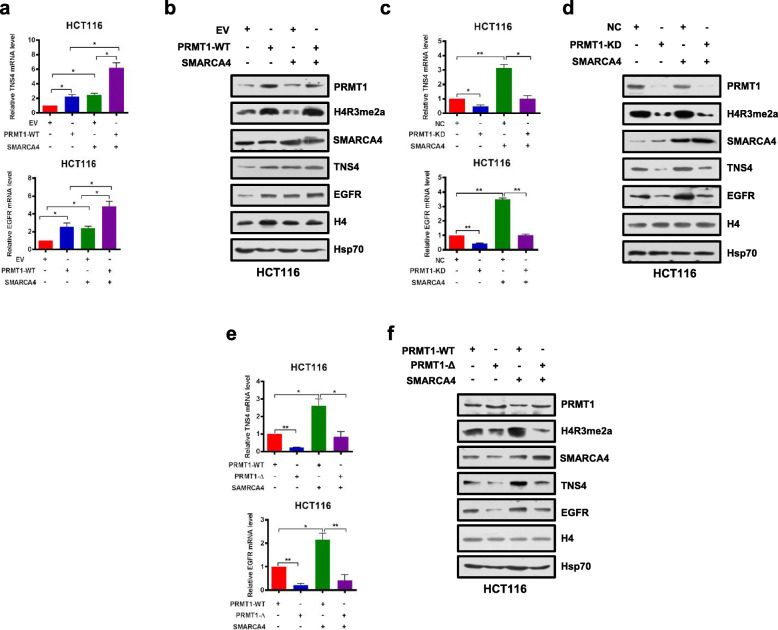
PRMT1 and SMARCA4 cooperatively activate *TNS4* and *EGFR* transcription in HCT116 cells. **a**, **b** Quantitative real-time PCR analysis of indicated mRNAs normalized to GAPDH (**a**) and western blot analysis of indicated proteins normalized to histone H4 and Hsp70 (**b**) from HCT116 cells that had been transfected with EV (empty vector, MSCV) or PRMT1-WT, and transfected or not with a SMARCA4 expression construct. **c**, **d** Quantitative real-time PCR analysis of indicated mRNAs normalized to GAPDH (**c**) and western blot analysis of indicated proteins normalized to histone H4 and Hsp70 (**d**) from NC or PRMT1-KD in HCT116 cells transfected or not with a SMARCA4 expression construct. **e**, **f** Quantitative real-time PCR analysis of indicated mRNAs normalized to GAPDH (**e**) and western blot analysis of indicated proteins normalized to histone H4 and Hsp70 (**f**) from HCT116 cells transfected with PRMT-WT or PRMT1-Δ constructs and transfected or not with a SMARCA4 expression construct. For **a**, **c**, and **e**, results are shown as mean ± s.d. from three independent experiments; *P < 0.05, **P < 0.01 compared with the indicated control

Given that knockdown of PRMT1 or SMARCA4 reduced mRNA and protein expression of TNS4 and EGFR in CRC cells (Fig. 3c, d), we sought to determine whether SMARCA4 and PRMT1 regulated *TNS4* and *EGFR* expression in a cooperative fashion. We enforced overexpression of PRMT1 or SMARCA4 or both in HCT116 and SW620 cells. We found that either PRMT1 or SMARCA4 individually activated expression of *TNS4* and *EGFR*, and the combination produced an additive effect on expression of *TNS4* and *EGFR*, whereas SMARCA4 knockdown did not have such an effect with PRMT1 (Fig. 4a, b, and Additional file 1: Fig. S6a, S6b, S6g, S6h). However, when PRMT1 was knocked down, this additive effect was lost (Fig. 4c, d, and Additional file 1: Fig. S6c, S6d, S6i, S6j). These results indicate that PRMT1 and SMARCA4 can cooperatively activate *TNS4* and *EGFR* transcription and that PRMT1 is required for this synergy. To further investigate whether enzymatic activity of PRMT1 is critical for the cooperative effect, we enforced overexpression of wild-type PRMT1 or of an enzymatic activity-associated deletion mutant, PRMT1-Δ, together with SMARCA4 in HCT116 and SW620 cells. We obtained results similar to those from PRMT1 knockdown, in that deletion mutant PRMT1-Δ did not show an additive effect on expression of *TNS4* and *EGFR* when co-expressed with SMARCA4 (Fig. 4e, f, and Additional file 1: Fig. S6e, S6f, S6k, S6l). These results indicate that methyltransferase activity of PRMT1 is essential for the cooperative effect of PRMT1 and SMARCA4 on transcriptional activation of *TNS4* and *EGFR* expression.

### SMARCA4 and PRMT1 combine to promote CRC cell proliferation through EGFR signaling

**Fig. 5:**
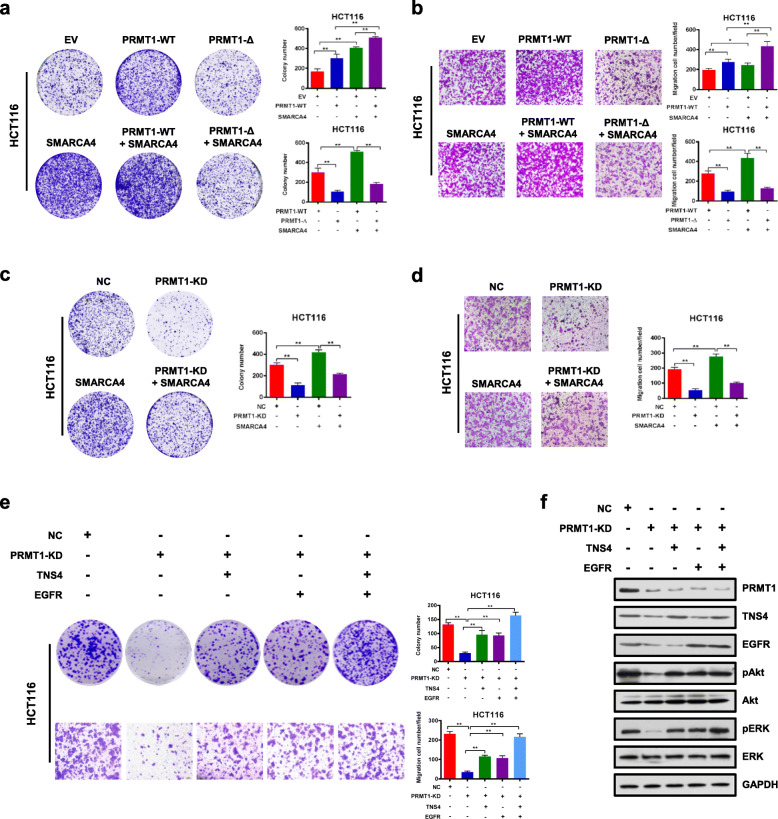
SMARCA4 couples with PRMT1 to promote CRC cell proliferation through EGFR signaling in HCT116 cells. **a** Colony formation assay with HCT116 cells transfected with EV (empty vector, MSCV), PRMT1-WT, PRMT1-Δ, SMARCA4, PRMT1-WT + SMARCA4, or PRMT1-Δ + SMARCA4. Representative images (left panels) and quantitative analyses of colony formation (right panels) are shown. **b** Cell migration assays with HCT116 cells transfected with MSCV, PRMT1-WT, PRMT1-Δ, SMARCA4, PRMT1-WT + SMARCA4, or PRMT1-Δ + SMARCA4. Representative images (left panels) and quantitative analyses of the migrated cells (right panels) are shown. **c** Colony formation assays from NC or PRMT1-KD transfected HCT116 cells transfected or not with a SMARCA4 expression construct. Representative images (left panels) and quantitative analyses of the colony formation (right panel) are shown. **d** Cell migration assays from NC or PRMT1-KD transfected HCT116 cells transfected or not with a SMARCA4 expression construct. Representative images (left panels) and quantitative analyses of the colony formation (right panel) are shown. **e** Colony formation assays and cell migration assays from NC or PRMT1-KD with ectopic expression of TNS4 or EGFR, or both. Representative images (left panels) and quantitative analyses of the colony formation (right panels) are shown. **f** Western blot analysis of the expression levels of PRMT1 and EGFR signaling pathway downstream molecules p-AKT, AKT, p-ERK, and ERK in HCT116 cells with ectopic expression of TNS4 or EGFR. GAPDH served as a loading control. All results are shown as mean ± s.d. from three independent experiments; **P < 0.01, *P < 0.05 compared with the indicated control

To determine the effect of SMARCA4 and PRMT1 on cell proliferation, we enforced overexpression of wild-type PRMT1 or SMARCA4 or both in HCT116 and SW620 cells, and determined colony formation and migration capabilities of those cells. We found that PRMT1 and SMARCA4 individually promoted colony formation and migration capabilities of both HCT116 and SW620 cells, and their co-expression resulted in an additive effect, whereas SMARCA4 knockdown did not have such an effect with PRMT1 (Fig. 5a, b, and Additional file 1: Fig. S7a, S7b, S7e, S7f). However, when PRMT1 was knocked down, this additive effect was lost (Fig. 5c, d, and Additional file 1: Fig. S7c, S7d, S7g, S7h). These results indicate that in combination PRMT1 and SMARCA4 cooperatively promote colony formation and migration capabilities of CRC cells. To further investigate whether enzymatic activity is critical for this cooperative effect, we enforced overexpression of wild-type PRMT1 or of the enzymatic activity-associated deletion mutant PRMT1-Δ together with SMARCA4 in HCT116 and SW620 cells. Colony formation and migration results were similar to the PRMT1 knockdown. The deletion mutant PRMT1-Δ did not mediate an additive effect on cell proliferation when co-expressed with SMARCA4 (Fig. 5a, b, and Additional file 1: Fig. S7a, S7b). These results indicate that the combination of SMARCA4 and PRMT1 promotes CRC cell proliferation, and this effect is dependent on methyltransferase activity of PRMT1, consistent with fact that SMARCA4 recognizes H4R3me2a, a PRMT1-mediated histone mark.

We next sought to determine whether PRMT1 promoted cell proliferation through activating EGFR signaling pathways, which include the Ras/MAPK pathway and the PI3K/AKT pathway [54]. We showed that overexpression of TNS4 and EGFR could restore the proliferation of PRMT1 knockdown cells (Fig. 5e). We found that knockdown of PRMT1 significantly decreased expression of TNS4 and EGFR genes. More interestingly, knockdown of PRMT1 also reduced phosphorylated Akt (p-Akt) and phosphorylated (p-ERK) but did not affect total protein levels. Importantly, the decrease in p-Akt and p-ERK could be rescued by enforced ectopic expression of either TNS4 or EGFR (Fig. 5f). Collectively, these data suggest that PRMT1 and SMARCA4 cooperatively promote CRC cell proliferation in a manner involving activating EGFR signaling and requiring PRMT1 methyltransferase activity.

### PRMT1 deficiency protects mice against DSS-induced Apc^min/+^ CRC progression

**Fig. 6:**
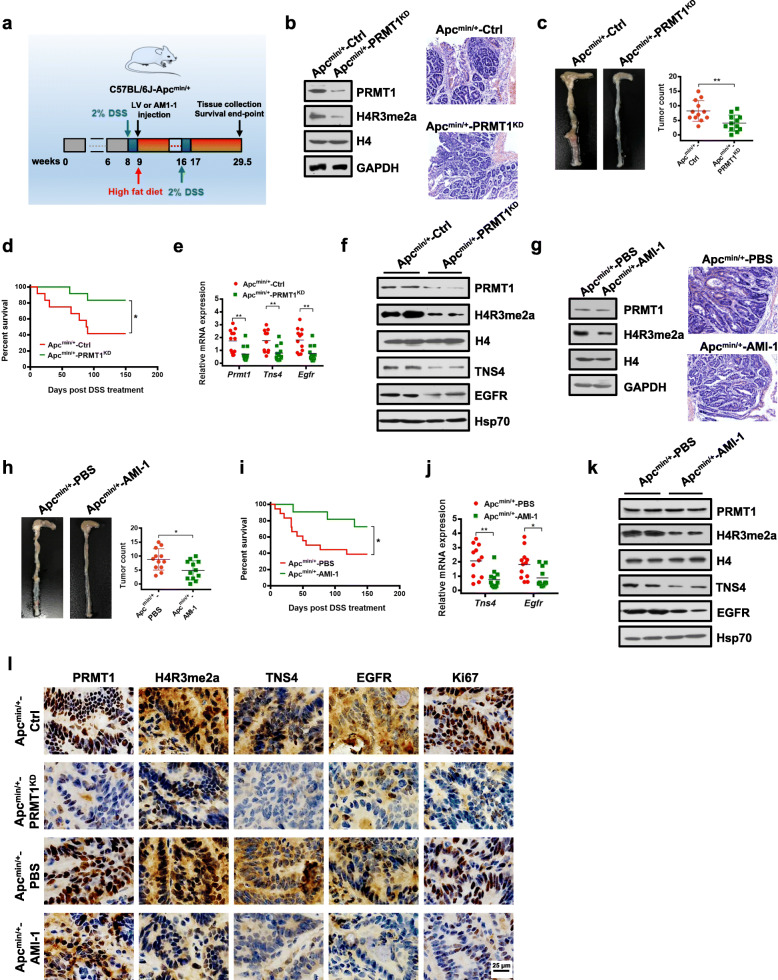
PRMT1 deficiency protects Apc^min/+^ mice against DSS-induced CRC progression. **a** Schematic diagram of DSS-induced CRC in C57BL/6 J-Apc^min/+^ mice with high-fat diet and related treatments. Tissue collection, analysis, and survival end-point analyses were performed at day 150 after the first DSS treatment. **b** Immunoblot analyses of PRMT1 and H4R3me2a in colon tissues (left) and hematoxylin and eosin (H&E) staining of colon tumors (right) from Apc^Min/+^-Ctrl and Apc^Min/+^-PRMT1^KD^ mice after the indicated LV infection. **c** Numbers and size of colon tumors found in Apc^Min/+^-PRMT1^KD^ mice (*n* = 12) compared with Apc^Min/+^-Ctrl mice (*n* = 12). Results are shown as mean ± s.d.; **P < 0.01 compared with the control mice. **d** Survival curves of Apc^Min/+^-Ctrl (*n* = 12) and Apc^Min/+^-PRMT1^KD^ (*n* = 12) mice. Statistical significance was determined by Kaplan–Meier log-rank test; *P < 0.05. **e**, **f** Quantitative real-time PCR analysis of indicated mRNAs normalized to GAPDH (**e**) and western blot analysis of indicated proteins normalized to histone H4 and Hsp70 (**f**) from colon tissues from Apc^Min/+^-Ctrl and Apc^Min/+^-PRMT1^KD^ mice. Results are shown as mean ± s.d. from 12 mice each; **P < 0.01 compared with the control mice. **g** Immunoblot analyses of PRMT1 and H4R3me2a in colon tissues (left) and hematoxylin and eosin (H&E) staining of colon tumors (right) from Apc^Min/+^-PBS and Apc^Min/+^-AMI-1 mice. **h** Numbers and size of colon tumors found in Apc^Min/+^-AMI-1 mice (*n* = 12) compared with Apc^Min/+^-PBS mice (*n* = 12). Results are shown as mean ± s.d.; *P < 0.05 compared with the control mice. **i** Survival curves of Apc^Min/+^-PBS (*n* = 12) and Apc^Min/+^-AMI-1 (*n* = 12) mice. Statistical significance was determined by the Kaplan–Meier log-rank test. *P < 0.05. **j**, **k** Quantitative real-time PCR analysis of indicated mRNAs normalized to GAPDH (**j**) and western blot analysis of indicated proteins normalized to histone H4 and Hsp70 (**k**) from colon tissues from Apc^Min/+^-PBS and Apc^Min/+^-AMI-1 mice. Results are shown as mean ± s.d. from 12 mice each; *P < 0.05, **P < 0.01 compared with the control mice. **l** Representative IHC staining of PRMT1, H4R3me2a, TNS4, EGFR, and Ki67 in colon tumor tissues of C57BL/6 J-Apc^Min/+^ mice from indicated groups

*APC*^*Min/+*^ mice provide a robust model for studying colon cancer progression. When raised conventionally, these mice develop small intestinal tumors, but if treated with 2% dextran sodium sulfate (DSS) in drinking water, they develop colitis and colonic neoplasia at very high penetrance [55]. Additional dietary modification with high fat accelerates carcinogenesis, providing a model that reflects the development of CRC in humans [56]. Using this model, we investigated whether PRMT1 participated in colon tumorigenesis in the *Apc*^*Min/+*^ mouse. First, we examined the role of PRMT1 in colon tumorigenesis by knocking down PRMT1 in C57BL/6 J-Apc^Min/+^ mice fed a high-fat diet (Fig. 6a). Decreased *PRMT1* expression was observed in the colons of mice after the indicated lentivirus virus (LV) infection (Fig. 6b). Reduced levels of histone mark H4R3me2a were confirmed in PRMT1 knockdown colon tissues (Fig. 6b). As demonstrated by histologic examination and hematoxylin and eosin (H&E) staining of mice colon tissues, we found that C57BL/6 J-Apc^Min/+^ mice pretreated with DSS followed by LV infection-mediated PRMT1 knockdown (Apc^Min/+^-PRMT1^KD^) developed significantly fewer and smaller visible tumors and microadenomas within their colons than did control scramble-treated mice (Apc^Min/+^-Ctrl) (Fig. 6b, c, l). In addition, the survival rate was significantly higher in Apc^Min/+^-PRMT1^KD^ mice than in Apc^Min/+^-Ctrl mice (Fig. 6d). As expected, colon tissues from Apc^Min/+^-PRMT1^KD^ mice had reduced expression of *TNS4* and *EGFR* genes (Fig. 6e, f).

To test the importance of PRMT1 methyltransferase activity for colon tumorigenesis in vivo, we utilized the PRMT1 inhibitor AMI-1 [53, 57] to inhibit its methyltransferase activity. We demonstrated that the AMI-1 treatment could mimick the phenotypes caused by the PRMT1-Δ on colony formation, migration capabilities, and expression of *TNS4* and *EGFR* of CRC cells in a dose-dependent manner (Additional file 1: Fig. S8a-d). Similar to Apc^Min/+^-PRMT1^KD^ CRC mice, AMI-1 treatment significantly reduced the formation of colorectal adenomas in C57BL/6J-Apc^Min/+^ mice (Apc^Min/+^-AMI-1) compared to the PBS-treated mice (Fig. 6a, h, l). In addition, the survival rate was significantly higher in AMI-1-treated mice than in PBS-treated mice (Fig. 6i). As expected, reduced levels of histone mark H4R3me2a were confirmed in AMI-1-treated colon tissues compared to PBS-treated colon tissues (Fig. 6g). Quantitative RT-PCR and Western blot analysis showed that colon tissues from AMI-1-treated mice had significantly lower expression levels of *TNS4* and *EGFR* genes compared to PBS-treated mice (Fig. 6j, k). Consistent with our observation of the in vivo treatment response, Ki67 staining showed that PRMT1 depletion or inhibition suppressed proliferation of colon tumor cells (Fig. 6l). More interestingly, combination treatment of APC^min/+^ mice using AMI-1 and cetuximab, a therapeutic EGFR monoclonal antibody [57], showed significant synergy to reduce the formation of colorectal adenomas in mice (Additional file 1: Fig. S9). Taken together, these data indicate that PRMT1 depletion or inhibition ameliorates CRC tumorigenesis in vivo.

### Upregulation of SMARCA4 positively correlates with expression of EGFR and TNS4 in CRC and is associated with poor prognosis of CRC patients

**Fig. 7:**
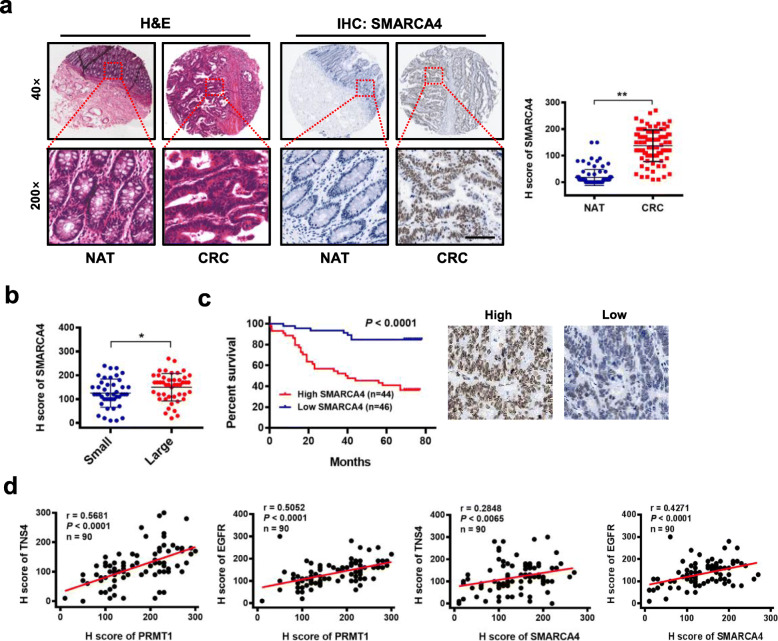
Upregulation of SMARCA4 positively correlates with expression of EGFR and TNS4 and is associated with poor prognosis of CRC patients. **a** Hematoxylin and eosin (H&E) staining and IHC staining of SMARCA4 protein in adjacent normal colon tissue controls (NAT) and colorectal cancer (CRC) in human tissues. Representative micrographs are shown in original magnification (× 200) as indicated (left); total IHC score of SMARCA4 in NAT and CRC tissues (*n* = 90); **P < 0.01 (right). Scale bar, 50 μm. **b** Correlation of SMARCA4 expression and tumor size in CRC patients; *P < 0.05. **c** Kaplan–Meier plot of overall survival of 90 patients with colorectal cancer, stratified by SMARCA4 expression; log-rank test, *P* < 0.0001. **d** Pearson correlation scatter plot of H scores of PRMT1 or SMARCA4, and TNS4 or EGFR in human colorectal cancer (*n* = 90)

To investigate the clinical significance of SMARCA4 expression in patients with CRC, we examined the protein expression profile of SMARCA4 in human CRC specimens and adjacent normal colon tissues. Consistent with previous observations from other groups [58–60], the immunohistochemical analysis showed significant upregulation of SMARCA4 expression in human CRC specimens compared to normal tissues (Fig. 7a). Interestingly, SMARCA4 expression correlated with tumor size (Fig. 7b), although differences in tumor grade, TNM stage, or lymph node metastasis were not significant (Additional file 2: Table S1). Kaplan–Meier survival analysis showed that CRC patients with high levels of SMARCA4 expression had shorter overall survival than other CRC patients (Fig. 7c). Notably, the expression of PRMT1 and TNS4, PRMT1 and EGFR, SMARCA4 and TNS4, and SMARCA4 and EGFR correlated well across all CRC samples analyzed (Fig. 7d). These results indicate that SMARCA4 expression levels are upregulated in human colorectal cancer tissues and correlate with poor prognosis in colorectal cancer, indicating that SMARCA4 may positively regulate TNS4 and EGFR expression to promote CRC proliferation during colorectal cancer progression.

## Discussion

**Fig. 8:**
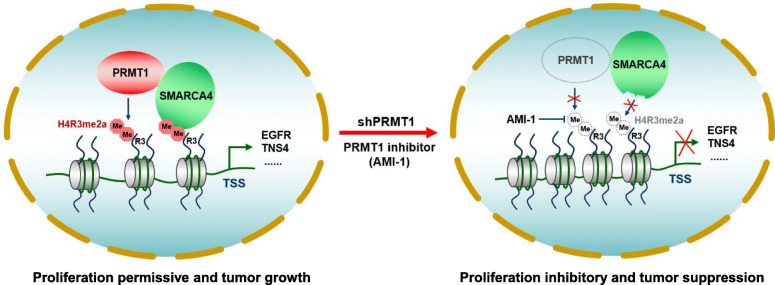
A hypothetical model of PRMT1 and SMARCA4 regulating cell proliferation and CRC progression

The methylation of arginine residues is catalyzed primarily by members of the protein arginine methyltransferase family, of which PRMT1 is the predominant member in mammalian cells [61]. Numerous studies have addressed the regulatory mechanisms and roles of PRMT1 in human cancers including breast, prostate, lung cancer, and leukemia, but little is known about the regulatory roles of PRMT1 in CRC progression [8]. In this study, we show that PRMT1-mediated histone modification H4R3me2a is critical for SMARCA4 recruitment to activate *TNS4* and *EGFR* expression and promote the proliferative, colony-formative, and migratory abilities of CRC cells (Fig. 8). In vivo, knockdown or inhibition of PRMT1 profoundly attenuated the growth of CRC cells in the C57BL/6 J-Apc^Min/+^ CRC mice model. Additionally, elevated expression of PRMT1 or SMARCA4 in CRC patients were positively correlated with expression of EGFR and TNS4, and CRC patients had shorter overall survival, which is consistent with observations by Mathioudaki and co-workers [14].

PRMT1 specifically deposits the H4R3me2a mark on histone H4, which is generally associated with transcriptional activation [61]. The link between H4R3me2a and activation of gene expression has also been established in the case of leukemia driven by an aberrant MLL1-EEN-Sam68-PRMT1 complex, where PRMT1 activity seems to cooperate with MLL1-driven H3K4 methylation to activate target genes [62]. Yang et al. reported that the Tudor domain-containing protein TDRD3 binds isolated methyl-marks H4R3me2a [9]. To the best of our knowledge, there is no other reported identifying effector molecules of H4R3me2a associated with transcriptional activation. One key observation in our study is that we provide compelling evidence that a portion of the ATPase domain of SMARCA4 (aa1009-1314), which contains an intact helicase superfamily c-terminal domain (HELICc), is an effector module that can bind the H4R3me2a mark and can discriminate between H4R3me2a and H4R3me2s marks. Consistent with our finding, Sen et al. showed that the ATPase domain of the SWI/SNF chromatin remodeling complex was necessary for interacting with the histone portion of the nucleosome in yeast [63, 64]. Strikingly, they determined that the yeast SnAC domain (aa 1312-1444) is a crucial histone anchor for SWI/SNF complex to mobilize nucleosomes. However, our current study did not show an interaction of the H4R3me2a peptide in vitro with SMARCA4-F5 (aa1315-1647) which contains the SnAC domain of SMARCA4. One possible explanation for this discrepancy is that the mammalian SnAC domain does not contribute to the affinity of the SWI/SNF complex for nucleosomes, but may rather be pivotal for regulating the activity of the ATPase domain [63]. Apart from that, it would be really interesting to explore the possibility whether SMARCA2, which harbors the highly conserved HELICc domain as well [65], could also bind H4R3me2a mark. The structural insights in the future would provide direct evidence for the selective interaction between SMARCA4 and the H4R3me2a mark.

Nonetheless, we provide evidence to show that SMARCA4 promotes colorectal cancer cell proliferation by interacting with PRMT1 to activate *TNS4* and *EGFR* transcription. We determined that one mechanism by which SMARCA4 promotes CRC progression is via the epigenetic regulation of *TNS4* and *EGFR*. SMARCA4 is recruited to the TNS4 and EGFR promoter through binding PRMT1-mediated H4R3me2a, leading to enhanced proliferation and migration of CRC cells, as well as formation of colorectal tumors in mice. Although its role in cancer progression remains a constant topic of debate, interest in elucidating the roles of SMARCA4 in cancer progression is at an all-time high. Interestingly, PRMT5 has been shown required for SMARCA4-dependent chromatin remodeling to facilitate myogenesis [66]. Some evidence of an oncogenic role for SMARCA4 was provided by Li and co-workers [67]. They reported that SMARCA4 interacted with Sp1 to activate LTBP2 transcription, thus promoting lung cancer progression [67]. However, Futreal and colleagues have shown that SMARCA4 acts as a tumor suppressor by directing a transcriptional program that steers lung cancer cells away from drawing energy from oxidative phosphorylation [68]. SMARCA4 inactivation is known to be associated with increased non-small cell lung cancer aggressiveness, which suggests that SMARCA4 functions as a tumor suppressor [69]. Romero et al. found that SMARCA4 antagonizes Myc activity and promotes cell differentiation in lung cancer [70]. Nevertheless, in the murine small intestine, SMARCA4 loss in an Apc-deficient context attenuated aberrant Wnt signaling and prevented Wnt-driven tumor initiations [30]. A recent report by Liu and colleagues showed that SMARCA4 is required for the homeostatic maintenance of intestinal epithelial cells to impede inflammation-associated CRC tumorigenesis [32]. Thus, unsurprisingly, it appears plausible that SMARCA4 can act as either an oncogene or tumor suppressor in a context- and cell type-specific manner. It is reasonable to extrapolate our finding to a larger scenario wherein SMARCA4, through coordinating the actions of multiple transcription factors or epigenetic regulators, modulates a transcriptional program that promotes cancer progression. It will be of interest to test this hypothesis.

Previous studies have demonstrated that SMARCA4 acts as a positive regulator of Wnt signaling in the small intestinal epithelium and plays a pivotal role in development and homeostasis of the duodenum through regulation of Notch signaling [30, 31]. In the current study, we also sought to explore whether Wnt or Notch signaling was altered in CRC cells in which SMARCA4 was depleted. Consistent with previous studies, gene ontology (GO) analysis showed that the Wnt signaling pathway was altered in CRC cells following SMARCA4 loss, whereas no noticeable alternations in the Notch signaling pathway were observed (Additional file 1: Fig. S4c). Moreover, both Wnt and Notch signaling pathways are significantly altered in PRMT1-depleted cells (Additional file 1: Fig. S4c). Notably, we observed that EGFR signaling correlated the best with PRMT1 in CRC cells. Indeed, in CRC, Liao et al. [57] and others showed that PRMT1-mediated EGFR methylation increases tumorigenesis in an orthotopic CRC mouse model and mediates resistance to cetuximab treatment in triple-negative breast cancer cells and head and neck cancer, suggesting that PRMT1 is highly related to EGFR functionality [10, 57, 71–73]. These findings are in agreement with studies reporting a tumor-promoting role of PRMT1 in acquisition of malignant characteristics associated with colon cancer progression. Thus, our data support the notion that PRMT1 may be a key regulator driving cancer progression through regulating multiple signaling pathways in CRC. However, given that SMARCA4 is a key epigenetic regulator involved in multiple transcriptional processes, we cannot fully exclude the possibility that other signaling changes also contribute to the phenotype observed in the present study.

In summary, our findings link PRMT1-mediated H4R3me2a and SMARCA4 in promoting CRC progression via enhancement of EGFR signaling. Future studies exploiting epigenomic tools and animal models will be of interest to fully elucidate the connection between SMARCA4-dependent transcription and CRC progression.

## Conclusion

Our present study reveals that PRMT1 which mainly catalyzes asymmetric dimethylation of histone H4 on arginine 3 (H4R3me2a), and SMARCA4, act cooperatively to promote CRC progression by enhancing EGFR signaling. These findings demonstrate a critical interplay between transcriptional and epigenetic control during CRC progression, suggesting that SMARCA4 is a novel key epigenetic modulator of CRC. Our findings thus highlight PRMT1/SMARCA4 inhibition as a potential effective therapeutic intervention strategy for CRC.

## Supplementary Information

**Additional file 1.** Supplementary figures and related figure legends. **Fig. S1.** SMARCA4 binds specifically to histone H4R3me2a mark. **Fig. S2.** ITC assay to identify direct interactions between SMARCA4-F4 and H4, H4R3me2a, or H4R3me2s peptides. **Fig. S3.** Interation of SMARCA4 and PRMT1. **Fig. S4.** Identification of transcriptional targets for PRMT1 and SMARCA4 in HCT116 cells. **Fig. S5.** Characterization of ATAC-seq, along with ChIP-Seq of SMARCA4, H3K4me1, H3K4me3, H3K27ac in HCT116 cells. **Fig. S6.** PRMT1 and SMARCA4 cooperatively activate TNS4 and EGFR transcription in SW620 and HCT116 cells. **Fig. S7.** SMARCA4 couples with PRMT1 to promote CRC cell proliferation in SW620 and HCT116 cells. **Fig. S8.** AMI-1, a PRMT1 inhibitor, blocks HCT116 cell proliferation, and inhibits TNS4 and EGFR expression. **Fig. S9.** Combined treatment with AMI-1 and Cetuximab synergistically protects Apc^min/+^ mice against DSS-induced CRC progression.

**Additional file 2: Supplementary tables. Table S1.** Clinicopathologic characteristics of PRMT1 and SMARCA4 expression in CRC patients. **Table S2.** 15 common target genes listed both in ChIP-Seq and microarray data. **Table S3.** A list of antibodies used in the present study. **Table S4.** Peptides used in the present study. **Table S5.** The list of primer sequences for RT-PCR. **Table S6.** The list of primer sequences for ChIP. **Table S7.** The list of viruses used in the present study. Table S8: Tissue array information. **Table S9.** List of proteins identified by Mass Spectrometry. **Table S10.** 109 common genes down-regualted by PRMT1-knockdown and SMARCA4-knockdown in HCT116 cells.

**Additional file 3.** Scans of the uncropped blots for Western blots. Fig. S1. Uncropped blots for Western blots in Fig. 1. Fig. S2. Uncropped blots for Western blots in Fig. 2. Fig. S3. Uncropped blots for Western blots in Fig. 3. Fig. S4. Uncropped blots for Western blots in Fig. 4. Fig. S5. Uncropped blots for Western blots in Fig. 5. Fig. S6. Uncropped blots for Western blots in Fig. 6. Fig. S7. Uncropped blots for Western blots in Additional file 1: Fig. S1. Fig. S8. Uncropped blots for Western blots in Additional file 1: Fig. S3. Fig. S9. Uncropped blots for Western blots in Additional file 1: Fig. S6. Fig. S10. Uncropped blots for Western blots in Additional file 1: Fig. S8.

## Data Availability

Our gene-microarray sequencing data have been deposited in Gene Expression Omnibus (GEO) with accession numbers GSE143198 and GSE143199 (https://www.ncbi.nlm.nih.gov/geo/query/acc.cgi?acc=GSE143198) [74] and (https://www.ncbi.nlm.nih.gov/geo/query/acc.cgi?acc=GSE143199) [75]. Mass spectrometric data have been deposited in PeptideAtlas with accession number PASS01646 (http://www.peptideatlas.org/PASS/PASS01646) [76].

## Abbreviations

PRMT1: Protein arginine methyl transferase 1
H4R3me2a: Asymmetric dimethylation of histone H4 on arginine 3
CRC: Colorectal cancer
SMARCA4: SWI/SNF related matrix-associated actin-dependent regulator of chromatin subfamily A member 4
Brg1: Brahma-related gene 1
ITC: Isothermal titration calorimetry
MST: Microscale thermophoresis
IHC: Immunohistochemical staining
NAT: Matched adjacent normal colon tissues
GSEA: Gene set enrichment analysis
GO: Gene ontology
TNS4: Tensin 4
EGFR: Epidermal growth factor receptor
DSS: Dextran sodium sulfate
LV: Lentivirus virus
H&E: Hematoxylin and eosin staining
AMI-1: Arginine methyltransferase inhibitor 1Apc: Adenomatous polyposis coli
EdU: 5-Ethynyl-20-deoxyuridine
ChIP: Chromatin immunoprecipitation
ATAC: Assay for transposase-accessible chromatin
