# Correction to: PRMT1-mediated H4R3me2a recruits SMARCA4 to promote colorectal cancer progression by enhancing EGFR signaling

**DOI:** 10.1186/s13073-021-00966-z

**Published:** 2021-10-04

**Authors:** Bing Yao, Tao Gui, Xiangwei Zeng, Yexuan Deng, Zhi Wang, Ying Wang, Dongjun Yang, Qixiang Li, Peipei Xu, Ruifeng Hu, Xinyu Li, Bing Chen, Jin Wang, Ke Zen, Haitao Li, Melissa J. Davis, Marco J. Herold, Hua-Feng Pan, Zhi-Wei Jiang, David C. S. Huang, Ming Liu, Junyi Ju, Quan Zhao

**Affiliations:** 1 The State Key Laboratory of Pharmaceutical Biotechnology, Department of Hematology, the Affiliated Drum Tower Hospital of Nanjing University Medical School, China-Australia Institute of Translational Medicine, School of Life Sciences, Nanjing University, 163 Xianlin Avenue, Nanjing, 210023 China; 2 Department of Medical Genetics, Nanjing Medical University, Nanjing, China; 3 Beijing Advanced Innovation Center for Structural Biology, Beijing Frontier Research Center for Biological Structure, Tsinghua-Peking Joint Center for Life Sciences, School of Medicine, Tsinghua University, Beijing, China; 4 The Walter and Eliza Hall Institute of Medical Research, Department of Medical Biology, University of Melbourne, Melbourne, VIC Australia; 5 Department of General Surgery, the Affiliated Hospital of Nanjing University of Chinese Medicine, Nanjing, China

**Correction to: Genome Med 13, 58 (2021)**

**DOI: 10.1186/s13073-021-00871-5**

In this article [1] the incorrect figure was shown as Fig. 5 and the incorrect figure appeared in Additional File 1 as Fig. S7. The figures should have appeared as shown below. The original article has been updated.

**Fig. 5:**
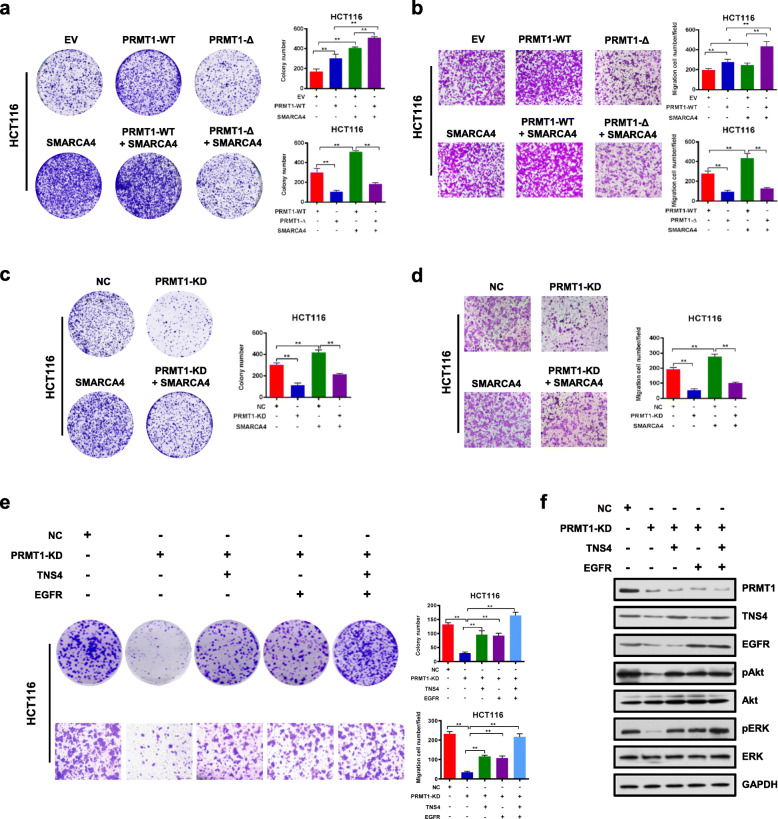
SMARCA4 couples with PRMT1 to promote CRC cell proliferation through EGFR signaling in HCT116 cells. **a** Colony formation assay with HCT116 cells transfected with EV (empty vector, MSCV), PRMT1-WT, PRMT1-**Δ**, SMARCA4, PRMT1-WT + SMARCA4, or PRMT1-**Δ** + SMARCA4. Representative images (left panels) and quantitative analyses of colony formation (right panels) are shown. **b** Cell migration assays with HCT116 cells transfected with MSCV, PRMT1-WT, PRMT1-**Δ**, SMARCA4, PRMT1-WT + SMARCA4, or PRMT1-Δ + SMARCA4. Representative images (left panels) and quantitative analyses of the migrated cells (right panels) are shown. **c** Colony formation assays from NC or PRMT1-KD transfected HCT116 cells transfected or not with a SMARCA4 expression construct. Representative images (left panels) and quantitative analyses of the colony formation (right panel) are shown. **d** Cell migration assays from NC or PRMT1-KD transfected HCT116 cells transfected or not with a SMARCA4 expression construct. Representative images (left panels) and quantitative analyses of the colony formation (right panel) are shown. **e** Colony formation assays and cell migration assays from NC or PRMT1-KD with ectopic expression of TNS4 or EGFR, or both. Representative images (left panels) and quantitative analyses of the colony formation (right panels) are shown. **f** Western blot analysis of the expression levels of PRMT1 and EGFR signaling pathway downstream molecules p-AKT, AKT, p-ERK, and ERK in HCT116 cells with ectopic expression of TNS4 or EGFR. GAPDH served as a loading control. All results are shown as mean ± s.d. from three independent experiments; **P < 0.01, *P < 0.05 compared with the indicated control

## Supplementary Information

**Additional file 1.** Supplementary figures and related figure legends. Fig. S1. SMARCA4 binds specifically to histone H4R3me2a mark. Fig. S2. ITC assay to identify direct interactions between SMARCA4-F4 and H4, H4R3me2a, or H4R3me2s peptides. Fig. S3. Interation of SMARCA4 and PRMT1. Fig. S4. Identification of transcriptional targets for PRMT1 and SMARCA4 in HCT116 cells. Fig. S5. Characterization of ATAC-seq, along with ChIP-Seq of SMARCA4, H3K4me1, H3K4me3, H3K27ac in HCT116 cells. Fig. S6. PRMT1 and SMARCA4 cooperatively activate TNS4 and EGFR transcription in SW620 and HCT116 cells. Fig. S7. SMARCA4 couples with PRMT1 to promote CRC cell proliferation in SW620 and HCT116 cells. Fig. S8. AMI-1, a PRMT1 inhibitor, blocks HCT116 cell proliferation, and inhibits TNS4 and EGFR expression. Fig. S9. Combined treatment with AMI-1 and Cetuximab synergistically protects Apcmin/+ mice against DSS-induced CRC progression.
