# Supplementary material for: PRMT1-mediated H4R3me2a recruits SMARCA4 to promote colorectal cancer progression by enhancing EGFR signaling
Source: Genome Med. 2021 Apr 14;13:58. doi: 10.1186/s13073-021-00871-5 (PMC8048298; doi:10.1186/s13073-021-00871-5)
Supplement: Supplementary file 1 — Additional file 1. Supplementary figures and related figure legends. Fig. S1. SMARCA4 binds specifically to histone H4R3me2a mark. Fig. S2. ITC assay to identify direct interactions between SMARCA4-F4 and H4, H4R3me2a, or H4R3me2s peptides. Fig. S3. Interation of SMARCA4 and PRMT1. Fig. S4. Identification of transcriptional targets for PRMT1 and SMARCA4 in HCT116 cells. Fig. S5. Characterization of ATAC-seq, along with ChIP-Seq of SMARCA4, H3K4me1, H3K4me3, H3K27ac in HCT116 cells. Fig. S6. PRMT1 and SMARCA4 cooperatively activate TNS4 and EGFR transcription in SW620 and HCT116 cells. Fig. S7. SMARCA4 couples with PRMT1 to promote CRC cell proliferation in SW620 and HCT116 cells. Fig. S8. AMI-1, a PRMT1 inhibitor, blocks HCT116 cell proliferation, and inhibits TNS4 and EGFR expression. Fig. S9. Combined treatment with AMI-1 and Cetuximab synergistically protects Apcmin/+ mice against DSS-induced CRC progression. [file 13073_2021_871_MOESM1_ESM.doc]

**Additional file 1**

**Fig. S1.** SMARCA4 binds specifically to histone H4R3me2a mark. **a** SDS-PAGE analysis of purified GST-fused recombinant SMARCA4-F1 (aa1-351), SMARCA4-F2 (aa352-707), SMARCA4-F3 (aa705-1008), SMARCA4-F4 (aa1009-1314) and SMARCA4-F5 (aa1315-1647) from *E. Coli* stained by Coomassie brilliant blue. MW: protein molecular weight markers. **b** A schematic diagram shows the essential domains required for the interaction with H4R3me2a peptide of SMARCA4. **c** SDS-PAGE analysis of purified full-length Flag-tagged SMARCA4 (Flag-SMARCA4, aa1-1647) from HCT116 cells. **d** Peptide pull-down assay to detect the interactions between H4, H4R3me2a, and H4R3me2s peptides and purified Flag-SMARCA4 from HCT116 cells (top panel). Coomassie staining shows equivalent loading of the three peptides (bottom panel). **e** MST assay to identify direct interactions between purified Flag-SMARCA4 from HCT116 cells and H4R3me2a peptides. The dissociation constant (Kd) between SMARCA4 and H4R3me2a or H4R3me2s peptides is 6.27±0.37 μM or 661 ± 73.1 μM respectively.

**Fig. S2.** ITC assay to identify direct interactions between SMARCA4-F4 and H4, H4R3me2a, or H4R3me2s peptides.

**Fig. S3.** Interation of SMARCA4 and PRMT1. **a** Cellular localization of PRMT1 and SMARCA4 in HCT116 cells shown by immunofluorescence with anti-PRMT1, anti-SMARCA4, and DAPI nuclear counterstaining. Scale bar, 10 μM. **b** Total proteins from HCT116 cells transfected with Flag-PRMT1 were extracted and immunoprecipitated with antibodies against Flag followed by immunoblotting with antibodies against SMARCA4 or PRMT1. **c** Co-immunoprecipitation of endogenous SMARCA4 and PRMT1 from HCT116 cells. **d** ChIP-reChIP analysis of chromatin from HCT116 cells. The first antibody (PRMT1) and second antibody (SMARCA4) used in the re-ChIP are shown below the bar plot. The amount recovered from the ChIP (first ChIP) and re-ChIP (second ChIP) was determined by qPCR, and is shown as a percentage of the input. All results are shown as mean±s.d. from three independent experiments; **P*<0.05, ***P*<0.01 compared with the indicated control.

**Fig. S4.** Identification of transcriptional targets for PRMT1 and SMARCA4 in HCT116 cells. **a** Heat map showing differentially expressed mRNA levels in PRMT1 knockdown (PRMT1-KD1 and PRMT1-KD2) or NC cells (NC-1 and NC-2, fold change value >1.5 and *P* < 0.01). **b** Heat map showing differentially expressed mRNA levels in SMARCA4 knockdown (SMARCA4-KD1 and SMARCA4-KD2) or NC cells (NC-1 and NC-2, fold change value >1.5 and *P* < 0.01). **c** Gene ontology (GO) analysis of the target genes of PRMT1 or SMARCA4 arranged into functional groups. **d** Venn diagram illustrating the overlap of target genes identified by microarray analysis as being regulated by PRMT1 and SMARCA4 in HCT116 cells. **e** Venn diagram illustrating the overlap between target genes regulated by PRMT1/SMARCA4 identified by microarray analysis and SMARCA4 target genes identified by ChIP-seq in HCT116 cells.

**Fig. S5**. Characterization of ATAC-seq, along with ChIP-seq of SMARCA4, H3K4me1, H3K4me3, H3K27ac in HCT116 cells. TSS, transcription start site.

**Fig. S6.** PRMT1 and SMARCA4 cooperatively activate TNS4 and EGFR transcription in SW620 and HCT116 cells. **a, b** Quantitative real-time PCR analysis of indicated mRNAs normalized to GAPDH (**a**) and western blot analysis of indicated proteins normalized to histone H4 and Hsp70 (**b**) from SW620 cells that had been transfected with MSCV (empty vector) or PRMT1-WT, and transfected or not with a SMARCA4 expression construct. **c, d** Quantitative real-time PCR analysis of indicated mRNAs normalized to GAPDH (**c**) and western blot analysis of indicated proteins normalized to histone H4 and Hsp70 (**d**) from NC or PRMT1-KD in SW620 cells transfected or not with a SMARCA4 expression construct. **e, f** Quantitative real-time PCR analysis of indicated mRNAs normalized to GAPDH (**e**) and western blot analysis of indicated proteins normalized to histone H4 and Hsp70 (**f**) from SW620 cells transfected with PRMT-WT or PRMT1-Δ constructs and transfected or not with a SMARCA4 expression construct. **g, h** Quantitative real-time PCR analysis of indicated mRNAs normalized to GAPDH (**g**) and western blot analysis of indicated proteins normalized to histone H4 and Hsp70 (**h**) from HCT116 cells that had been transfected with MSCV (empty vector) or PRMT1-WT, and transfected or not with a SMARCA4-KD construct. **i, j** Quantitative real-time PCR analysis of indicated mRNAs normalized to GAPDH (**i**) and western blot analysis of indicated proteins normalized to histone H4 and Hsp70 (**j**) from NC or PRMT1-KD in HCT116 cells transfected or not with a SMARCA4-KD construct. **k, l** Quantitative real-time PCR analysis of indicated mRNAs normalized to GAPDH (**k**) and western blot analysis of indicated proteins normalized to histone H4 and Hsp70 (**l**) from HCT116 cells transfected with PRMT-WT or PRMT1-Δ constructs and transfected or not with a SMARCA4-KD construct. All results are shown as mean±s.d. from three independent experiments; **P*<0.05, ***P*<0.01 compared with the indicated control.

**Fig. S7.** SMARCA4 couples with PRMT1 to promote CRC cell proliferation in SW620 and HCT116 cells. **a** Colony formation assay with SW620 cells transfected with MSCV, PRMT1-WT, PRMT1-Δ, SMARCA4, PRMT1-WT+SMARCA4, or PRMT1-Δ+SMARCA4. Representative images (left panels) and quantitative analyses of colony formation (right panels) are shown. **b** Cell migration assays with SW620 cells transfected with MSCV, PRMT1-WT, PRMT1-Δ, SMARCA4, PRMT1-WT+SMARCA4, or PRMT1-Δ+SMARCA4. Representative images (left panels) and quantitative analyses of the migrated cells (right panels) are shown. **c** Colony formation assays from NC or PRMT1-KD transfected SW620 cells transfected or not with a SMARCA4 expression construct. Representative images (left panels) and quantitative analyses of the colony formation (right panel) are shown. **d** Cell migration assays from NC or PRMT1-KD transfected SW620 cells transfected or not with a SMARCA4 expression construct. Representative images (left panels) and quantitative analyses of the colony formation (right panel) are shown. **e** Colony formation assay with HCT116 cells transfected with MSCV, PRMT1-WT, PRMT1-Δ, SMARCA4-KD, PRMT1-WT+SMARCA4-KD, or PRMT1-Δ+SMARCA4-KD. Representative images (left panels) and quantitative analyses of colony formation (right panels) are shown. **f** Cell migration assays with HCT116 cells transfected with MSCV, PRMT1-WT, PRMT1-Δ, SMARCA4-KD, PRMT1-WT+SMARCA4-KD, or PRMT1-Δ+SMARCA4-KD. Representative images (left panels) and quantitative analyses of the migrated cells (right panels) are shown. **g** Colony formation assays from NC or PRMT1-KD transfected HCT116 cells transfected or not with a SMARCA4-KD construct. Representative images (left panels) and quantitative analyses of the colony formation (right panel) are shown. **h** Cell migration assays from NC or PRMT1-KD transfected HCT116 cells transfected or not with a SMARCA4-KD construct. Representative images (left panels) and quantitative analyses of the colony formation (right panel) are shown. All results are shown as mean±s.d. from three independent experiments; **P*<0.05, ***P*< 0.01 compared with the indicated control.

**Fig. S8.** AMI-1, a PRMT1 inhibitor, blocks HCT116 cell proliferation, and inhibits TNS4 and EGFR expression. **a** Colony formation assay with HCT116 cells treated with DMSO or AMI-1. Representative images (left panels) and quantitative analyses of colony formation (right panels) are shown. **b** Cell migration assays with HCT116 cells treated with DMSO or AMI-1. Representative images (left panels) and quantitative analyses of the migrated cells (right panels) are shown. **c, d** Quantitative real-time PCR analysis of indicated mRNAs normalized to GAPDH (**c**) and western blot analysis of indicated proteins normalized to histone H4 and Hsp70 (**d**) from HCT116 cells treated with DMSO or AMI-1. **e** ChIP analysis of SMARCA4 binding to the TNS4 and EGFR promoter in HCT116 cells treated with AMI-1. All results are shown as mean±s.d. from three independent experiments; **P*<0.05, ***P*< 0.01 compared with the indicated control.

**Fig. S9.** Combined treatment with AMI-1 and Cetuximab synergistically protects Apc^min/+^ mice against DSS-induced CRC progression. **a** Schematic diagram of DSS-induced CRC in C57BL/6J-Apc^min/+^ mice with high fat diet and related treatments with AMI-1, Cetuximab, or AMI-1 + Cetuximab. Tissue collection and analysis were performed at day 150 after the first DSS treatment. **b** Numbers and size of colon tumors found in Apc^Min/+^-AMI-1 mice (n=12), Apc^Min/+^-Cetuximab mice (n=12) or Apc^Min/+^-AMI-1 + Cetuximab (n=12) compared with Apc^Min/+^-PBS mice (n=12). Results are shown as mean±s.d. **P*<0.05, ***P*<0.01 compared with the indicated control.
