# Supplementary material for: PRMT1-mediated H4R3me2a recruits SMARCA4 to promote colorectal cancer progression by enhancing EGFR signaling
Source: Genome Med. 2021 Apr 14;13:58. doi: 10.1186/s13073-021-00871-5 (PMC8048298; doi:10.1186/s13073-021-00871-5)
Supplement: Supplementary file 2 — Additional file 2: Supplementary tables. Table S1. Clinicopathologic characteristics of PRMT1 and SMARCA4 expression in CRC patients. Table S2. 15 common target genes listed both in ChIP-Seq and microarray data. Table S3. A list of antibodies used in the present study. Table S4. Peptides used in the present study. Table S5. The list of primer sequences for RT-PCR. Table S6. The list of primer sequences for ChIP. Table S7. The list of viruses used in the present study. Table S8: Tissue array information. Table S9. List of proteins identified by Mass Spectrometry. Table S10. 109 common genes down-regualted by PRMT1-knockdown and SMARCA4-knockdown in HCT116 cells. [file 13073_2021_871_MOESM2_ESM.doc]

**Additional file 2**

**Table S1: Clinicopathologic characteristics of PRMT1 and SMARCA4 expression in CRC patients.**

| **Characteristics** | **Cases** | **IHC score of PRMT1** | |  | **IHC score of SMARCA4** | |
| --- | --- | --- | --- | --- | --- | --- |
|  |  | **Mean±s.d.** | ***P* value^a^** |  | **Mean±s.d.** | ***P* value^a^** |
| Gender  *Male*  *Female* | 47  43 | 162.1±74.4  178.1±58.7 | 0.2683 |  | 135.5 ± 65.8  139.5 ± 50.6 | 0.7513 |
| Age  ＞60  ≤60 | 58  32 | 156.6 ± 8.0  162.2 ± 12.0 | 0.6870 |  | 131.4 ± 5.9  129.4 ± 11.6 | 0.8650 |
| Tumor volume  *Large*  *Small* | 43  46 | 189.8 ± 10.2  148.7 ± 9.3 | 0.0036 |  | 150.7 ± 9.0  124.3 ± 8.2 | 0.0328 |
| Lymph node status^b^  *N_0_*  *N_1-3_* | 61  29 | 162.3 ± 8.3  185.5 ± 13.8 | 0.1319 |  | 138.5 ± 7.4  135.2 ± 11.8 | 0.8039 |
| Grade  *I-II*  *III-VI* | 14  76 | 207.1 ± 19.5  162.9 ± 7.5 | 0.0248 |  | 145.0 ± 20.9  136.1 ± 6.4 | 0.6071 |
| TNM stage  *T1-T2*  *T3* | 62  28 | 165.8 ± 9.3  195.4 ± 12.7 | 0.0730 |  | 141.8 ± 9.0  150.0 ± 12.4 | 0.6033 |

^a^ *P* values were derived using Student’s *t*-test to compare values for the two parameters in each category.

^b^ The tumor stage, lymph node status, and metastasis were classified according to the international system for staging CRC cancer [1].

**Reference:**

1. Weiser MR. AJCC 8th Edition: Colorectal Cancer. Ann Surg Oncol. 2018;25: 1454-5.

**Table S2: 15 common target genes listed both in ChIP-seq and microarray data**.

| **Gene Name** | **Fold change from RNA-seq data** | | | |  | **Regulatory potential score of SMARCA4^c^** |
| --- | --- | --- | --- | --- | --- | --- |
|  | **SMARCA4**  **-KD1** | **SMARCA4**  **-KD2** | **PRMT1**  **-KD1** | **PRMT1**  **-KD2** |  |  |
| TNS4 | 3.559 | 3.675 | 2.103 | 1.886 |  | 0.731 |
| SERPINA1 | 3.515 | 3.080 | 1.849 | 1.749 |  | 0.429 |
| ABCC3 | 2.923 | 2.538 | 1.954 | 2.041 |  | 1.096 |
| KIAA0040 | 2.368 | 1.746 | 1.880 | 1.684 |  | 0.268 |
| ICMT | 2.184 | 1.664 | 2.024 | 2.181 |  | 0.326 |
| UNC5A | 1.896 | 1.729 | 1.671 | 2.484 |  | 0.376 |
| HPSE | 1.800 | 1.629 | 1.571 | 1.572 |  | 0.276 |
| ANKRD2 | 1.799 | 1.534 | 1.837 | 1.523 |  | 1.250 |
| LOC100507002 | 1.757 | 1.533 | 1.534 | 1.503 |  | 0.607 |
| MBNL2 | 1.728 | 1.527 | 1.728 | 1.632 |  | 0.252 |
| EGFR | 1.677 | 1.851 | 1.520 | 1.660 |  | 0.284 |
| POC1A | 1.650 | 1.633 | 1.825 | 2.027 |  | 0.613 |
| SPC24 | 1.610 | 1.975 | 1.659 | 1.628 |  | 0.414 |
| TSPAN14 | 1.566 | 1.543 | 1.855 | 1.822 |  | 0.802 |
| SYNE2 | 1.527 | 1.535 | 1.948 | 1.855 |  | 0.412 |

**^c^** SMARCA4 ChIP-seq data was downloaded from the Gene Expression Omnibus database (GEO; GSM1835989) and the regulatory potential score of SMARCA4 was calculated using the Binding and Expression Target Analysis (BETA) in the Cistrome Data Browser (CDB; 71202).

**Table S3:** Antibodies

| **Antibodies** | **Source** | **Identifier** |
| --- | --- | --- |
| Anti-BRG1  Anti-H4R3me2a  Anti-H4R3me2s  Anti-H4  Anti-GST  Anti-PRMT1  Anti-PRMT1  Anti-Flag  Anti-Hsp70  Anti-EGFR  Anti-TNS4  Anti-Akt  Anti-p-Akt  Anti-ERK  Anti-p-ERK  Anti-GAPDH  Anti-mouse IgG  Anti-rabbit IgG | Abcam  Active Motif  Abcam  Abcam  Abcam  CST  Abcam  CST  Genscript  Abcam  Abcam  Abcam  Abcam  Abcam  Abcam  Abcam  Life Technologies  Life Technologies | ab110641  39705  ab5823  ab109463  ab19256  #2449  ab12198  #14793  A01236  ab52894  ab192247  ab179463  ab81283  ab32537  ab65142  ab181602  A32723  A32740 |

**Table S4:** Peptides

| **Peptides, chemicals** | **Source** | **Identifier** |
| --- | --- | --- |
| H4  H4R3me2a  H4R3me2s | Genscript, Nanjing, China  Genscript, Nanjing, China  Genscript, Nanjing, China | C3238DJ160_2  C3238DJ160_1  C3238DJ160_3 |

**Table S5: Primers for RT-PCR**

| **Names** | **Sequence (5’-3’)** |
| --- | --- |
| BRG1-F  BRG1-R  PRMT1-F  PRMT1-R  EGFR-F  EGFR-R  TNS4-F  TNS4-R  GAPDH-F  GAPDH-R | GACCAGCACTCCCAAGGTTAC  CTGGCCCGGAAGACATCTG  CTTTGACTCCTACGCACACTT  GTGCCGGTTATGAAACATGGA  AGGCACGAGTAACAAGCTCAC  ATGAGGACATAACCAGCCACC  AGGACACCAGAACTCCGTTCA  TCTCGGGTGATGTTTGGCTTA  GAAGGTGAAGGTCGGAG  GAAGATGGTGATGGGATTTC |

**Table S6: Primers for ChIP**

| **Names** | **Sequence (5’-3’)** |
| --- | --- |
| EGFR-ChIP-F  EGFR-ChIP -R  TNS4-ChIP -F  TNS4-ChIP -R | CTCCTCAGGGCACCCGCTC  CCAGGCGGCGGAGGAGGGATC  CACTGGCATCCTGCTTTGTCAAG  GTTCCTGCTTGGGCTTCTCGTG |

**Table S7:** Viruses

| **Name** | **Source** |
| --- | --- |
| Mouse-LV-PRMT1-RNAi  Mouse-LV-Control-RNAi | Shanghai Genechem Co., Ltd.  Shanghai Genechem Co., Ltd. |

**Table S8:** Tissue Arrays

| **Name** | **Source** |
| --- | --- |
| HColA180Su14 (90 colorectal tumor tissues from patients, 90 adjacent tissues) | Shang Hai Outdo Biotech Co, Ltd. |

**Table S9: List of proteins identified by Mass Spectrometry**

| **No.** | **%Cov(95)** | **Accession**|**Name** | **Peptides(95%)** |
| --- | --- | --- | --- |
| 1 | 49.87 | sp|Q13085|ACACA_HUMAN | 141.00 |
| 2 | 39.90 | sp|P11498|PYC_HUMAN | 55.00 |
| 3 | 21.16 | sp|O75643|U520_HUMAN | 40.00 |
| 4 | 9.01 | sp|P78527|PRKDC_HUMAN | 34.00 |
| 5 | 47.83 | tr|H6VRF9|H6VRF9_HUMAN | 66.00 |
| 6 | 22.85 | sp|Q13428|TCOF_HUMAN | 51.00 |
| 7 | 15.46 | sp|Q6P2Q9|PRP8_HUMAN | 33.00 |
| 8 | 29.21 | sp|Q08211|DHX9_HUMAN | 39.00 |
| 9 | 44.38 | tr|B2RDE0|B2RDE0_HUMAN | 40.00 |
| 10 | 30.45 | tr|H7BXY3|H7BXY3_HUMAN | 32.00 |
| **^#^11** | **17.20** | **sp|P60264**|SMARCA4_**HUMAN** | **29.00** |
| 12 | 38.70 | sp|Q9NR30|DDX21_HUMAN | 32.00 |
| 13 | 23.64 | sp|Q9H6S0|YTDC2_HUMAN | 28.00 |
| 14 | 23.86 | sp|Q16531|DDB1_HUMAN | 24.00 |
| 15 | 46.39 | sp|P35527|K1C9_HUMAN | 39.00 |
| 16 | 44.34 | tr|E9PKE3|E9PKE3_HUMAN | 29.00 |
| 17 | 27.06 | sp|Q15029|U5S1_HUMAN | 21.00 |
| 18 | 30.59 | sp|Q92841|DDX17_HUMAN | 28.00 |
| 19 | 40.41 | sp|P52272|HNRPM_HUMAN | 49.00 |
| 20 | 40.69 | sp|Q96RQ3|MCCA_HUMAN | 30.00 |
| 21 | 44.05 | sp|Q9HCC0|MCCB_HUMAN | 33.00 |
| 22 | 9.60 | sp|P49327|FAS_HUMAN | 20.00 |
| 23 | 38.70 | sp|P13645|K1C10_HUMAN | 31.00 |
| 24 | 23.61 | sp|Q9H0A0|NAT10_HUMAN | 24.00 |
| 25 | 33.18 | sp|P11940|PABP1_HUMAN | 23.00 |
| 26 | 18.25 | sp|O75533|SF3B1_HUMAN | 20.00 |
| 27 | 19.28 | sp|P53621|COPA_HUMAN | 19.00 |
| 28 | 33.64 | sp|P11021|GRP78_HUMAN | 25.00 |
| 29 | 13.49 | sp|Q00610|CLH1_HUMAN | 18.00 |
| 30 | 9.59 | sp|Q9UQ35|SRRM2_HUMAN | 21.00 |
| 31 | 37.72 | sp|P35908|K22E_HUMAN | 30.00 |
| 32 | 22.14 | sp|O43143|DHX15_HUMAN | 18.00 |
| 33 | 17.34 | sp|Q15393|SF3B3_HUMAN | 19.00 |
| 34 | 31.53 | tr|B3KM80|B3KM80_HUMAN | 19.00 |
| 35 | 33.83 | tr|B7Z4V2|B7Z4V2_HUMAN | 19.00 |
| 36 | 29.21 | tr|B2R8Z8|B2R8Z8_HUMAN | 17.00 |
| 37 | 12.35 | sp|Q9BQG0|MBB1A_HUMAN | 15.00 |
| 38 | 16.55 | sp|P35251|RFC1_HUMAN | 16.00 |
| 39 | 21.99 | tr|B4DGL0|B4DGL0_HUMAN | 16.00 |
| 40 | 13.49 | sp|Q6P158|DHX57_HUMAN | 17.00 |
| 41 | 23.59 | sp|Q13263|TIF1B_HUMAN | 21.00 |
| 42 | 34.95 | sp|P0DMV8|HS71A_HUMAN | 23.00 |
| 43 | 25.25 | sp|Q96EY7|PTCD3_HUMAN | 18.00 |
| 44 | 31.61 | sp|O00425|IF2B3_HUMAN | 17.00 |
| 45 | 18.07 | sp|P13639|EF2_HUMAN | 14.00 |
| 46 | 32.33 | tr|B4DY90|TUBB_HUMAN | 17.00 |
| 47 | 18.97 | sp|P46087|NOP2_HUMAN | 15.00 |
| 48 | 28.06 | sp|Q6P1J9|CDC73_HUMAN | 13.00 |
| 49 | 23.41 | sp|Q9UJS0|CMC2_HUMAN | 14.00 |
| 50 | 14.20 | sp|P42285|SK2L2_HUMAN | 13.00 |
| 51 | 10.29 | sp|P52701|MSH6_HUMAN | 11.00 |
| 52 | 10.93 | sp|Q9UPW5|CBPC1_HUMAN | 11.00 |
| 53 | 21.14 | sp|P33993|MCM7_HUMAN | 13.00 |
| 54 | 13.79 | sp|O14617|AP3D1_HUMAN | 13.00 |
| 55 | 12.20 | sp|Q9P2E9|RRBP1_HUMAN | 13.00 |
| 56 | 12.79 | sp|Q6PD62|CTR9_HUMAN | 15.00 |
| 57 | 17.86 | tr|A0A1W2PP35_HUMAN | 13.00 |
| 58 | 29.42 | tr|A0A024R2Z6_HUMAN | 12.00 |
| 58 | 26.59 | sp|Q9BVP2|GNL3_HUMAN | 11.00 |
| 59 | 15.90 | tr|B4E0B9|B4E0B9_HUMAN | 12.00 |
| 60 | 28.99 | sp|P17844|DDX5_HUMAN | 23.00 |
| 61 | 26.49 | tr|B4DRA0|B4DRA0_HUMAN | 14.00 |
| 62 | 29.11 | sp|Q9Y2X3|NOP58_HUMAN | 13.00 |
| 63 | 19.77 | tr|Q96L66|Q96L66_HUMAN | 13.00 |
| 64 | 6.12 | sp|Q12789|TF3C1_HUMAN | 12.00 |
| 65 | 14.73 | tr|B4E2T6|B4E2T6_HUMAN | 11.00 |
| 66 | 11.34 | sp|P55265|DSRAD_HUMAN | 15.00 |
| 67 | 12.92 | sp|Q8IZL8|PELP1_HUMAN | 10.00 |
| 68 | 7.82 | tr|Q59G75|Q59G75_HUMAN | 9.00 |
| 69 | 8.30 | sp|P35580|MYH10_HUMAN | 12.00 |
| 70 | 10.42 | tr|A0A0S2Z5J4_HUMAN | 10.00 |
| 71 | 25.42 | sp|O00567|NOP56_HUMAN | 14.00 |
| 72 | 14.10 | tr|A0A024R169_HUMAN | 10.00 |
| 73 | 25.42 | sp|P02533|K1C14_HUMAN | 10.00 |
| 74 | 15.84 | sp|Q9BSC4|NOL10_HUMAN | 11.00 |
| 75 | 11.80 | tr|F4ZW66|F4ZW66_HUMAN | 10.00 |
| 76 | 20.47 | tr|Q59GX6|Q59GX6_HUMAN | 17.00 |
| 77 | 22.22 | tr|A0A024R0H6_HUMAN | 11.00 |
| 78 | 8.60 | sp|P07814|SYEP_HUMAN | 10.00 |
| 79 | 21.86 | tr|B7Z5E7|B7Z5E7_HUMAN | 9.00 |
| 80 | 11.11 | sp|Q9H2U1|DHX36_HUMAN | 9.00 |
| 81 | 16.82 | tr|B1AHC9|B1AHC9_HUMAN | 8.00 |
| 82 | 10.29 | sp|Q8IY37|DHX37_HUMAN | 10.00 |
| 83 | 12.04 | tr|Q5JR04|Q5JR04_HUMAN | 10.00 |
| 84 | 14.33 | sp|P51114|FXR1_HUMAN | 9.00 |
| 85 | 8.59 | sp|Q9NZB2|F120A_HUMAN | 9.00 |
| 86 | 20.34 | tr|B4DVI7|B4DVI7_HUMAN | 9.00 |
| 87 | 17.96 | tr|Q6IBR0|Q6IBR0_HUMAN | 9.00 |
| 88 | 27.35 | sp|O76021|RL1D1_HUMAN | 10.00 |
| 89 | 12.29 | tr|B3GQE6|B3GQE6_HUMAN | 9.00 |
| 90 | 24.11 | sp|P48668|K2C6C_HUMAN | 14.00 |
| 91 | 13.33 | sp|P53618|COPB_HUMAN | 11.00 |
| 92 | 24.09 | sp|Q9NZI8|IF2B1_HUMAN | 14.00 |
| 93 | 11.64 | sp|P09874|PARP1_HUMAN | 10.00 |
| 94 | 14.30 | tr|A8K9U6|A8K9U6_HUMAN | 9.00 |
| 95 | 11.86 | tr|B7Z3V1|B7Z3V1_HUMAN | 9.00 |
| 96 | 8.54 | sp|O00411|RPOM_HUMAN | 9.00 |
| 97 | 10.04 | sp|Q6PKG0|LARP1_HUMAN | 9.00 |
| 98 | 18.30 | sp|Q9NVI7|ATD3A_HUMAN | 10.00 |
| 99 | 19.16 | tr|Q53HU0|Q53HU0_HUMAN | 9.00 |
| **^#^100** | **34.13** | **sp|P60709|ACTB_HUMAN** | **10.00** |
| 101 | 16.29 | tr|Q59H77|Q59H77_HUMAN | 8.00 |
| 102 | 13.63 | tr|E9KL44|E9KL44_HUMAN | 8.00 |
| 103 | 21.21 | sp|O95831|AIFM1_HUMAN | 9.00 |
| 104 | 17.79 | tr|Q96N86|Q96N86_HUMAN | 8.00 |
| 105 | 3.34 | sp|P15924|DESP_HUMAN | 9.00 |
| 106 | 8.69 | sp|Q9Y2W1|TR150_HUMAN | 8.00 |
| 107 | 16.69 | sp|P61221|ABCE1_HUMAN | 9.00 |
| 108 | 27.64 | tr|B3KT06|B3KT06_HUMAN | 8.00 |
| 109 | 15.94 | tr|B5MCF9|B5MCF9_HUMAN | 9.00 |
| 110 | 10.39 | tr|A8MXP9|A8MXP9_HUMAN | 7.00 |
| 111 | 15.81 | sp|Q4G0J3|LARP7_HUMAN | 8.00 |
| 112 | 5.04 | sp|Q9Y4B6|DCAF1_HUMAN | 8.00 |
| 113 | 11.69 | sp|O14654|IRS4_HUMAN | 9.00 |
| 114 | 35.26 | tr|Q8WVW5|Q8WVW5_HUMAN | 10.00 |
| 115 | 12.97 | sp|Q9BVJ6|UT14A_HUMAN | 8.00 |
| 116 | 10.45 | sp|Q99575|POP1_HUMAN | 8.00 |
| 117 | 12.62 | tr|B4DLM8|B4DLM8_HUMAN | 8.00 |
| 118 | 9.40 | tr|A8K897|A8K897_HUMAN | 7.00 |
| 119 | 13.94 | sp|P54136|SYRC_HUMAN | 8.00 |
| 120 | 11.85 | sp|Q9Y4W2|LAS1L_HUMAN | 7.00 |
| 121 | 4.42 | tr|B7ZKR9|B7ZKR9_HUMAN | 7.00 |
| 122 | 15.63 | tr|J3KNN5|J3KNN5_HUMAN | 8.00 |
| 123 | 12.62 | tr|B4DRS4|B4DRS4_HUMAN | 9.00 |
| 124 | 10.94 | tr|Q7RU04|Q7RU04_HUMAN | 7.00 |
| 125 | 22.84 | tr|Q5EC54|Q5EC54_HUMAN | 7.00 |
| 126 | 10.22 | sp|Q8TDD1|DDX54_HUMAN | 8.00 |
| 127 | 4.13 | sp|Q92621|NU205_HUMAN | 9.00 |
| 128 | 11.27 | sp|Q96KR1|ZFR_HUMAN | 7.00 |
| 129 | 11.45 | tr|Q6NUN2|Q6NUN2_HUMAN | 7.00 |
| 130 | 8.61 | sp|O94906|PRP6_HUMAN | 7.00 |
| 131 | 5.85 | tr|B7ZLZ7|B7ZLZ7_HUMAN | 6.00 |
| 132 | 3.80 | sp|Q14690|RRP5_HUMAN | 7.00 |
| 133 | 14.86 | sp|Q08J23|NSUN2_HUMAN | 8.00 |
| 134 | 8.73 | tr|A0A0S2Z3L2_HUMAN | 7.00 |
| 135 | 15.10 | tr|A0A0S2Z4Z0_HUMAN | 7.00 |
| 136 | 10.96 | sp|Q15397|PUM3_HUMAN | 6.00 |
| 137 | 9.92 | sp|Q8IY81|SPB1_HUMAN | 7.00 |
| 138 | 15.76 | sp|O43175|SERA_HUMAN | 7.00 |
| 139 | 7.73 | tr|M0R3F6|M0R3F6_HUMAN | 6.00 |
| 140 | 20.51 | sp|P13647|K2C5_HUMAN | 14.00 |
| 141 | 5.08 | sp|Q12769|NU160_HUMAN | 6.00 |
| 142 | 19.75 | tr|B5BU25|B5BU25_HUMAN | 9.00 |
| 143 | 11.26 | tr|B2RDN4|B2RDN4_HUMAN | 7.00 |
| 144 | 9.36 | tr|X5D2E5|X5D2E5_HUMAN | 8.00 |
| 145 | 7.40 | tr|A8K8U1|A8K8U1_HUMAN | 7.00 |
| 146 | 6.54 | sp|O14980|XPO1_HUMAN | 6.00 |
| 147 | 7.68 | tr|A8KA56|A8KA56_HUMAN | 6.00 |
| 148 | 10.25 | tr|A0A090N7Y2_HUMAN | 8.00 |
| 149 | 8.66 | tr|E9PPJ0|E9PPJ0_HUMAN | 7.00 |
| 150 | 5.99 | tr|B7Z6H4|B7Z6H4_HUMAN | 7.00 |
| 151 | 30.65 | tr|A0A024RAQ1_HUMAN | 7.00 |
| 152 | 7.86 | tr|V9GYM8|V9GYM8_HUMAN | 7.00 |
| 153 | 2.32 | sp|Q8N3C0|ASCC3_HUMAN | 5.00 |
| 154 | 9.21 | sp|P06737|PYGL_HUMAN | 8.00 |
| 155 | 8.60 | tr|A0A024R1A3_HUMAN | 6.00 |
| 156 | 10.54 | tr|A0A024R753_HUMAN | 7.00 |
| 157 | 4.50 | sp|Q10570|CPSF1_HUMAN | 6.00 |
| 158 | 12.13 | tr|B4DSH1|B4DSH1_HUMAN | 6.00 |
| 159 | 1.44 | sp|Q14204|DYHC1_HUMAN | 6.00 |
| 160 | 17.46 | tr|Q59GY2|Q59GY2_HUMAN | 7.00 |
| 161 | 12.80 | sp|Q9NY93|DDX56_HUMAN | 7.00 |
| 162 | 7.55 | sp|Q9BZJ0|CRNL1_HUMAN | 6.00 |
| 163 | 7.51 | sp|P55786|PSA_HUMAN | 7.00 |
| 164 | 10.97 | tr|A0A087WTP3_HUMAN | 6.00 |
| 165 | 23.94 | tr|A0A024R814|A0A024R814_HUMAN | 6.00 |
| 166 | 17.16 | tr|B4DW97|B4DW97_HUMAN | 12.00 |
| 167 | 4.75 | tr|E9PD53|E9PD53_HUMAN | 5.00 |
| 168 | 5.10 | tr|Q4LE36|Q4LE36_HUMAN | 4.00 |
| 169 | 5.82 | sp|Q9H9Y6|RPA2_HUMAN | 6.00 |
| 170 | 8.96 | tr|A0A024RAH8_HUMAN | 5.00 |
| **^#^171** | **5.04** | **sp|O60264|**SMCA5_**HUMAN** | **5.00** |
| 172 | 6.63 | tr|Q59EC7|Q59EC7_HUMAN | 5.00 |
| 173 | 7.36 | tr|Q53HS0|Q53HS0_HUMAN | 5.00 |
| 174 | 12.48 | tr|B2RAX6|B2RAX6_HUMAN | 5.00 |
| 175 | 22.18 | tr|B4DMB1|B4DMB1_HUMAN | 13.00 |
| 176 | 8.29 | tr|Q53HJ4|Q53HJ4_HUMAN | 6.00 |
| 177 | 12.62 | tr|B4DPP6|B4DPP6_HUMAN | 10.00 |
| 178 | 10.48 | sp|Q5BKZ1|ZN326_HUMAN | 5.00 |
| 179 | 15.60 | sp|P51116|FXR2_HUMAN | 8.00 |
| 180 | 7.82 | tr|Q59EF6|Q59EF6_HUMAN | 5.00 |
| 181 | 9.23 | sp|P17858|PFKAL_HUMAN | 6.00 |
| 182 | 9.65 | tr|B7Z4B8|B7Z4B8_HUMAN | 5.00 |
| 183 | 8.76 | sp|O15371|EIF3D_HUMAN | 5.00 |
| 184 | 3.96 | tr|J3QS41|J3QS41_HUMAN | 6.00 |
| 185 | 5.81 | sp|Q9BPX3|CND3_HUMAN | 5.00 |
| 186 | 3.63 | tr|B2RTS4|B2RTS4_HUMAN | 5.00 |
| 187 | 6.67 | sp|P56192|SYMC_HUMAN | 6.00 |
| 188 | 3.21 | tr|A0A024R1N1_HUMAN | 5.00 |
| 189 | 20.30 | tr|Q6IQ30|Q6IQ30_HUMAN | 13.00 |
| 190 | 14.49 | tr|B4DMB5|B4DMB5_HUMAN | 6.00 |
| 191 | 10.89 | sp|O60832|DKC1_HUMAN | 5.00 |
| 192 | 6.35 | tr|A1KYQ7|A1KYQ7_HUMAN | 5.00 |
| 193 | 6.89 | sp|Q01780|EXOSX_HUMAN | 5.00 |
| 194 | 13.68 | sp|Q96GA3|LTV1_HUMAN | 6.00 |
| 195 | 7.76 | tr|B4DNM0|B4DNM0_HUMAN | 5.00 |
| 196 | 9.30 | sp|Q5QP82|DCA10_HUMAN | 4.00 |
| 197 | 5.41 | sp|Q9H6R4|NOL6_HUMAN | 5.00 |
| 198 | 7.78 | sp|Q14684|RRP1B_HUMAN | 6.00 |
| 199 | 5.03 | sp|Q9Y678|COPG1_HUMAN | 4.00 |
| 200 | 7.31 | tr|B2RBR9|B2RBR9_HUMAN | 5.00 |
| 201 | 8.34 | tr|V9HWP2|V9HWP2_HUMAN | 7.00 |
| 202 | 5.47 | tr|Q8NDH0|Q8NDH0_HUMAN | 4.00 |
| 203 | 5.42 | sp|Q9Y5Q9|TF3C3_HUMAN | 4.00 |
| 204 | 7.60 | tr|Q71V07|Q71V07_HUMAN | 4.00 |
| 205 | 4.56 | sp|Q96ST3|SIN3A_HUMAN | 5.00 |
| 206 | 8.42 | sp|Q12788|TBL3_HUMAN | 5.00 |
| 207 | 3.92 | tr|Q9HC39|Q9HC39_HUMAN | 4.00 |
| 208 | 5.33 | sp|P13010|XRCC5_HUMAN | 4.00 |
| 209 | 7.54 | sp|Q15046|SYK_HUMAN | 4.00 |
| 210 | 6.34 | tr|A0A024R464_HUMAN | 4.00 |
| 211 | 12.46 | sp|Q6UN15|FIP1_HUMAN | 6.00 |
| 212 | 21.99 | sp|P08779|K1C16_HUMAN | 10.00 |
| 213 | 15.71 | tr|E7EQ64|E7EQ64_HUMAN | 12.00 |
| 214 | 7.30 | tr|C4B4C6|C4B4C6_HUMAN | 4.00 |
| 215 | 10.54 | sp|Q9NY61|AATF_HUMAN | 4.00 |
| 216 | 8.35 | tr|Q05DU1|Q05DU1_HUMAN | 4.00 |
| 217 | 9.94 | tr|B2RDI5|B2RDI5_HUMAN | 6.00 |
| 218 | 7.52 | sp|Q9UII4|HERC5_HUMAN | 6.00 |
| 219 | 7.50 | tr|A8K905|A8K905_HUMAN | 5.00 |
| 220 | 6.61 | tr|B4DUT2|B4DUT2_HUMAN | 4.00 |
| 221 | 6.67 | sp|Q709F0|ACD11_HUMAN | 5.00 |
| 222 | 9.12 | tr|B5BU99|B5BU99_HUMAN | 5.00 |
| 223 | 17.01 | tr|V9HVZ4|V9HVZ4_HUMAN | 5.00 |
| 224 | 3.14 | sp|Q8NI27|THOC2_HUMAN | 5.00 |
| 225 | 18.03 | tr|K9JA46|K9JA46_HUMAN | 14.00 |
| 226 | 6.07 | tr|D3DPS3|D3DPS3_HUMAN | 5.00 |
| 227 | 7.81 | tr|Q5BKZ2|Q5BKZ2_HUMAN | 4.00 |
| 228 | 2.24 | tr|B2RWN5|B2RWN5_HUMAN | 4.00 |
| 229 | 7.03 | tr|Q8TBR3|Q8TBR3_HUMAN | 4.00 |
| 230 | 4.85 | tr|B2RAH5|B2RAH5_HUMAN | 5.00 |
| 231 | 4.78 | sp|Q9Y5B9|SP16H_HUMAN | 4.00 |
| 232 | 11.53 | tr|V9HW31|V9HW31_HUMAN | 5.00 |
| 233 | 5.99 | tr|Q53GS0|Q53GS0_HUMAN | 4.00 |
| 234 | 11.59 | tr|V9HW96|V9HW96_HUMAN | 5.00 |
| 235 | 3.51 | sp|Q03701|CEBPZ_HUMAN | 4.00 |
| 236 | 11.03 | tr|V9HW26|V9HW26_HUMAN | 5.00 |
| 237 | 9.62 | tr|B4DPW9|B4DPW9_HUMAN | 5.00 |
| 238 | 3.23 | tr|E5KNY5|E5KNY5_HUMAN | 5.00 |
| 239 | 4.83 | sp|P50570|DYN2_HUMAN | 4.00 |
| 240 | 8.33 | tr|A0A024R1S5_HUMAN | 5.00 |
| 241 | 7.96 | sp|Q5SSJ5|HP1B3_HUMAN | 4.00 |
| 242 | 10.54 | tr|Q5H909|Q5H909_HUMAN | 5.00 |
| 243 | 9.00 | sp|P14866|HNRPL_HUMAN | 4.00 |
| 244 | 17.65 | tr|Q9HBB3|Q9HBB3_HUMAN | 4.00 |
| 245 | 6.80 | tr|E9PQN2|E9PQN2_HUMAN | 5.00 |
| 246 | 6.43 | tr|Q8TDR3|Q8TDR3_HUMAN | 4.00 |
| 247 | 4.54 | sp|Q5C9Z4|NOM1_HUMAN | 3.00 |
| 248 | 8.54 | tr|A6NLN1|A6NLN1_HUMAN | 3.00 |
| 249 | 6.22 | sp|Q71RC2|LARP4_HUMAN | 4.00 |
| 250 | 6.37 | tr|Q4ZG57|Q4ZG57_HUMAN | 5.00 |
| 251 | 9.61 | tr|V9HWB8|V9HWB8_HUMAN | 4.00 |
| 252 | 7.21 | sp|Q8N8A6|DDX51_HUMAN | 5.00 |
| 253 | 4.60 | tr|A0A0D9SER5_HUMAN | 4.00 |
| 254 | 10.63 | sp|Q9H4L4|SENP3_HUMAN | 5.00 |
| 255 | 1.89 | sp|Q5SRE5|NU188_HUMAN | 5.00 |
| 256 | 1.47 | sp|P46013|KI67_HUMAN | 3.00 |
| 257 | 10.37 | tr|A0A024R4F1|A0A024R4F1_HUMAN | 3.00 |
| 258 | 4.64 | tr|B9A067|B9A067_HUMAN | 3.00 |
| 259 | 6.28 | tr|B2RDD7|B2RDD7_HUMAN | 4.00 |
| 260 | 3.76 | sp|Q9H501|ESF1_HUMAN | 3.00 |
| 261 | 4.35 | tr|F2Z2W7|F2Z2W7_HUMAN | 3.00 |
| 262 | 11.43 | tr|A0A0J9YXX5_HUMAN | 3.00 |
| 263 | 13.72 | tr|B3KMV8|B3KMV8_HUMAN | 8.00 |
| 264 | 3.55 | sp|Q5JTZ9|SYAM_HUMAN | 3.00 |
| 265 | 17.65 | sp|P16402|H13_HUMAN | 3.00 |
| 266 | 12.91 | tr|Q9NZS6|Q9NZS6_HUMAN | 5.00 |
| 267 | 6.98 | tr|A0A090N8Y2_HUMAN | 4.00 |
| 268 | 4.90 | tr|A0A024RDS1_HUMAN | 3.00 |
| 269 | 3.75 | sp|Q9Y4C8|RBM19_HUMAN | 3.00 |
| 270 | 4.14 | tr|H9KV75|H9KV75_HUMAN | 3.00 |
| 271 | 2.17 | tr|H0YM23|H0YM23_HUMAN | 5.00 |
| 272 | 2.83 | tr|A0A024R7L8_HUMAN | 3.00 |
| 273 | 12.07 | tr|B4DLD4|B4DLD4_HUMAN | 3.00 |
| 274 | 5.13 | tr|A8K4T8|A8K4T8_HUMAN | 3.00 |
| 275 | 6.87 | tr|Q53H10|Q53H10_HUMAN | 3.00 |
| 276 | 3.89 | sp|Q9UKN8|TF3C4_HUMAN | 3.00 |
| 277 | 6.68 | tr|H3BS42|H3BS42_HUMAN | 3.00 |
| 278 | 6.83 | tr|Q6NWZ1|Q6NWZ1_HUMAN | 3.00 |
| 279 | 5.18 | sp|Q7Z417|NUFP2_HUMAN | 3.00 |
| 280 | 7.41 | tr|Q9BSV4|Q9BSV4_HUMAN | 3.00 |
| 281 | 2.29 | sp|Q9UPY3|DICER_HUMAN | 4.00 |
| 282 | 2.26 | tr|Q9Y5S5|Q9Y5S5_HUMAN | 4.00 |
| 283 | 3.54 | sp|P49736|MCM2_HUMAN | 3.00 |
| 284 | 8.14 | sp|O00763|ACACB_HUMAN | 24.00 |
| 285 | 5.47 | sp|Q9Y3T9|NOC2L_HUMAN | 4.00 |
| 286 | 1.93 | sp|Q8IZH2|XRN1_HUMAN | 4.00 |
| 287 | 2.75 | tr|Q6URC4|Q6URC4_HUMAN | 3.00 |
| 288 | 6.81 | tr|B2RE46|B2RE46_HUMAN | 3.00 |
| 289 | 7.44 | tr|A0A087X2G1_HUMAN | 4.00 |
| 290 | 2.03 | sp|O60287|NPA1P_HUMAN | 4.00 |
| 291 | 5.22 | tr|B3KN45|B3KN45_HUMAN | 3.00 |
| 292 | 2.21 | tr|L0DYZ1|L0DYZ1_THIND | 3.00 |
| 293 | 2.09 | tr|B4DER1|B4DER1_HUMAN | 2.00 |
| 294 | 7.00 | tr|Q6IBT3|Q6IBT3_HUMAN | 3.00 |
| 295 | 3.20 | tr|Q7Z5T5|Q7Z5T5_HUMAN | 2.00 |
| 296 | 2.35 | sp|P11388|TOP2A_HUMAN | 3.00 |
| 297 | 4.53 | tr|B4DGM5|B4DGM5_HUMAN | 3.00 |
| 298 | 5.44 | tr|B4DDM5|B4DDM5_HUMAN | 3.00 |
| 299 | 5.58 | tr|B2RAU8|B2RAU8_HUMAN | 3.00 |
| 300 | 10.53 | tr|Q6NVC0|Q6NVC0_HUMAN | 3.00 |
| 301 | 3.87 | sp|Q8TCJ2|STT3B_HUMAN | 3.00 |
| 302 | 3.61 | sp|Q1KMD3|HNRL2_HUMAN | 2.00 |
| 303 | 4.26 | sp|Q15572|TAF1C_HUMAN | 3.00 |
| 304 | 8.37 | tr|Q6FI03|Q6FI03_HUMAN | 3.00 |
| 305 | 3.27 | sp|Q8IWX8|CHERP_HUMAN | 3.00 |
| 306 | 6.68 | tr|D3DUE7|D3DUE7_HUMAN | 3.00 |
| 307 | 3.58 | sp|Q8NI36|WDR36_HUMAN | 3.00 |
| 308 | 2.67 | tr|A0A024QZH6_HUMAN | 3.00 |
| 309 | 4.96 | tr|F8WAJ0|F8WAJ0_HUMAN | 3.00 |
| 310 | 9.16 | tr|Q9BV61|Q9BV61_HUMAN | 5.00 |
| 311 | 2.69 | tr|Q05CW6|Q05CW6_HUMAN | 2.00 |
| 312 | 2.53 | sp|O15042|SR140_HUMAN | 2.00 |
| 313 | 4.38 | tr|Q5T0F3|Q5T0F3_HUMAN | 4.00 |
| 314 | 5.97 | tr|B4DZ22|B4DZ22_HUMAN | 3.00 |
| 315 | 2.13 | tr|Q8N5A0|Q8N5A0_HUMAN | 2.00 |
| 316 | 6.13 | tr|A0A024R983_HUMAN | 3.00 |
| 317 | 3.88 | sp|O95394|AGM1_HUMAN | 2.00 |
| 318 | 3.37 | tr|A0A0A0MRP0_HUMAN | 2.00 |
| 319 | 8.53 | tr|Q5T7U1|Q5T7U1_HUMAN | 3.00 |
| 320 | 2.49 | tr|A8KA19|A8KA19_HUMAN | 2.00 |
| 321 | 1.74 | sp|P42695|CNDD3_HUMAN | 2.00 |
| 322 | 2.26 | tr|B7ZMF2|B7ZMF2_HUMAN | 3.00 |
| 323 | 5.11 | tr|B7ZM99|B7ZM99_HUMAN | 4.00 |
| 324 | 3.93 | tr|B3KPV5|B3KPV5_HUMAN | 2.00 |
| 325 | 1.38 | sp|Q69YN4|VIR_HUMAN | 2.00 |
| 326 | 4.97 | tr|A0A0S2Z487_HUMAN | 3.00 |
| 327 | 5.19 | sp|Q15020|SART3_HUMAN | 3.00 |
| 328 | 3.51 | sp|Q9Y3Z3|SAMH1_HUMAN | 2.00 |
| 329 | 2.42 | sp|P38935|SMBP2_HUMAN | 2.00 |
| 330 | 2.91 | tr|C9J2Y9|C9J2Y9_HUMAN | 3.00 |
| 331 | 2.45 | sp|Q9UPP1|PHF8_HUMAN | 2.00 |
| 332 | 5.88 | sp|Q7Z4Q2|HEAT3_HUMAN | 3.00 |
| 333 | 5.26 | tr|Q53F02|Q53F02_HUMAN | 3.00 |
| 334 | 7.69 | tr|Q59GI7|Q59GI7_HUMAN | 2.00 |
| 335 | 6.59 | tr|Q6IAU5|Q6IAU5_HUMAN | 3.00 |
| 336 | 7.50 | tr|Q71US4|Q71US4_HUMAN | 3.00 |
| 337 | 1.47 | tr|A0A087WWE2_HUMAN | 3.00 |
| 338 | 2.10 | tr|Q59HH3|Q59HH3_HUMAN | 2.00 |
| 339 | 3.22 | tr|B3KPM8|B3KPM8_HUMAN | 3.00 |
| 340 | 1.93 | tr|B3KMR5|B3KMR5_HUMAN | 2.00 |
| 341 | 3.22 | sp|Q9UGR2|Z3H7B_HUMAN | 3.00 |
| 342 | 8.58 | tr|Q6I9S2|Q6I9S2_HUMAN | 3.00 |
| 343 | 9.38 | tr|A8K559|A8K559_HUMAN | 3.00 |
| 344 | 3.54 | tr|Q53FG5|Q53FG5_HUMAN | 2.00 |
| 345 | 2.92 | tr|B4DLA6|B4DLA6_HUMAN | 2.00 |
| 346 | 3.82 | sp|Q96SB4|SRPK1_HUMAN | 2.00 |
| 347 | 13.64 | tr|Q6IPH7|Q6IPH7_HUMAN | 2.00 |
| 348 | 2.99 | tr|Q53ER0|Q53ER0_HUMAN | 2.00 |
| 349 | 2.87 | tr|Q58F09|Q58F09_HUMAN | 2.00 |
| 350 | 12.50 | sp|Q04695|K1C17_HUMAN | 6.00 |
| 351 | 2.25 | tr|A0A140VK66_HUMAN | 2.00 |
| 352 | 13.68 | tr|J3QQ67|J3QQ67_HUMAN | 2.00 |
| 353 | 5.11 | tr|J3KPP4|J3KPP4_HUMAN | 3.00 |
| 354 | 3.13 | tr|H0YJ34|H0YJ34_HUMAN | 2.00 |
| 355 | 35.45 | tr|Q59EM9|Q59EM9_HUMAN | 2.00 |
| 356 | 4.96 | tr|D6RIY6|D6RIY6_HUMAN | 2.00 |
| 357 | 5.17 | tr|J3QT46|J3QT46_HUMAN | 2.00 |
| 358 | 11.48 | tr|B0QYV1|B0QYV1_HUMAN | 2.00 |
| 359 | 4.09 | tr|Q5HYL4|Q5HYL4_HUMAN | 2.00 |
| 360 | 12.73 | sp|P81605|DCD_HUMAN | 2.00 |
| 361 | 4.17 | tr|Q6MZV5|Q6MZV5_HUMAN | 2.00 |
| 362 | 8.08 | tr|Q5U077|Q5U077_HUMAN | 2.00 |
| 363 | 2.91 | sp|Q8NB90|SPAT5_HUMAN | 2.00 |
| 364 | 2.03 | sp|Q14692|BMS1_HUMAN | 2.00 |
| 365 | 3.66 | tr|G3V153|G3V153_HUMAN | 2.00 |
| 366 | 3.23 | tr|Q9UK43|Q9UK43_HUMAN | 2.00 |
| 367 | 2.22 | tr|F8W726|F8W726_HUMAN | 2.00 |
| 368 | 4.81 | tr|V9HW37|V9HW37_HUMAN | 2.00 |
| 369 | 4.34 | sp|Q01813|PFKAP_HUMAN | 3.00 |
| 370 | 4.26 | tr|A8MST6|A8MST6_HUMAN | 3.00 |
| 371 | 8.70 | tr|X5DQX7|X5DQX7_HUMAN | 5.00 |
| 372 | 4.48 | tr|A8K2K2|A8K2K2_HUMAN | 2.00 |
| 373 | 1.25 | sp|O75165|DJC13_HUMAN | 2.00 |
| 374 | 6.68 | tr|A0A024R254_HUMAN | 4.00 |
| 375 | 4.10 | sp|P20700|LMNB1_HUMAN | 2.00 |
| 376 | 6.13 | sp|Q9H0W5|CCDC8_HUMAN | 2.00 |
| 377 | 0.71 | tr|E1NZA1|E1NZA1_HUMAN | 2.00 |
| 378 | 4.19 | tr|A0A024RC67_HUMAN | 2.00 |
| 379 | 4.05 | tr|B4DWL1|B4DWL1_HUMAN | 2.00 |
| 380 | 6.83 | sp|P05787|K2C8_HUMAN | 3.00 |
| 381 | 3.07 | sp|Q96P70|IPO9_HUMAN | 2.00 |
| 382 | 1.92 | tr|X5D2F4|X5D2F4_HUMAN | 2.00 |
| 383 | 4.39 | tr|V9HWH7|V9HWH7_HUMAN | 2.00 |
| 384 | 4.46 | sp|Q9BVI4|NOC4L_HUMAN | 2.00 |
| 385 | 7.32 | tr|E7EX29|E7EX29_HUMAN | 2.00 |
| 386 | 4.22 | tr|F5H5U2|F5H5U2_HUMAN | 2.00 |
| 387 | 3.47 | tr|I6TRR8|I6TRR8_HUMAN | 3.00 |
| 388 | 4.01 | sp|P07199|CENPB_HUMAN | 2.00 |
| 389 | 23.20 | tr|J3KQ96|J3KQ96_HUMAN | 50.00 |
| 390 | 2.98 | tr|V9HW80|V9HW80_HUMAN | 2.00 |
| 391 | 1.81 | tr|Q86VX4|Q86VX4_HUMAN | 2.00 |
| 392 | 3.30 | tr|E5KRK5|E5KRK5_HUMAN | 2.00 |
| 393 | 3.80 | tr|B2RE34|B2RE34_HUMAN | 2.00 |
| 394 | 3.44 | sp|P49756|RBM25_HUMAN | 2.00 |
| 395 | 4.99 | tr|Q9BV37|Q9BV37_HUMAN | 2.00 |
| 396 | 10.90 | tr|Q6NZ55|Q6NZ55_HUMAN | 2.00 |
| 397 | 4.51 | sp|P38432|COIL_HUMAN | 2.00 |
| 398 | 5.92 | tr|A0A024R2F4_HUMAN | 3.00 |
| 399 | 5.95 | tr|Q53YD7|Q53YD7_HUMAN | 2.00 |
| 400 | 3.44 | tr|J3KT10|J3KT10_HUMAN | 2.00 |
| 401 | 2.44 | tr|K4DI93|K4DI93_HUMAN | 2.00 |
| 402 | 2.55 | tr|Q5TDG3|Q5TDG3_HUMAN | 2.00 |
| 403 | 4.26 | tr|A0A024R1U0_HUMAN | 2.00 |
| 404 | 3.93 | tr|C9JA69|C9JA69_HUMAN | 3.00 |
| 405 | 1.18 | sp|Q14008|CKAP5_HUMAN | 2.00 |
| 406 | 3.32 | tr|Q8NE02|Q8NE02_HUMAN | 2.00 |
| 407 | 3.84 | sp|Q9Y2R4|DDX52_HUMAN | 2.00 |
| 408 | 2.15 | sp|Q96T37|RBM15_HUMAN | 2.00 |
| 409 | 1.25 | tr|A0A140VJP1_HUMAN | 1.00 |
| 410 | 1.92 | tr|B2RAJ6|B2RAJ6_HUMAN | 2.00 |
| 411 | 5.45 | sp|Q13501|SQSTM_HUMAN | 2.00 |
| 412 | 1.97 | tr|B4DM03|B4DM03_HUMAN | 1.00 |
| 413 | 2.50 | tr|A8QI98|A8QI98_HUMAN | 2.00 |
| 414 | 6.56 | tr|Q96I60|Q96I60_HUMAN | 4.00 |
| 415 | 3.21 | tr|V9HW33|V9HW33_HUMAN | 2.00 |
| 416 | 3.41 | tr|A0A140VKH3_HUMAN | 2.00 |
| 417 | 1.54 | tr|B7Z5S1|B7Z5S1_HUMAN | 2.00 |
| 418 | 5.02 | tr|D9ZGG2|D9ZGG2_HUMAN | 3.00 |
| 419 | 3.07 | tr|A8K5H7|A8K5H7_HUMAN | 2.00 |
| 420 | 3.32 | tr|Q53GX7|Q53GX7_HUMAN | 2.00 |
| 421 | 5.31 | tr|B2R841|B2R841_HUMAN | 2.00 |
| 422 | 6.07 | sp|Q12797|ASPH_HUMAN | 3.00 |
| 423 | 1.21 | sp|Q14669|TRIPC_HUMAN | 2.00 |
| **^#^424** | **2.82** | **tr|Q59GQ7|**SMARCA3_**HUMAN** | **2.00** |
| 425 | 3.32 | sp|Q4VCS5|AMOT_HUMAN | 3.00 |
| 426 | 2.57 | tr|A0A087WZ30_HUMAN | 2.00 |
| 427 | 1.77 | sp|O75419|CDC45_HUMAN | 1.00 |
| 428 | 1.47 | tr|Q68DI0|Q68DI0_HUMAN | 2.00 |
| 429 | 2.61 | tr|A0A0B4J2E5_HUMAN | 2.00 |
| 430 | 4.12 | tr|J3KPF3|J3KPF3_HUMAN | 2.00 |
| 431 | 1.54 | sp|Q6PCB5|RSBNL_HUMAN | 1.00 |
| 432 | 1.09 | sp|O14776|TCRG1_HUMAN | 1.00 |
| 433 | 30.79 | tr|Q8N6N5|Q8N6N5_HUMAN | 15.00 |
| 434 | 2.31 | sp|Q8N1N4|K2C78_HUMAN | 1.00 |
| 435 | 1.55 | tr|A0A024R4Z6_HUMAN | 1.00 |
| 436 | 0.96 | tr|F5H6E2|F5H6E2_HUMAN | 1.00 |
| 437 | 4.12 | tr|A0A024R4G1_HUMAN | 2.00 |
| 438 | 0.99 | sp|P18583|SON_HUMAN | 2.00 |
| 439 | 3.83 | sp|Q9NWK9|BCD1_HUMAN | 2.00 |
| 440 | 1.92 | sp|Q15061|WDR43_HUMAN | 1.00 |
| 441 | 1.50 | sp|O43290|SNUT1_HUMAN | 1.00 |
| 442 | 1.40 | sp|Q9UKV8|AGO2_HUMAN | 1.00 |
| 443 | 2.06 | sp|P55060|XPO2_HUMAN | 2.00 |
| 444 | 4.77 | sp|Q13325|IFIT5_HUMAN | 2.00 |
| 445 | 1.25 | tr|J3QRN6|J3QRN6_HUMAN | 1.00 |
| 446 | 4.19 | tr|Q53HM6|Q53HM6_HUMAN | 2.00 |
| 447 | 2.78 | tr|H7BXH2|H7BXH2_HUMAN | 2.00 |
| 448 | 0.82 | tr|H0Y8C6|H0Y8C6_HUMAN | 1.00 |
| 449 | 2.29 | tr|B7Z3E1|B7Z3E1_HUMAN | 1.00 |
| 450 | 1.16 | tr|Q59FD4|Q59FD4_HUMAN | 1.00 |
| 451 | 5.63 | tr|L0DZ34|L0DZ34_THIND | 1.00 |
| 452 | 2.42 | tr|A8K6Y8|A8K6Y8_HUMAN | 1.00 |
| 453 | 0.98 | tr|Q8N516|Q8N516_HUMAN | 1.00 |
| 454 | 3.51 | sp|Q8WWK9|CKAP2_HUMAN | 2.00 |
| 455 | 4.35 | sp|Q9BVS4|RIOK2_HUMAN | 2.00 |
| 456 | 1.20 | tr|X5DR09|X5DR09_HUMAN | 1.00 |
| 457 | 1.04 | tr|Q9NPK3|Q9NPK3_HUMAN | 1.00 |
| 458 | 3.32 | tr|Q8NCF7|Q8NCF7_HUMAN | 1.00 |
| 459 | 4.78 | tr|B3KPB9|B3KPB9_HUMAN | 2.00 |
| 460 | 0.42 | tr|Q60FE6|Q60FE6_HUMAN | 1.00 |
| 461 | 2.63 | tr|Q59EG8|Q59EG8_HUMAN | 2.00 |
| 462 | 3.81 | sp|Q8IWR0|Z3H7A_HUMAN | 3.00 |
| 463 | 1.52 | tr|A0A024R179_HUMAN | 1.00 |
| 464 | 17.90 | sp|Q5T9A4|ATD3B_HUMAN | 10.00 |
| 465 | 2.11 | tr|Q7Z3C4|Q7Z3C4_HUMAN | 2.00 |
| 466 | 1.61 | tr|A0A090N8G0_HUMAN | 1.00 |
| 467 | 2.79 | tr|B7ZAQ5|B7ZAQ5_HUMAN | 1.00 |
| 468 | 7.75 | tr|M0R210|M0R210_HUMAN | 1.00 |
| 469 | 1.68 | sp|Q92878|RAD50_HUMAN | 2.00 |
| 470 | 7.11 | tr|F8W930|F8W930_HUMAN | 4.00 |
| 471 | 1.79 | tr|Q9HB00|Q9HB00_HUMAN | 1.00 |
| 472 | 8.90 | tr|E9PCX4|E9PCX4_HUMAN | 1.00 |
| 473 | 1.37 | tr|H7BYN4|H7BYN4_HUMAN | 1.00 |
| 474 | 0.46 | sp|Q5D862|FILA2_HUMAN | 1.00 |
| 475 | 1.92 | tr|A0A024QZW3_HUMAN | 1.00 |
| 476 | 32.58 | tr|Q59GL1|Q59GL1_HUMAN | 17.00 |
| 477 | 11.34 | tr|A0A024DAK3_HUMAN | 15.00 |
| 478 | 40.21 | tr|M0QZM1|M0QZM1_HUMAN | 28.00 |
| 479 | 21.48 | tr|Q05CW7|Q05CW7_HUMAN | 11.00 |
| 480 | 18.31 | sp|P16403|H12_HUMAN | 3.00 |
| 481 | 12.84 | tr|A8K7N0|A8K7N0_HUMAN | 2.00 |
| 482 | 0.81 | tr|B4DLT2|B4DLT2_HUMAN | 1.00 |
| 483 | 0.63 | tr|A0A024R9R5_HUMAN | 1.00 |
| 484 | 1.03 | tr|V5YQL4|V5YQL4_HUMAN | 1.00 |
| 485 | 1.24 | tr|Q2L6I2|Q2L6I2_HUMAN | 1.00 |
| 486 | 1.48 | sp|Q9BXP5|SRRT_HUMAN | 1.00 |
| 487 | 4.13 | sp|P62424|RL7A_HUMAN | 1.00 |
| 488 | 0.78 | tr|L0E0P4|L0E0P4_THIND | 1.00 |
| 489 | 4.59 | tr|E7EQL5|E7EQL5_HUMAN | 1.00 |
| 490 | 1.10 | sp|Q13472|TOP3A_HUMAN | 1.00 |
| 491 | 5.42 | tr|Q5QTS3|Q5QTS3_HUMAN | 1.00 |
| 492 | 1.73 | sp|Q9HCD5|NCOA5_HUMAN | 1.00 |
| 493 | 2.24 | sp|Q7Z4V5|HDGR2_HUMAN | 1.00 |
| 494 | 8.22 | sp|P12273|PIP_HUMAN | 1.00 |
| 495 | 1.90 | tr|Q71RH4|Q71RH4_HUMAN | 1.00 |
| 496 | 1.60 | tr|Q5T985|Q5T985_HUMAN | 1.00 |
| 497 | 5.25 | sp|P60174|TPIS_HUMAN | 1.00 |
| 498 | 2.21 | tr|Q8N169|Q8N169_HUMAN | 1.00 |
| 499 | 3.85 | tr|V9HWN7|V9HWN7_HUMAN | 1.00 |
| 500 | 3.93 | tr|Q9BSY0|Q9BSY0_HUMAN | 1.00 |
| 501 | 8.38 | tr|H3BSK4|H3BSK4_HUMAN | 1.00 |
| 502 | 19.05 | tr|C9J0E4|C9J0E4_HUMAN | 1.00 |
| 503 | 2.15 | tr|A0A024R035_HUMAN | 1.00 |
| 504 | 8.61 | sp|P62263|RS14_HUMAN | 1.00 |
| 505 | 1.58 | tr|J3KMX1|J3KMX1_HUMAN | 1.00 |
| 506 | 2.13 | tr|A0A024R4R9_HUMAN | 2.00 |
| 507 | 3.74 | tr|Q53H58|Q53H58_HUMAN | 2.00 |
| 508 | 1.76 | tr|I3L0N3|I3L0N3_HUMAN | 1.00 |
| 509 | 7.09 | tr|V9HWH2|V9HWH2_HUMAN | 2.00 |
| 510 | 2.03 | tr|Q71UA6|Q71UA6_HUMAN | 1.00 |
| 511 | 4.02 | tr|A0A140VK12_HUMAN | 3.00 |
| 512 | 2.62 | tr|V9HVZ0|V9HVZ0_HUMAN | 1.00 |
| 513 | 1.43 | tr|B4E223|B4E223_HUMAN | 1.00 |
| 514 | 2.72 | tr|F8WJN3|F8WJN3_HUMAN | 1.00 |
| 515 | 1.16 | tr|E7EW20|E7EW20_HUMAN | 1.00 |
| 516 | 3.70 | tr|Q96IR1|Q96IR1_HUMAN | 1.00 |
| 517 | 1.35 | tr|A0A024RDU0_HUMAN | 1.00 |
| 518 | 2.35 | tr|Q68DU0|Q68DU0_HUMAN | 2.00 |
| 519 | 2.78 | sp|P23526|SAHH_HUMAN | 1.00 |
| 520 | 2.08 | sp|P49916|DNLI3_HUMAN | 3.00 |
| 521 | 1.48 | RRRRRsp|Q5VXH5|PRAM7_HUMAN | 1.00 |
| 522 | 1.63 | sp|Q96JB3|HIC2_HUMAN | 2.00 |
| 523 | 3.06 | tr|Q05BI1|Q05BI1_HUMAN | 1.00 |
| 524 | 2.50 | tr|B4E106|B4E106_HUMAN | 1.00 |
| 525 | 2.03 | tr|A8K9B9|A8K9B9_HUMAN | 1.00 |
| 526 | 2.30 | sp|Q92615|LAR4B_HUMAN | 1.00 |
| 527 | 1.85 | tr|L0E0R5|L0E0R5_THIND | 1.00 |
| 528 | 6.01 | tr|H0YFN9|H0YFN9_HUMAN | 1.00 |
| 529 | 3.20 | sp|Q9BW92|SYTM_HUMAN | 2.00 |
| 530 | 0.40 | sp|P49792|RBP2_HUMAN | 1.00 |
| 531 | 1.10 | tr|B4DPY2|B4DPY2_HUMAN | 1.00 |
| 532 | 1.59 | tr|A8K8U7|A8K8U7_HUMAN | 1.00 |
| 533 | 0.41 | sp|Q9NU22|MDN1_HUMAN | 2.00 |
| 534 | 11.83 | sp|P05109|S10A8_HUMAN | 1.00 |
| 535 | 1.27 | tr|B4DN49|B4DN49_HUMAN | 1.00 |
| 536 | 1.27 | tr|V9HWI4|V9HWI4_HUMAN | 1.00 |
| 537 | 2.77 | tr|A0A024R1K8_HUMAN | 2.00 |
| 538 | 0.97 | sp|Q8IWV7|UBR1_HUMAN | 1.00 |
| 539 | 1.78 | sp|Q12894|IFRD2_HUMAN | 1.00 |
| 540 | 1.68 | tr|A0A024R994_HUMAN | 1.00 |
| 541 | 2.49 | tr|Q6IT96|Q6IT96_HUMAN | 1.00 |
| 542 | 6.84 | tr|L0E0M9|L0E0M9_THIND | 1.00 |
| 543 | 5.51 | tr|F6T1Q0|F6T1Q0_HUMAN | 2.00 |
| 544 | 2.50 | tr|H3BS72|H3BS72_HUMAN | 1.00 |
| 545 | 1.54 | sp|Q5VYS8|TUT7_HUMAN | 2.00 |
| 546 | 1.66 | tr|B3KNK5|B3KNK5_HUMAN | 1.00 |
| 547 | 1.46 | sp|Q9BVQ7|SPA5L_HUMAN | 1.00 |
| 548 | 7.35 | tr|Q53G25|Q53G25_HUMAN | 1.00 |
| 549 | 3.31 | tr|V9HWB9|V9HWB9_HUMAN | 1.00 |
| 550 | 4.50 | tr|A8K5Q1|A8K5Q1_HUMAN | 2.00 |
| 551 | 2.63 | tr|B1Q2B0|B1Q2B0_HUMAN | 1.00 |
| 552 | 1.41 | tr|B3KN05|B3KN05_HUMAN | 1.00 |
| 553 | 38.10 | tr|A0N4V7|A0N4V7_HUMAN | 1.00 |
| 554 | 0.61 | tr|E7EVA0|E7EVA0_HUMAN | 1.00 |
| 555 | 0.98 | tr|B4DET0|B4DET0_HUMAN | 1.00 |
| 556 | 1.79 | tr|B2R935|B2R935_HUMAN | 1.00 |
| 557 | 0.99 | tr|E9PDF6|E9PDF6_HUMAN | 1.00 |
| 558 | 1.10 | sp|P35606|COPB2_HUMAN | 1.00 |
| 559 | 1.05 | tr|A8K9T9|A8K9T9_HUMAN | 1.00 |
| 560 | 2.94 | sp|Q96GM8|TOE1_HUMAN | 1.00 |
| 561 | 0.68 | tr|Q4LE58|Q4LE58_HUMAN | 1.00 |
| 562 | 0.66 | sp|Q8NDV7|TNR6A_HUMAN | 1.00 |
| 563 | 1.77 | tr|G3XAG1|G3XAG1_HUMAN | 1.00 |
| 564 | 2.52 | tr|A8KAQ5|A8KAQ5_HUMAN | 1.00 |
| 565 | 2.20 | tr|H0YN14|H0YN14_HUMAN | 2.00 |
| 566 | 11.34 | tr|A0A0A0MRQ5_HUMAN | 1.00 |
| 567 | 3.04 | tr|A0A087WXU3_HUMAN | 2.00 |
| 568 | 1.29 | sp|O00268|TAF4_HUMAN | 1.00 |
| 569 | 1.35 | sp|P12270|TPR_HUMAN | 4.00 |
| 570 | 0.88 | tr|Q308M6|Q308M6_HUMAN | 1.00 |
| 571 | 2.03 | tr|A0A024R704_HUMAN | 1.00 |
| 572 | 6.25 | tr|A0A024R8D7_HUMAN | 1.00 |
| 573 | 2.07 | tr|Q8IYG2|Q8IYG2_HUMAN | 1.00 |
| 574 | 1.76 | sp|Q5TAA0|TTC22_HUMAN | 1.00 |
| 575 | 1.36 | tr|F8W1F5|F8W1F5_HUMAN | 1.00 |
| 576 | 12.05 | sp|O43719|HTSF1_HUMAN | 9.00 |
| 577 | 1.20 | tr|Q6UUU9|Q6UUU9_HUMAN | 1.00 |
| 578 | 1.22 | tr|A0A0C4DFM2_HUMAN | 1.00 |
| 579 | 3.23 | tr|I6L965|I6L965_HUMAN | 1.00 |
| 580 | 2.27 | tr|Q59ET3|Q59ET3_HUMAN | 1.00 |
| 581 | 0.71 | tr|J3KN36|J3KN36_HUMAN | 1.00 |
| 582 | 0.42 | tr|Q9NYZ5|Q9NYZ5_HUMAN | 1.00 |
| 583 | 2.76 | RRRRRsp|Q5VV42|CDKAL_HUMAN | 1.00 |
| 584 | 2.99 | sp|Q2NL82|TSR1_HUMAN | 2.00 |
| 585 | 0.95 | sp|Q8IY17|PLPL6_HUMAN | 1.00 |
| 586 | 7.41 | tr|L0R5A1|L0R5A1_HUMAN | 1.00 |
| 587 | 1.71 | tr|Q6NUR7|Q6NUR7_HUMAN | 1.00 |
| 588 | 2.97 | tr|A8K525|A8K525_HUMAN | 1.00 |
| 589 | 1.81 | sp|Q02413|DSG1_HUMAN | 1.00 |
| 590 | 1.21 | tr|B0AZS5|B0AZS5_HUMAN | 1.00 |
| 591 | 1.63 | tr|A8K3Q7|A8K3Q7_HUMAN | 1.00 |
| 592 | 1.17 | tr|B4DGC5|B4DGC5_HUMAN | 1.00 |
| 593 | 1.32 | tr|A8K0P8|A8K0P8_HUMAN | 1.00 |
| 594 | 1.32 | sp|Q8IWA0|WDR75_HUMAN | 1.00 |
| 595 | 2.98 | tr|B4E228|B4E228_HUMAN | 1.00 |
| 596 | 1.55 | tr|Q59GW5|Q59GW5_HUMAN | 1.00 |
| 597 | 1.27 | tr|Q53GZ2|Q53GZ2_HUMAN | 1.00 |
| 598 | 8.22 | tr|Q32Q12|Q32Q12_HUMAN | 1.00 |
| 599 | 2.02 | sp|Q96HR8|NAF1_HUMAN | 1.00 |
| 600 | 1.36 | tr|Q6I7N8|Q6I7N8_HUMAN | 1.00 |
| 601 | 2.04 | tr|A0A1U9X8Q1_HUMAN | 1.00 |
| 602 | 2.05 | RRRRRtr|B1AMR4|B1AMR4_HUMAN | 1.00 |
| 603 | 1.72 | sp|Q5QJE6|TDIF2_HUMAN | 1.00 |
| 604 | 2.52 | tr|L0DZG9|L0DZG9_THIND | 1.00 |
| 605 | 1.50 | sp|Q14694|UBP10_HUMAN | 1.00 |
| 606 | 4.40 | tr|J3KPE3|J3KPE3_HUMAN | 1.00 |
| 607 | 1.28 | sp|P46977|STT3A_HUMAN | 1.00 |
| 608 | 0.63 | tr|A8K583|A8K583_HUMAN | 1.00 |
| 609 | 0.96 | tr|B0AZM4|B0AZM4_HUMAN | 1.00 |
| 610 | 1.77 | tr|Q5THR1|Q5THR1_HUMAN | 1.00 |
| 611 | 1.23 | tr|Q8TA92|Q8TA92_HUMAN | 1.00 |
| 612 | 1.31 | tr|A0A024R4A5_HUMAN | 1.00 |
| 613 | 7.77 | tr|Q0VAS5|Q0VAS5_HUMAN | 1.00 |
| 614 | 1.14 | tr|Q4LE60|Q4LE60_HUMAN | 1.00 |
| 615 | 1.79 | tr|A0A024R3R5_HUMAN | 1.00 |
| 616 | 0.69 | sp|Q9UHB7|AFF4_HUMAN | 1.00 |
| 617 | 0.70 | sp|Q7KZ85|SPT6H_HUMAN | 1.00 |
| 618 | 2.28 | tr|B3KRY3|B3KRY3_HUMAN | 1.00 |
| 619 | 0.95 | RRRRRtr|L0DW34|L0DW34_THIND | 1.00 |
| 620 | 1.78 | sp|Q8IXI1|MIRO2_HUMAN | 1.00 |
| 621 | 7.69 | tr|A8K517|A8K517_HUMAN | 1.00 |
| 622 | 4.10 | tr|Q71VH6|Q71VH6_HUMAN | 1.00 |
| 623 | 0.96 | sp|O15397|IPO8_HUMAN | 1.00 |
| 624 | 0.29 | sp|O95071|UBR5_HUMAN | 1.00 |
| 625 | 1.51 | tr|A0A0B4J1V8_HUMAN | 1.00 |
| 626 | 3.46 | tr|F2Z393|F2Z393_HUMAN | 1.00 |
| 627 | 0.99 | RRRRRtr|A0A126LAW9|A0A126LAW9_HUMAN | 1.00 |
| 628 | 0.94 | tr|A8K940|A8K940_HUMAN | 1.00 |
| 629 | 3.68 | tr|Q16094|Q16094_HUMAN | 1.00 |
| 630 | 1.18 | tr|I3L2Z9|I3L2Z9_HUMAN | 1.00 |
| 631 | 4.51 | tr|C9J5H8|C9J5H8_HUMAN | 1.00 |
| 632 | 4.93 | sp|Q96M42|CU129_HUMAN | 1.00 |
| 633 | 3.73 | tr|Q59FF0|Q59FF0_HUMAN | 3.00 |
| 634 | 2.06 | tr|Q53QN0|Q53QN0_HUMAN | 1.00 |
| 635 | 6.92 | tr|Q08AJ9|Q08AJ9_HUMAN | 1.00 |
| 636 | 0.67 | tr|B4DYG5|B4DYG5_HUMAN | 1.00 |
| 637 | 1.72 | tr|B4DLG2|B4DLG2_HUMAN | 1.00 |
| 638 | 0.00 | sp|Q9Y6X9|MORC2_HUMAN | 1.00 |
| 639 | 1.89 | tr|Q96CS0|Q96CS0_HUMAN | 1.00 |
| 640 | 1.57 | tr|B2RCN8|B2RCN8_HUMAN | 1.00 |
| 641 | 3.06 | sp|Q1ED39|KNOP1_HUMAN | 1.00 |
| 642 | 0.66 | tr|B7ZLC9|B7ZLC9_HUMAN | 1.00 |
| 643 | 0.72 | RRRRRtr|B9A6K8|B9A6K8_HUMAN | 1.00 |
| 644 | 3.28 | tr|Q96II5|Q96II5_HUMAN | 2.00 |
| 645 | 1.52 | tr|L0DWZ1|L0DWZ1_THIND | 1.00 |
| 646 | 0.42 | RRRRRtr|D3DQQ7|D3DQQ7_HUMAN | 1.00 |
| 647 | 1.55 | sp|Q9P0U3|SENP1_HUMAN | 1.00 |
| 648 | 0.71 | RRRRRsp|O15040|TCPR2_HUMAN | 1.00 |
| 649 | 1.16 | sp|O95373|IPO7_HUMAN | 1.00 |
| 650 | 1.61 | RRRRRsp|Q8NFI3|ENASE_HUMAN | 1.00 |
| 651 | 1.71 | sp|O75342|LX12B_HUMAN | 1.00 |
| 652 | 4.00 | RRRRRtr|Q5T182|Q5T182_HUMAN | 1.00 |
| 653 | 5.17 | tr|Q59GE4|Q59GE4_HUMAN | 1.00 |
| 654 | 1.48 | tr|A0A1W2PNX8_HUMAN | 2.00 |
| 655 | 0.68 | tr|B7Z8Z8|B7Z8Z8_HUMAN | 1.00 |
| 656 | 2.55 | tr|B7Z592|B7Z592_HUMAN | 1.00 |
| 657 | 2.30 | sp|Q02040|AK17A_HUMAN | 2.00 |
| 658 | 1.45 | tr|L0E106|L0E106_THIND | 1.00 |
| **^#^659** | **3.59** | **tr|A0A0J9YYJ9|BCL11A_HUMAN** | **1.00** |
| 660 | 3.70 | tr|Q53G83|Q53G83_HUMAN | 1.00 |
| 661 | 2.38 | tr|B5BUK7|B5BUK7_HUMAN | 1.00 |
| 662 | 0.48 | tr|A0A024R7A6_HUMAN | 1.00 |
| 663 | 0.61 | RRRRRsp|A6NNT2|CP096_HUMAN | 1.00 |
| 664 | 2.03 | RRRRRtr|A0A024R157|A0A024R157_HUMAN | 1.00 |
| 665 | 0.99 | RRRRRsp|Q5T848|GP158_HUMAN | 1.00 |
| 666 | 2.64 | sp|Q15323|K1H1_HUMAN | 1.00 |
| 667 | 1.29 | tr|B2RBJ7|B2RBJ7_HUMAN | 1.00 |
| 668 | 2.57 | RRRRRtr|A0A126GWD6|A0A126GWD6_HUMAN | 1.00 |
| 669 | 1.04 | tr|Q59H19|Q59H19_HUMAN | 1.00 |
| 670 | 30.77 | tr|F8W6S9|F8W6S9_HUMAN | 1.00 |
| 671 | 0.50 | RRRRRtr|A0A0A0MRB5|A0A0A0MRB5_HUMAN | 1.00 |
| 672 | 1.21 | RRRRRsp|P0DOX6|IGM_HUMAN | 1.00 |
| 673 | 4.22 | RRRRRtr|B7Z3P2|B7Z3P2_HUMAN | 1.00 |
| 674 | 2.04 | RRRRRsp|Q9H972|CN093_HUMAN | 1.00 |
| 675 | 0.89 | sp|Q14126|DSG2_HUMAN | 1.00 |
| 676 | 1.23 | RRRRRsp|Q14526|HIC1_HUMAN | 1.00 |
| 677 | 0.97 | tr|V9HWK2|V9HWK2_HUMAN | 1.00 |
| 678 | 2.43 | sp|Q08257|QOR_HUMAN | 1.00 |
| 679 | 0.32 | sp|P21675|TAF1_HUMAN | 1.00 |
| 680 | 7.81 | tr|B4DE59|B4DE59_HUMAN | 4.00 |
| 681 | 2.31 | tr|Q59GM9|Q59GM9_HUMAN | 2.00 |
| 682 | 0.44 | tr|G8JLP4|G8JLP4_HUMAN | 1.00 |
| 683 | 0.83 | RRRRRsp|O43283|M3K13_HUMAN | 1.00 |
| 684 | 0.84 | RRRRRtr|A8QI98|A8QI98_HUMAN | 1.00 |
| 685 | 0.96 | tr|A0A0X1KG71_HUMAN | 1.00 |
| 686 | 2.50 | RRRRRtr|A2RQR4|A2RQR4_HUMAN | 1.00 |
| 687 | 4.15 | tr|F8W1U3|F8W1U3_HUMAN | 1.00 |
| 688 | 1.16 | tr|J3KNR0|J3KNR0_HUMAN | 1.00 |
| 689 | 16.60 | tr|O95036|O95036_HUMAN | 4.00 |
| 690 | 2.53 | tr|L0E266|L0E266_THIND | 1.00 |
| 691 | 0.27 | tr|E7ESW6|E7ESW6_HUMAN | 1.00 |
| 692 | 22.31 | tr|A0A1U9X7X9_HUMAN | 14.00 |
| 693 | 0.34 | RRRRRtr|A0A0A0MSP7|A0A0A0MSP7_HUMAN | 1.00 |
| 694 | 22.31 | tr|B4DWU6|B4DWU6_HUMAN | 12.00 |

**^#^**SWI/SNF family members, such as SMARCA4, SMCA5, SMARCA3, ACTB and BCL11A，are highlighted in bold.

**Table S10: 109 common genes down-regualted by PRMT1-knockdown and SMARCA4-knockdown in HCT116 cells**

For the study of differential gene expression, Genespring (version13.1, Agilent Technologies) were employed to finish the basic analysis with the raw data. The genes with a fold change value greater than 1.5, and a p-value < 0.01 were considered differentially expressed. FC, fold change.
