# Supplementary material for: PRMT1-mediated H4R3me2a recruits SMARCA4 to promote colorectal cancer progression by enhancing EGFR signaling
Source: Genome Med. 2021 Apr 14;13:58. doi: 10.1186/s13073-021-00871-5 (PMC8048298; doi:10.1186/s13073-021-00871-5)

Additional file 3: Scans of the uncropped blots for Western blots.

Fig. S1. Uncropped blots for Western blots in Fig.1.

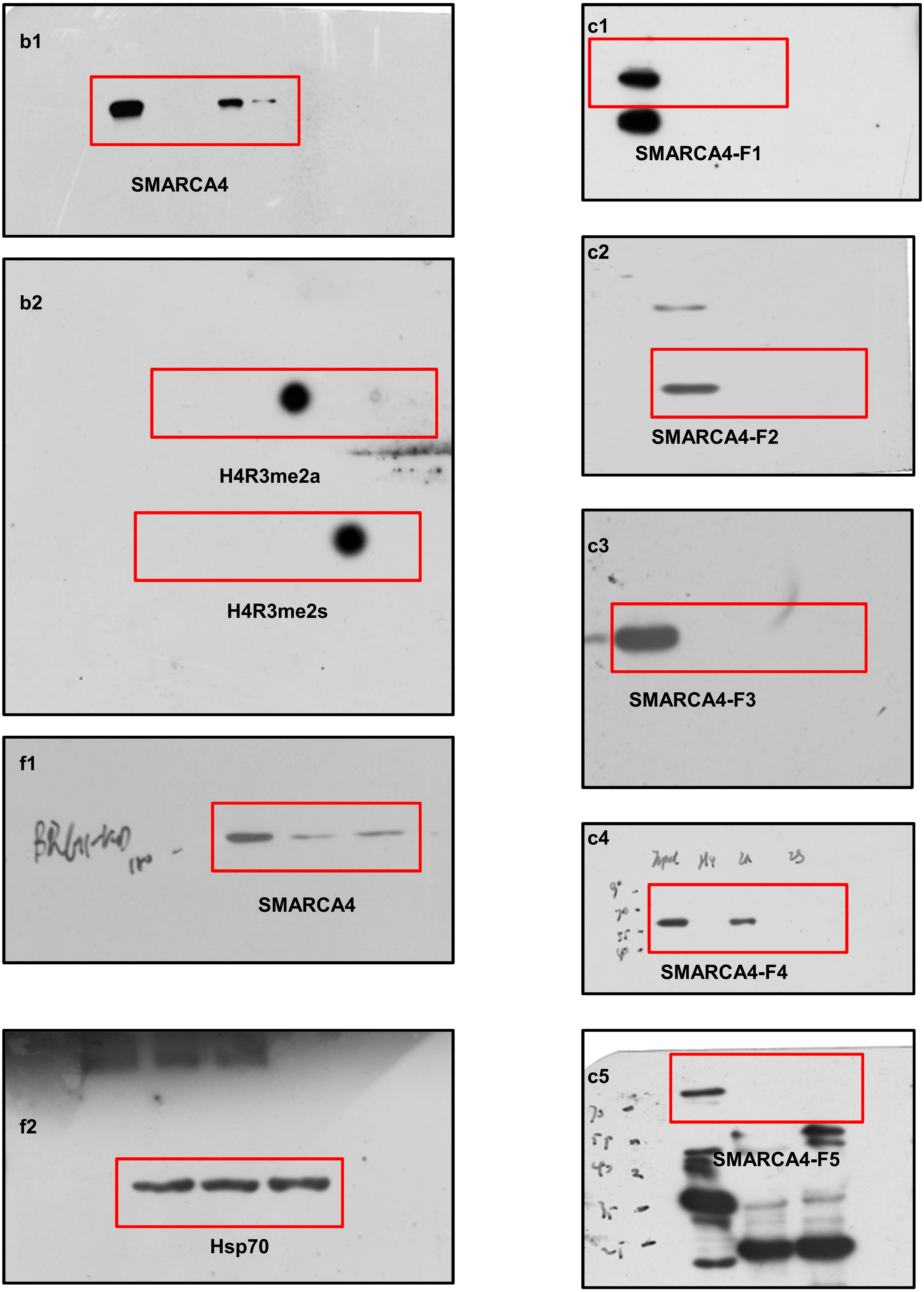

Fig. S2. Uncropped blots for Western blots in Fig. 2.

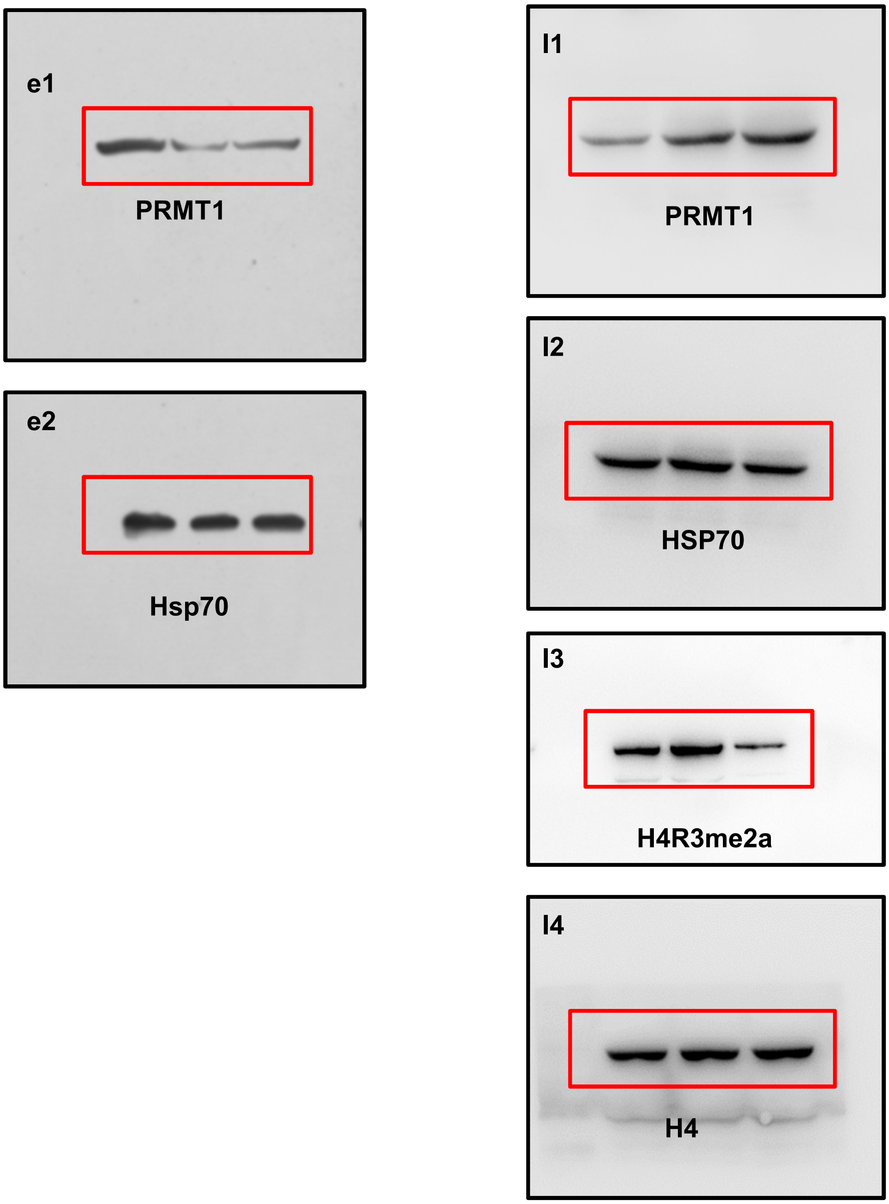

Fig. S3. Uncropped blots for Western blots in Fig. 3.

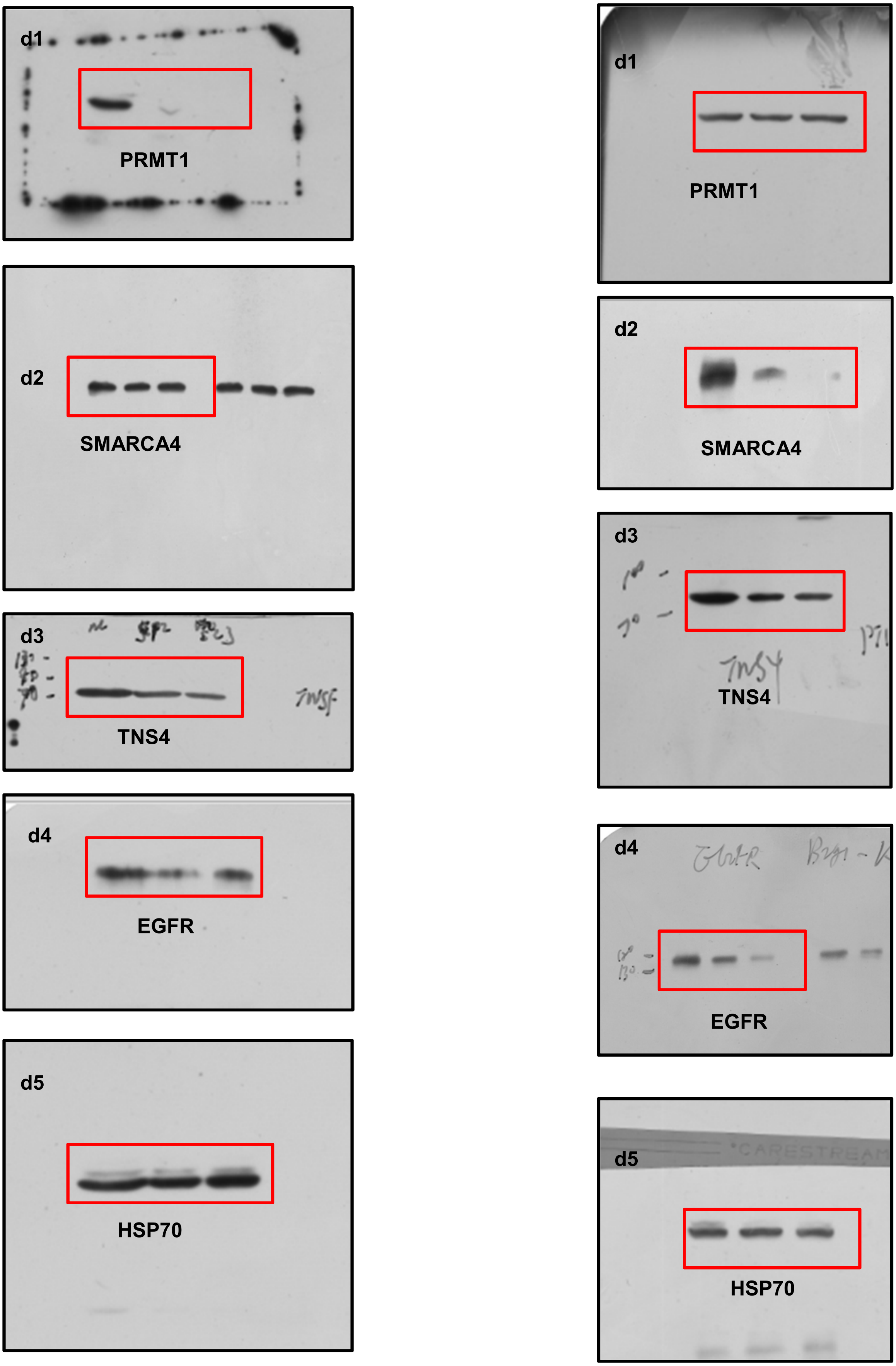

Fig. S4. Uncropped blots for Western blots in Fig. 4.

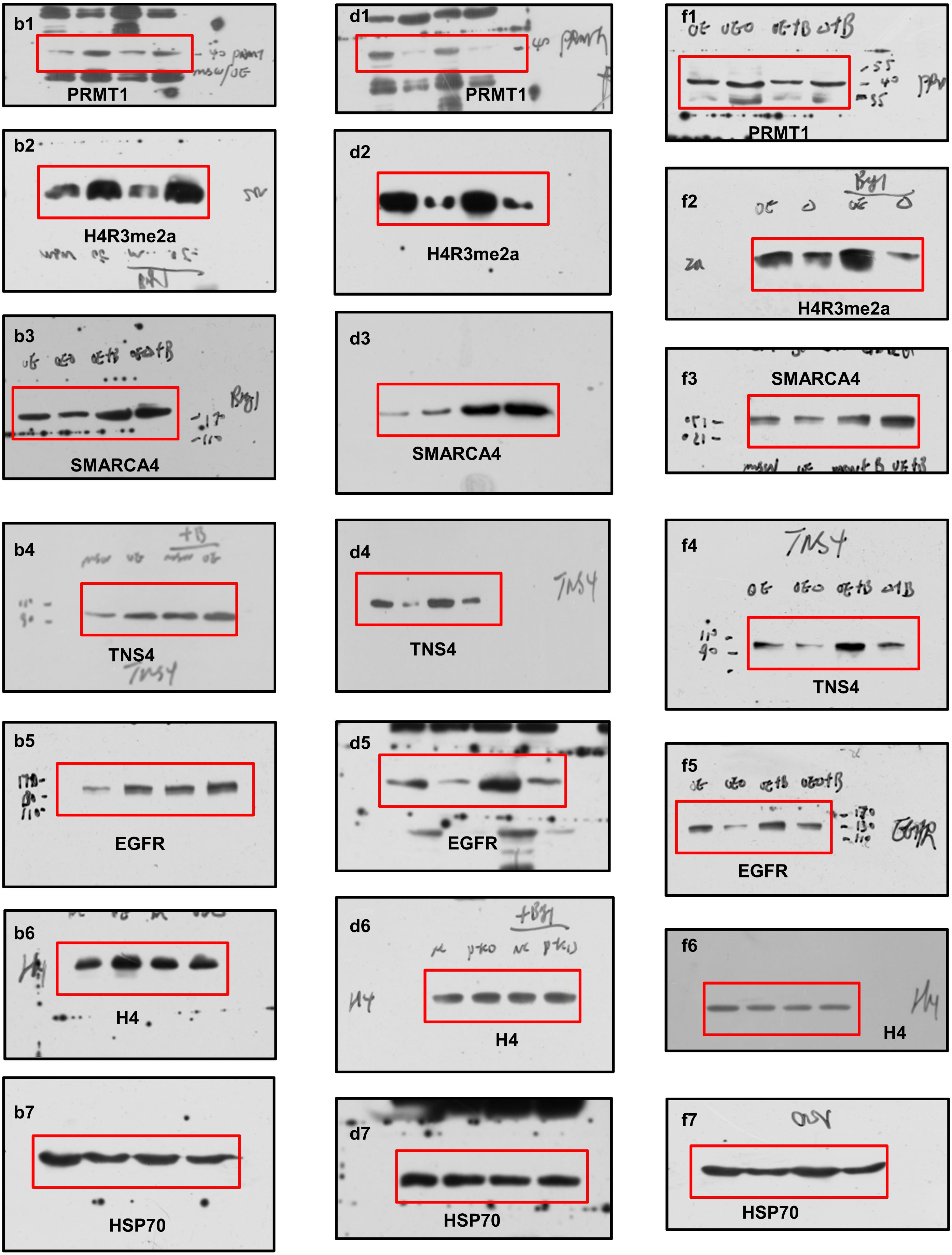

Fig. S5. Uncropped blots for Western blots in Fig. 5.

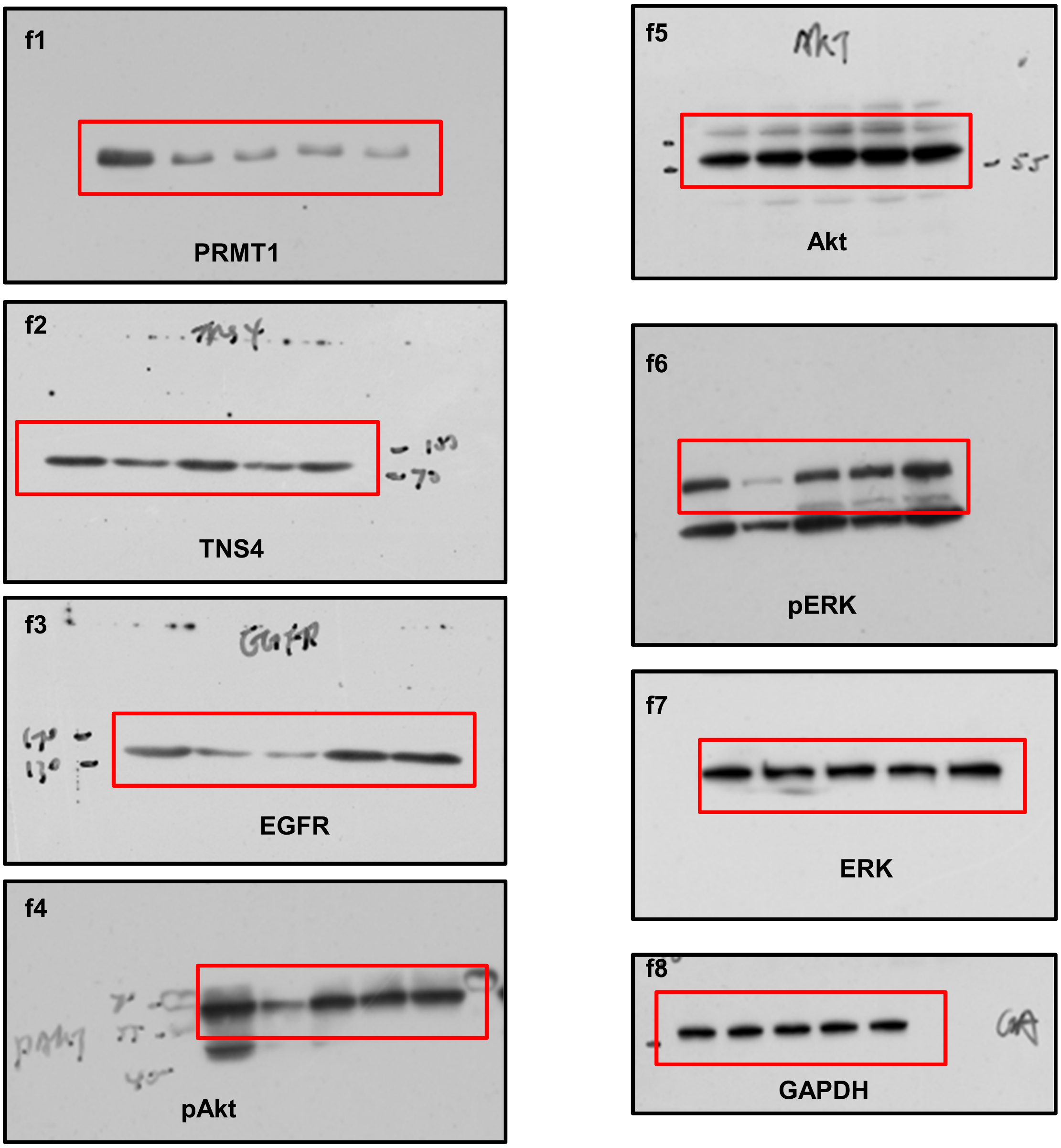

Fig. S5. Uncropped blots for Western blots in Fig. 5.

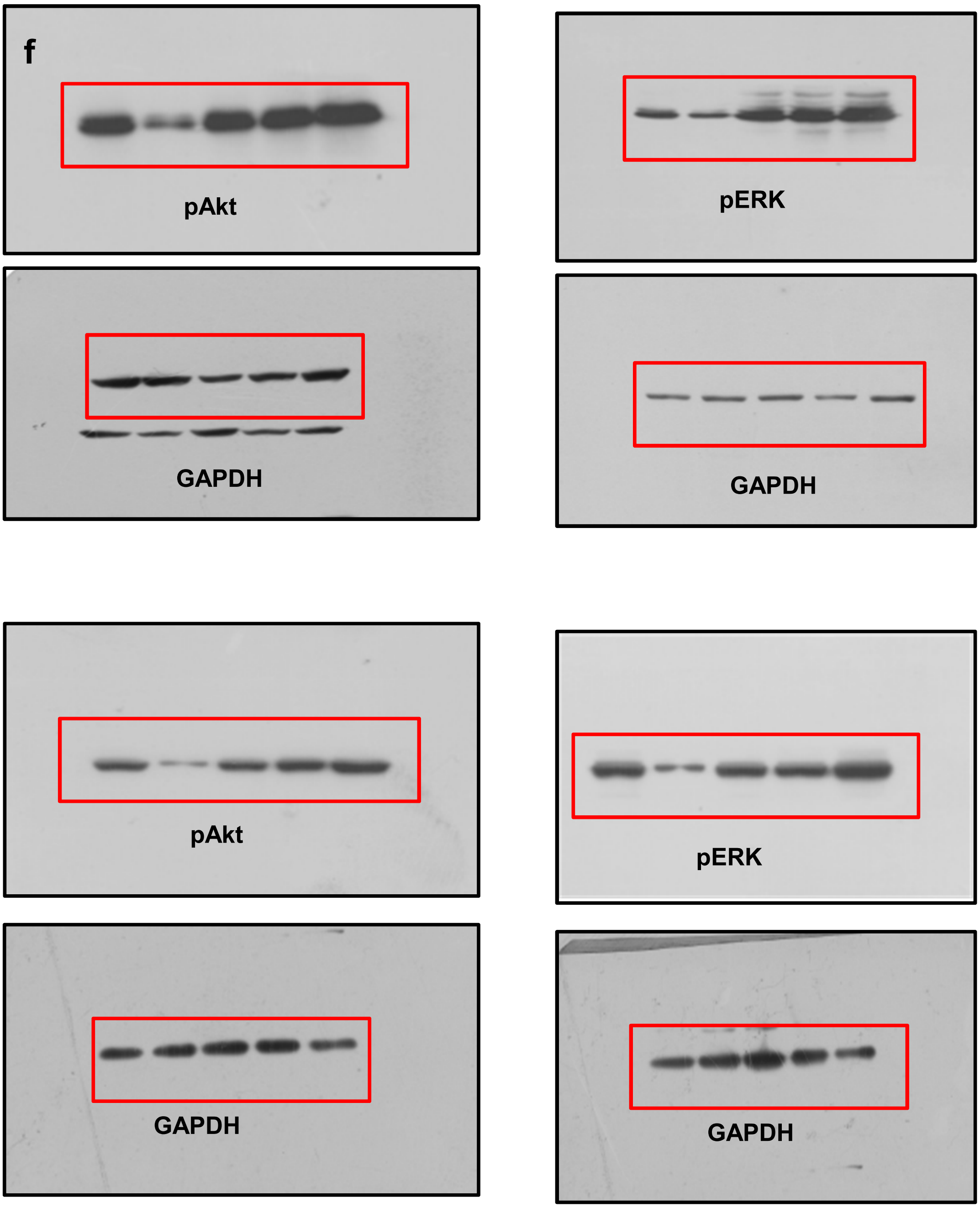

Fig. S6. Uncropped blots for Western blots in Fig. 6.

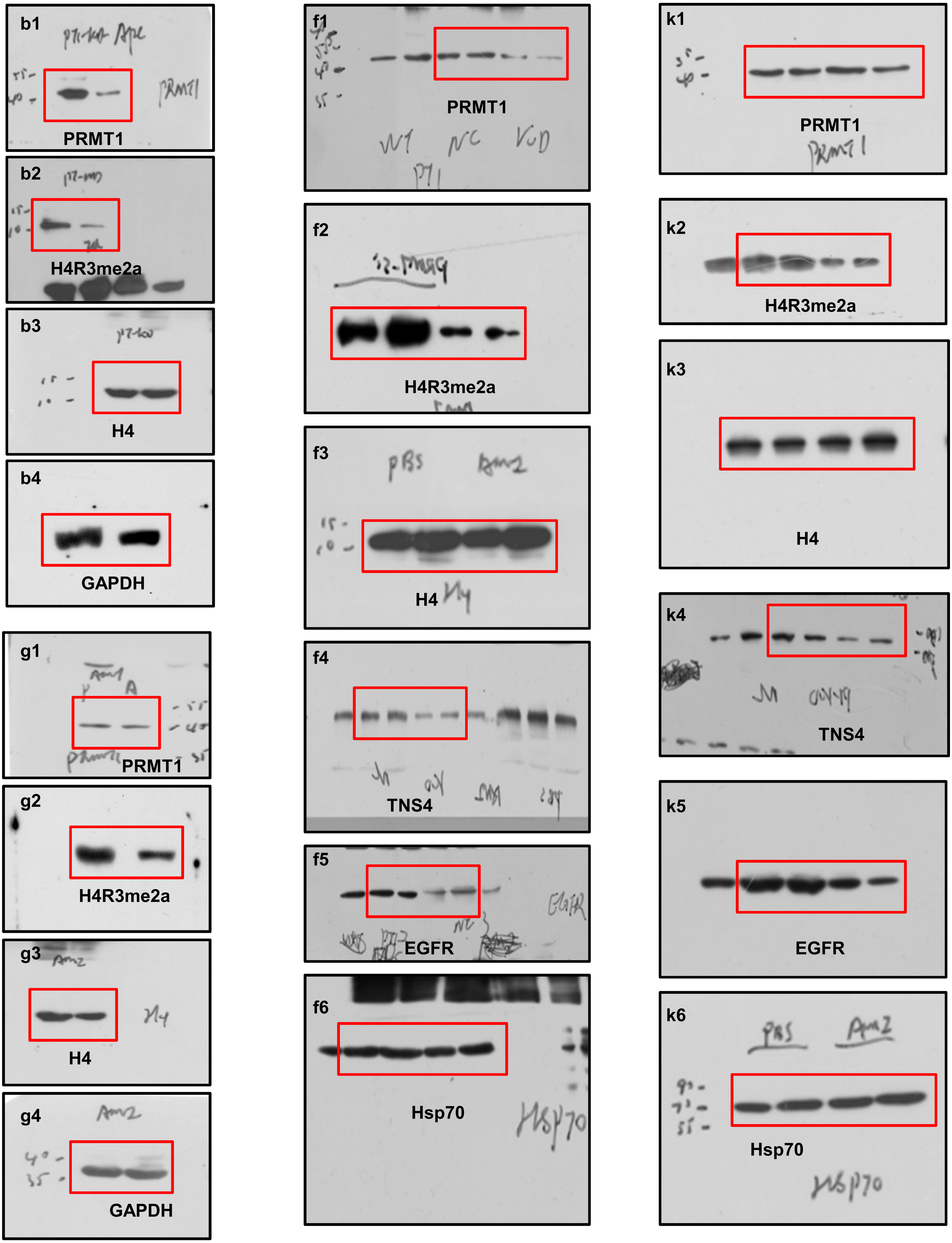

Fig. S7. Uncropped blots for Western blots in Additional file 1: Fig. S1.

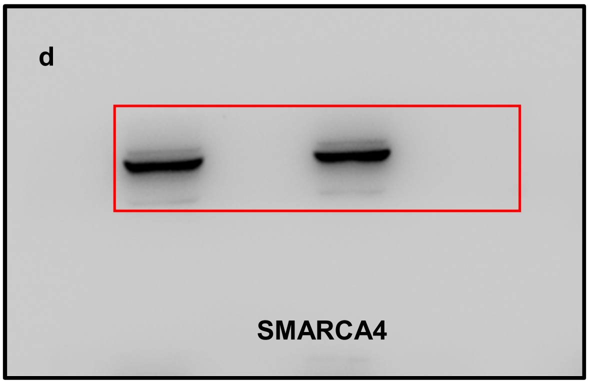

Fig. S8. Uncropped blots for Western blots in Additional file 1: Fig. S3.

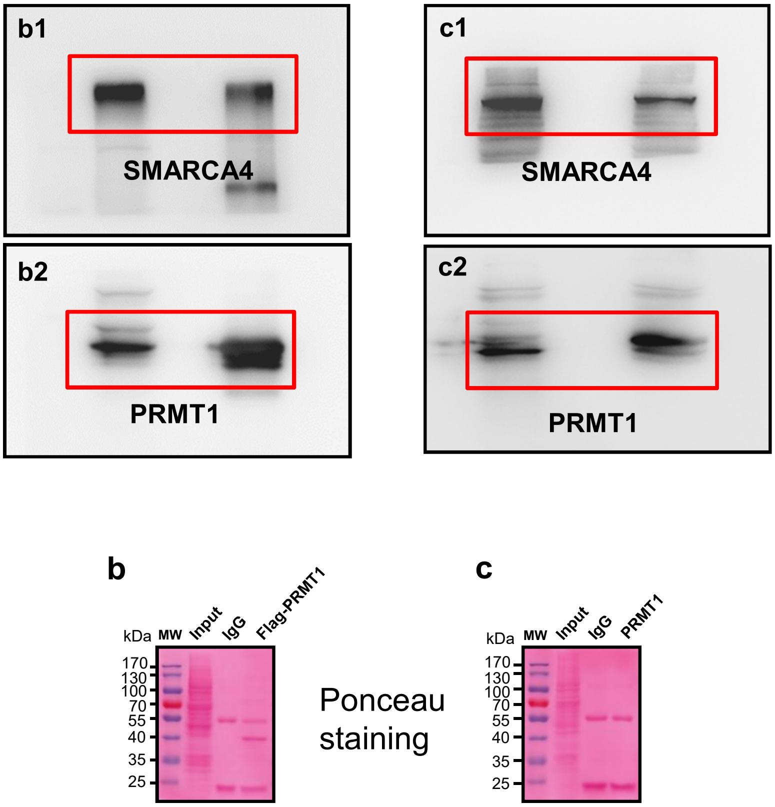

Fig. S9. Uncropped blots for Western blots in Additional file 1: Fig. S6.

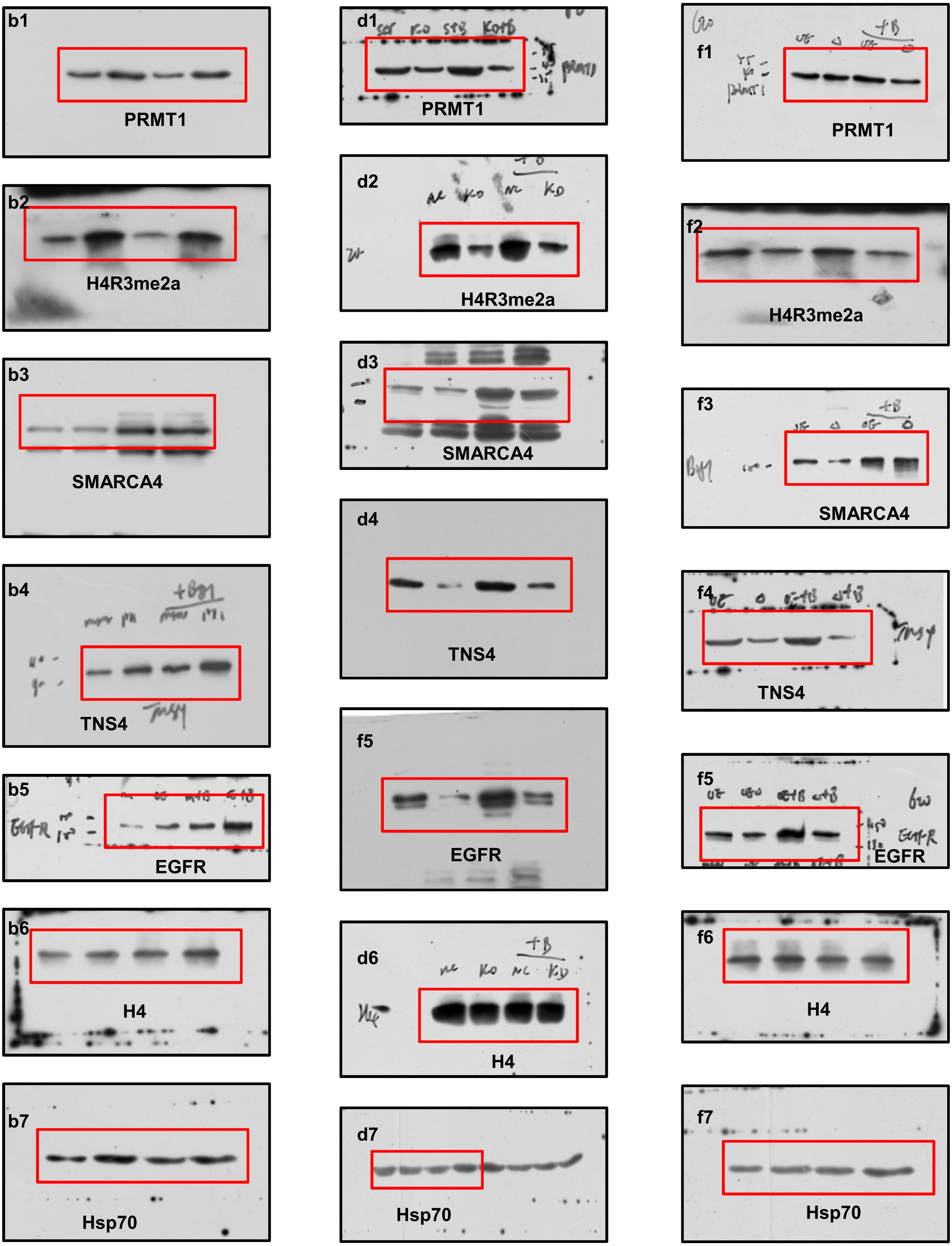

Fig. S9. Uncropped blots for Western blots in Additional file 1: Fig. S6.

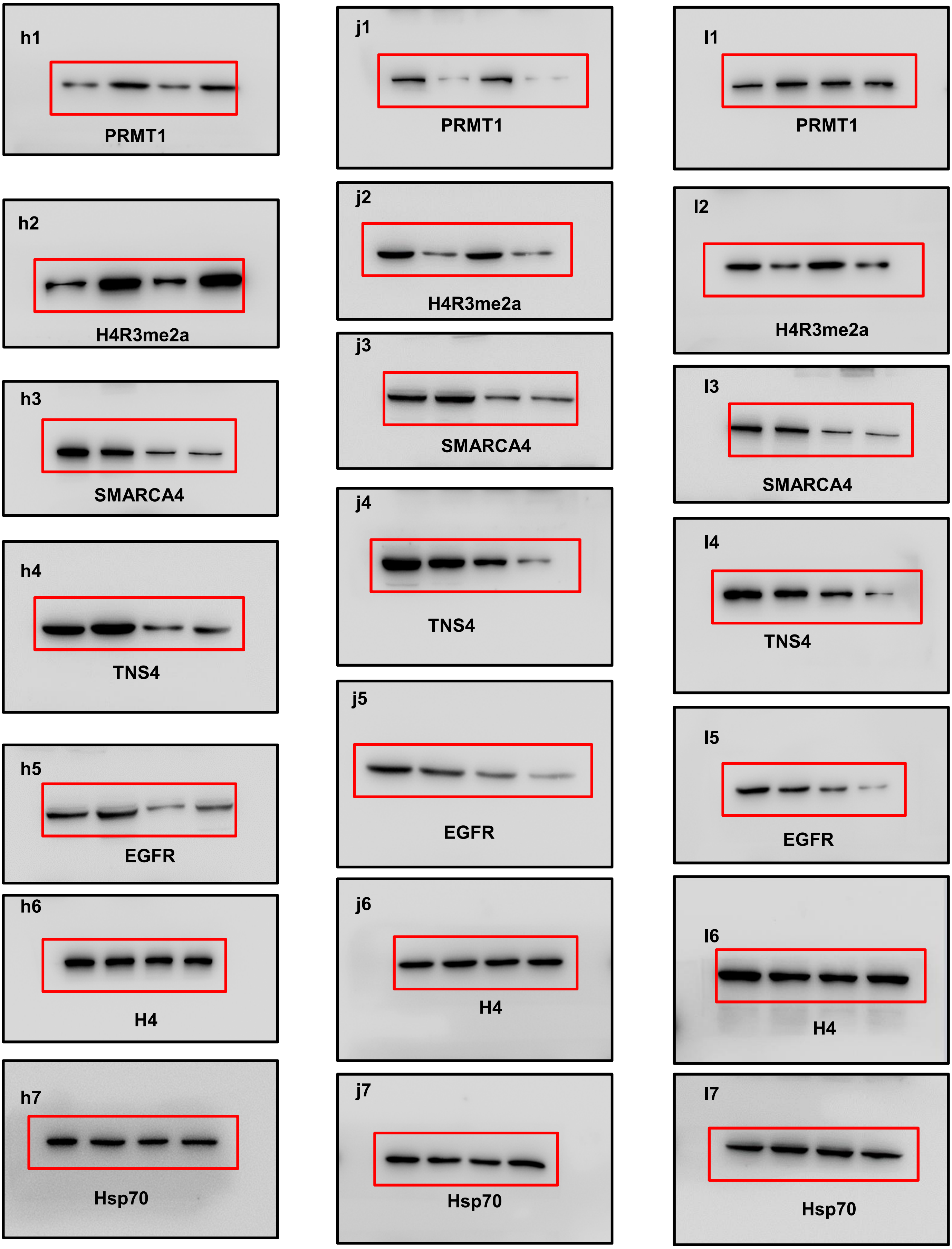

Fig. S10. Uncropped blots for Western blots in Additional file 1: Fig. S8.

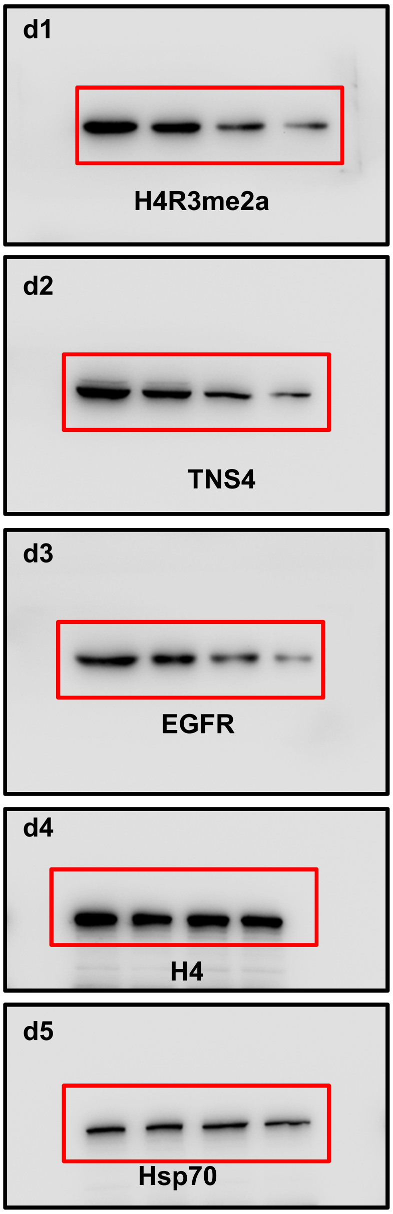

Supplement: Supplementary file 3 — Additional file 3. Scans of the uncropped blots for Western blots. Fig. S1. Uncropped blots for Western blots in Fig. 1. Fig. S2. Uncropped blots for Western blots in Fig. 2. Fig. S3. Uncropped blots for Western blots in Fig. 3. Fig. S4. Uncropped blots for Western blots in Fig. 4. Fig. S5. Uncropped blots for Western blots in Fig. 5. Fig. S6. Uncropped blots for Western blots in Fig. 6. Fig. S7. Uncropped blots for Western blots in Additional file 1: Fig. S1. Fig. S8. Uncropped blots for Western blots in Additional file 1: Fig. S3. Fig. S9. Uncropped blots for Western blots in Additional file 1: Fig. S6. Fig. S10. Uncropped blots for Western blots in Additional file 1: Fig. S8. [file 13073_2021_871_MOESM3_ESM.pdf]
